# Supplementary material for: Hydroarylation of olefins catalysed by a dimeric ytterbium(II) alkyl
Source: Nat Commun. 2021 May 25;12:3147. doi: 10.1038/s41467-021-23444-x (PMC8149703; doi:10.1038/s41467-021-23444-x)
Supplement: Supplementary file 1 — Supplementary Information [file 41467_2021_23444_MOESM1_ESM.docx]

Supplementary Information for

**Hydroarylation of Olefins Catalysed by a Dimeric Ytterbium(II) Alkyl**

Georgia Richardson,^1^ Iskander Douair,^2^ Scott A. Cameron,^3^ Joe Bracegirdle,^1^ Robert A. Keyzers,^1^ Michael S. Hill,*^4^ Laurent Maron*^2^ and Mathew D. Anker*^1^

[1] School of Chemical and Physical Sciences, Victoria University of Wellington, PO Box 600, Wellington 6012, New Zealand. Email: [Mathew.Anker@vuw.ac.nz](mailto:Mathew.Anker@vuw.ac.nz)

[2] Université de Toulouse et CNRS, INSA, UPS, UMR 5215, LPCNO 135 Avenue de Rangueil, 31077 Toulouse (France) E-mail: [laurent.maron@irsamc.ups-tlse.fr](mailto:laurent.maron@irsamc.ups-tlse.fr)

[3] Ferrier Research Institute, Victoria University of Wellington, PO Box 600, Wellington 6012, New Zealand

[4] Department of Chemistry, University of Bath, Bath, BA2 7AY, UK E-mail: msh27@bath.ac.uk

[1. Materials and Methods 4](#_Toc53061093)

[1.1 Experimental Procedures 4](#_Toc53061094)

[1.2 Synthetic, Spectroscopic and Analytic Data for New Compounds 5](#_Toc53061095)

[[HC{(Me)CN(2,6-^i^Pr_2_C_6_H_3_)}_2_YbN(SiMe_3_)_2_]_4_ (**1**) 5](#_Toc53061096)

[**Supplementary Figure 1:** ^1^H NMR spectrum (500 MHz, C_6_D_6_) of [HC{(Me)CN(2,6-^i^Pr_2_C_6_H_3_)}_2_YbN(SiMe_3_)_2_]_4_ (**1**). 6](#_Toc53061097)

[**Supplementary Figure 2:** ^13^C{^1^H} NMR spectrum (126 MHz, C_6_D_6_) of [HC{(Me)CN(2,6-^i^Pr_2_C_6_H_3_)}_2_YbN(SiMe_3_)_2_]_4_ (**1**). 7](#_Toc53061098)

[**Supplementary Figure 3:** ^1^H – ^13^C HSQC NMR spectrum of [HC{(Me)CN(2,6-^i^Pr_2_C_6_H_3_)}_2_YbN(SiMe_3_)_2_]_4_ (**1**). 8](#_Toc53061099)

[**Supplementary Figure 4:** ORTEP representation (ellipsoid 30% probability) of [HC{(Me)CN(2,6-^i^Pr_2_C_6_H_3_)}_2_YbN(SiMe_3_)_2_]_4_ (**1**). 9](#_Toc53061100)

[[HC{(Me)CN(2,6-^i^Pr_2_C_6_H_3_)}_2_YbH]_2_ (2) 10](#_Toc53061101)

[**Supplementary Figure 5:** ^1^H NMR spectrum (500 MHz, C_6_D_6_) of [HC{(Me)CN(2,6-^i^Pr_2_C_6_H_3_)}_2_YbH]_2_ (**2**). 11](#_Toc53061102)

[**Supplementary Figure 6:** ^1^H NMR spectrum (500 MHz, C_6_D_6_) of [HC{(Me)CN(2,6-^i^Pr_2_C_6_H_3_)}_2_YbH]_2_ (**2**), Expansion of the downfield region of the spectra shown in Supplementary Figure 5, showing the two triplet resonances indicative of two hydride species in the solution state in a 1:25 ratio. 12](#_Toc53061103)

[**Supplementary Figure 7:** ^13^C{^1^H} NMR spectrum (126 MHz, C_6_D_6_) of [HC{(Me)CN(2,6-^i^Pr_2_C_6_H_3_)}_2_YbH]_2_ (**2**). 13](#_Toc53061104)

[**Supplementary Figure 8:** ^1^H – ^13^C HSQC NMR spectrum of [HC{(Me)CN(2,6-^i^Pr_2_C_6_H_3_)}_2_YbH]_2_ (**2**). 14](#_Toc53061105)

[**Supplementary Figure 9:** ^1^H – ^1^H EXSY NMR spectrum of [HC{(Me)CN(2,6-^i^Pr_2_C_6_H_3_)}_2_YbH]_2_ (**2**), showing exchange between **2** and **2’**. 15](#_Toc53061106)

[**Supplementary Figure 10:** ^2^H NMR spectrum (77 MHz, C_6_D_6_) of [HC{(Me)CN(2,6-^i^Pr_2_C_6_H_3_)}_2_YbD]_2_ (**2-*d***). 16](#_Toc53061107)

[**Supplementary Figure 11:** ORTEP representation (ellipsoid 30% probability) of [HC{(Me)CN(2,6-^i^Pr_2_C_6_H_3_)}_2_YbH]_2_ (**2**). 17](#_Toc53061108)

[[HC{(Me)CN(2,6-^i^Pr_2_C_6_H_3_)}_2_Yb(C_2_H_5_)]_2_ (3) 18](#_Toc53061109)

[**Supplementary Figure 12:** ^1^H NMR spectrum (500 MHz, C_6_D_6_) of [HC{(Me)CN(2,6-^i^Pr_2_C_6_H_3_)}_2_Yb(C_2_H_5_)]_2_ (**3**). 19](#_Toc53061110)

[**Supplementary Figure 13:** ^13^C{^1^H} NMR spectrum (126 MHz, C_6_D_6_) of [HC{(Me)CN(2,6-^i^Pr_2_C_6_H_3_)}_2_Yb(C_2_H_5_)]_2_ (**3**) 20](#_Toc53061111)

[**Supplementary Figure 14:** ^1^H – ^13^C HSQC NMR spectrum of [HC{(Me)CN(2,6-^i^Pr_2_C_6_H_3_)}_2_Yb(C_2_H_5_)]_2_ (**3**). 21](#_Toc53061112)

[**Supplementary Figure 15:** ORTEP representation (ellipsoid 30% probability) of [HC{(Me)CN(2,6-^i^Pr_2_C_6_H_3_)}_2_Yb(C_2_H_5_)]_2_ (**3**). 22](#_Toc53061113)

[**Supplementary Figure 16:** ^1^H NMR spectrum (500 MHz, C_6_D_6_) of [HC{(Me)CN(2,6-^i^Pr_2_C_6_H_3_)}_2_Yb(C_3_H_7_)]_2_ (**4**). 24](#_Toc53061114)

[**Supplementary Figure 18:** ^1^H – ^13^C HSQC NMR spectrum (500 MHz, C_6_D_6_) of [HC{(Me)CN(2,6-^i^Pr_2_C_6_H_3_)}_2_Yb(C_3_H_7_)]_2_ (**4**). 26](#_Toc53061115)

[1.3 Reactions of compounds 3 and 4 with *d*_6_-benzene 28](#_Toc53061116)

[Reaction of compound 3 with C_6_D_6_ 28](#_Toc53061117)

[Reaction of compound 4 with C_6_D_6_ 31](#_Toc53061118)

[1.4 Hydroarylation of ethene or propene with *d*_6_-benzene/benzene 34](#_Toc53061119)

[Hydroarylation of ethene with C_6_D_6_ catalysed by 2 34](#_Toc53061120)

[Hydroarylation of ethene with C_6_D_6_ catalysed by 2 at 40 ^o^C 43](#_Toc53061121)

[Hydroarylation of ethene with C_6_H_6_ catalysed by 2 at 40 ^o^C 53](#_Toc53061122)

[Hydroarylation of propene with C_6_D_6_ catalysed by 2 54](#_Toc53061123)

[Hydroarylation of propene with C_6_H_6_ catalysed by 2 at 40 ^o^C 63](#_Toc53061124)

[Hydroarylation of propene with C_6_H_6_ catalysed by 2 64](#_Toc53061125)

[1.5 Crystallography 65](#_Toc53061126)

[**Supplementary Table 1**. Crystal data and structure refinement for [Yb{N(SiMe_3_)_2_}_2_]_2_, **1** and **2**. 67](#_Toc53061127)

[**Supplementary Table 2.** Crystal data and structure refinement for **3**, **4** and **6**. 68](#_Toc53061128)

[1.6 Computational details 71](#_Toc53061129)

[**Figure 62:** Computed enthalpy profile for the alkylation of benzene catalysed by **2**, including the possibility of polymerising ethene. 71](#_Toc53061130)

[1.7 Supplementary References 148](#_Toc53061131)

# 1. Materials and Methods

## 1.1 Experimental Procedures

All manipulations were performed under a dry, oxygen-free argon atmosphere using standard Schlenk-line techniques, or in a conventional nitrogen-filled glovebox. Solvents were dried over appropriate drying agents and degassed prior to use. NMR spectra were recorded using a JEOL 500 MHz spectrometer, operating at 500 MHz (^1^H), 126 MHz (^13^C) or 77 MHz (^2^H). Spectra were recorded at 298 K (unless stated otherwise) and proton and carbon chemical shifts were referenced internally to residual solvent resonances. Coupling constants are quoted in Hz. Elemental analyses were performed at Elemental Microanalysis Ltd (UK). Phenylsilane (97%), ethene (>99.5%) and propene (>99%) were purchased from Sigma-Aldrich Ltd. and used without further purification. [Yb{N(SiMe_3_)_2_}_2_]_2_ and [HC{(Me)CN(2,6-^i^Pr_2_C_6_H_3_)}_2_]H were synthesised by literature procedures.^1,2^ All other chemicals were purchased from Sigma-Aldrich and used without further purification. GC-MS analyses were carried out using a Shimadzu QP2010-Plus gas chromatograph−mass spectrometer equipped with an AOC-20i auto injector. The samples were taken from the NMR tube and diluted 20:1 with chloroform. GC-MS analyses used helium as the carrier gas. Mass spectra were obtained at 70 eV in positive ion mode, scanning at *m/z* 40−600 every 0.3 s. The ion source was held at 200 °C, while the MS-transfer line was at 305 °C. Compound identity was determined using both retention time and mass spectral fragmentation patterns. Samples were introduced (1 *μ*L) into a glass split/splitless liner at 270 °C. Separations were performed using a Restek RXI-5Sil-MS column (30 m × 0.25 mm × 0.25 *μ*m) with a 50:1 split injection using constant carrier gas flow (linear velocity 43.4 cm/s; 1.38 mL/min). The initial oven temperature was 50 °C, held for 2 min, after which a temperature ramp of 10 °C/min to 300 °C was used, with a final hold of 5 min (total analysis time 32 min).

## 1.2 Synthetic, Spectroscopic and Analytic Data for New Compounds

### Preparation of [HC{(Me)CN(2,6-^i^Pr_2_C_6_H_3_)}_2_YbN(SiMe_3_)_2_]_4_ (1)

A colourless toluene solution of the pre-ligand BDI^Dipp^H (847 mg, 2.02 mmol) was added to an orange toluene solution of Yb(HMDS)_2_ (1000 mg, 2.02 mmol) in an ampoule fitted with a J. Youngs tap and sealed. The mixture was heated to 100 ^o^C for 16h. The solvent was removed *in vacuo* from the resulting dark red solution to give **1** as an analytically pure dark red crystalline solid in essentially quantitative yields. Deep red crystals suitable for X-ray diffraction analysis were obtained from a saturated toluene solution at – 30 ^o^C.

^1^H NMR (500 MHz, C_6_D_6_) δ 7.20-7.00 (m, 6H, Ar*H*), 4.88 (s, 1H, NC(CH_3_)C*H*), 3.29 (sept, *J* = 6.9 Hz, 4H, C*H*(CH_3_)_2_), 1.73 (s, 6H, NC(C*H*_3_)CH), 1.32 (d, *J* = 6.9 Hz, 12H, CH(C*H*_3_)_2_), 1.28 (d, *J* = 6.9 Hz, 12H, CH(C*H*_3_)_2_), 0.11 (s, 18H, Si(C*H*_3_)_3_).

^13^C{^1^H} NMR (126 MHz, C_6_D_6_) δ 165.7 (N*C*(CH_3_)CH), 146.7 (*C*_ipso_), 141.1 (*C*_ortho_), 125.0 (*C*_para_), 124.2 (*C*_meta_), 91.6 (NC(CH_3_)*C*H), 28.6 (*C*H(CH_3_)_2_), 25.8, 25.5 (CH(*C*H_3_)_2_), 24.7 (NC(*C*H_3_)CH), 5.5 (Si(*C*H_3_)_3_).

Elemental Analysis; Calculated: C, 55.97; H, 7.92; N, 5.59; Found: C, 55.76; H, 7.62; N, 5.16

#### **Supplementary Figure 1:** ^1^H NMR spectrum (500 MHz, C_6_D_6_) of [HC{(Me)CN(2,6-^i^Pr_2_C_6_H_3_)}_2_YbN(SiMe_3_)_2_]_4_ (**1**).

####

#### **Supplementary Figure 2:** ^13^C{^1^H} NMR spectrum (126 MHz, C_6_D_6_) of [HC{(Me)CN(2,6-^i^Pr_2_C_6_H_3_)}_2_YbN(SiMe_3_)_2_]_4_ (**1**).

#### **Supplementary Figure 3:** ^1^H – ^13^C HSQC NMR spectrum of [HC{(Me)CN(2,6-^i^Pr_2_C_6_H_3_)}_2_YbN(SiMe_3_)_2_]_4_ (**1**).


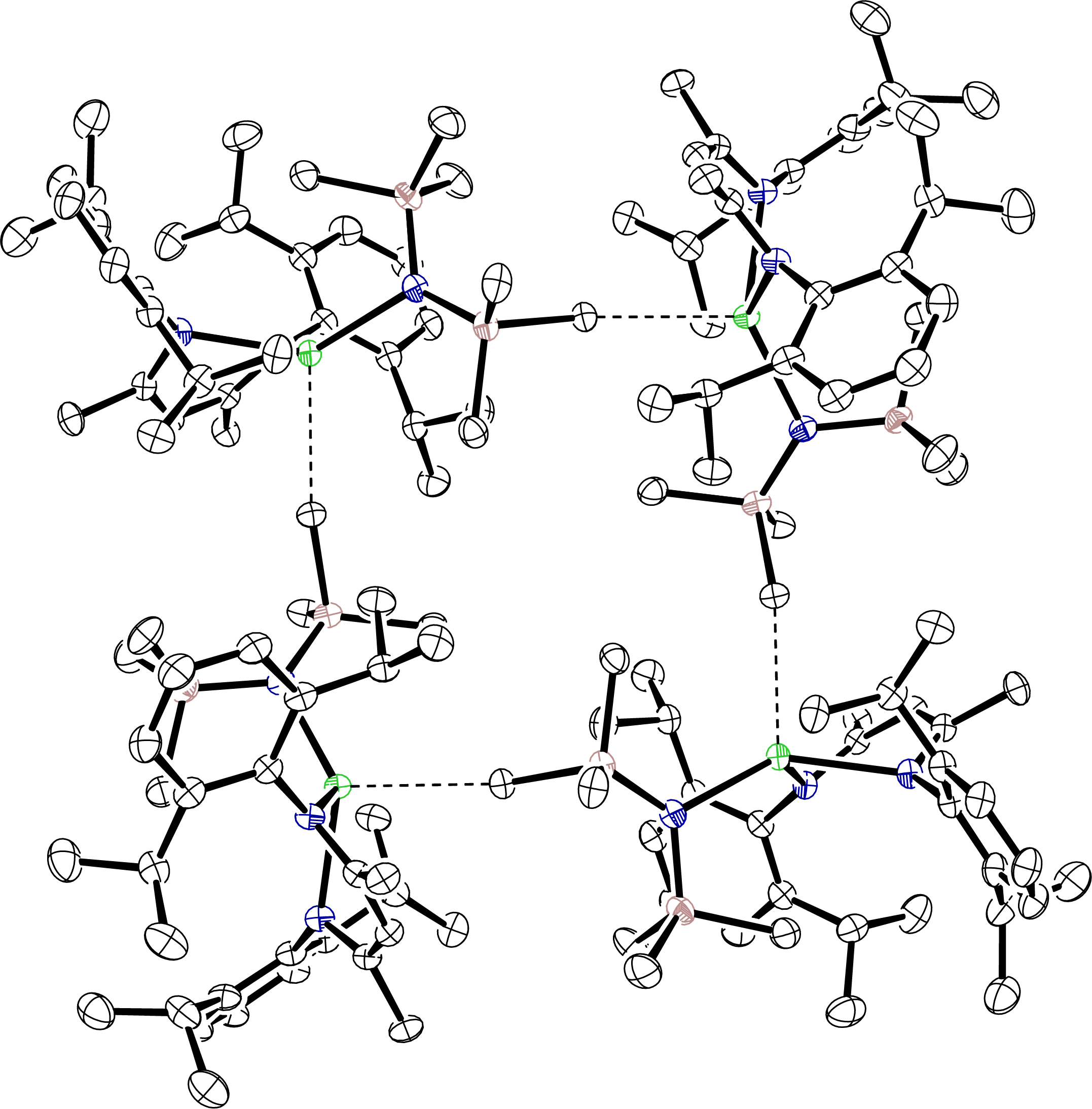


#### **Supplementary Figure 4:** ORTEP representation (ellipsoid 30% probability) of [HC{(Me)CN(2,6-^i^Pr_2_C_6_H_3_)}_2_YbN(SiMe_3_)_2_]_4_ (**1**). Hydrogen atoms have been omitted for clarity.

### Preparation of [HC{(Me)CN(2,6-^i^Pr_2_C_6_H_3_)}_2_YbH]_2_ (2)

Inside a glovebox, a colourless toluene solution containing 3 equivalents of phenylsilane (129.4 mg, 1.19 mmol) was added to a scintillation vial containing a dark red toluene solution of **1** (300 mg, 0.39mmol) and left to stir at room temperature for 30 minutes which resulted in a black solution. Solvent was removed *in vacuo* to give the crude product as a thick black paste, which was re-dissolved into hexane to give a black solution. The solution was filtered, concentrated and crystallised at –30 ^o^C overnight to give **2** as black hexagonal block-like crystals (179 mg, 77%).

^1^H NMR (500 MHz, C_6_D_6_) δ 7.93 (t, *J* = 398 Hz, 1H, Yb*H*), 7.01 (d, *J* = 7.6 Hz, 4H, Ar*H*), 6.52 (t, *J* = 7.6 Hz, 2H, Ar*H*), 4.84 (s, 1H, NC(CH_3_)C*H*), 3.14 (sept, *J* = 6.8 Hz, 4H, C*H*(CH_3_)_2_), 1.68 (s, 6H, NC(C*H*_3_)CH), 1.34 (d, *J* = 6.8 Hz, 12H, CH(C*H*_3_)_2_), 1.12 (d, *J* = 6.8 Hz, 12H, CH(C*H*_3_)_2_).

^13^C{^1^H} NMR (126 MHz, C_6_D_6_) δ 163.1 (N*C*(CH_3_)CH), 147.0 (*C*_ipso_), 143.6 (*C*_ortho_), 123.5 (*C*_para_), 123.1 (*C*_meta_), 95.0 (NC(CH_3_)*C*H), 29.1 (*C*H(CH_3_)_2_), 25.2, 25.1 (CH(*C*H_3_)_2_), 24.2 (NC(*C*H_3_)CH).

Elemental Analysis: Calculated: C, 58.87; H, 7.15; N, 4.73; Found: C, 55.03; H, 6.62; N, 4.22;

Despite repeated attempts, more accurate microanalysis could not be obtained for this compound.

#### **Supplementary Figure 5:** ^1^H NMR spectrum (500 MHz, C_6_D_6_) of [HC{(Me)CN(2,6-^i^Pr_2_C_6_H_3_)}_2_YbH]_2_ (**2**).

#### **Supplementary Figure 6:** ^1^H NMR spectrum (500 MHz, C_6_D_6_) of [HC{(Me)CN(2,6-^i^Pr_2_C_6_H_3_)}_2_YbH]_2_ (**2**), Expansion of the downfield region of the spectra shown in Supplementary Figure 5, showing the two triplet resonances indicative of two hydride species in the solution state in a 1:25 ratio.

####

#### **Supplementary Figure 7:** ^13^C{^1^H} NMR spectrum (126 MHz, C_6_D_6_) of [HC{(Me)CN(2,6-^i^Pr_2_C_6_H_3_)}_2_YbH]_2_ (**2**).

#### **Supplementary Figure 8:** ^1^H – ^13^C HSQC NMR spectrum of [HC{(Me)CN(2,6-^i^Pr_2_C_6_H_3_)}_2_YbH]_2_ (**2**).

#### **Supplementary Figure 9:** ^1^H – ^1^H EXSY NMR spectrum of [HC{(Me)CN(2,6-^i^Pr_2_C_6_H_3_)}_2_YbH]_2_ (**2**), showing the exchange between **2** and **2′**.

#### **Supplementary Figure 10:** ^2^H NMR spectrum (77 MHz, C_6_D_6_) of [HC{(Me)CN(2,6-^i^Pr_2_C_6_H_3_)}_2_YbD]_2_ (**2-*d***).

#### **Supplementary Figure 11:** ORTEP representation (ellipsoid 30% probability) of [HC{(Me)CN(2,6-^i^Pr_2_C_6_H_3_)}_2_YbH]_2_ (**2**). Hydrogen atoms except hydrides (H1 and H1a) have been omitted for clarity.

### Preparation of [HC{(Me)CN(2,6-^i^Pr_2_C_6_H_3_)}_2_Yb(C_2_H_5_)]_2_ (3)

In a J. Youngs tap NMR tube, a black hexane solution of **2** (74 mg, 0.06 mmol) was degassed *via* three freeze-pump-thaw cycles before being exposed to 1 atmosphere of ethene and left at room temperature for 16h. The hexane solution was decanted from the resulting black – brown crystalline solid which was dried *in vacuo* to give **3** (31 mg, 42% isolated yield) as a black solid. Black – brown crystals suitable for X-ray diffraction analysis were obtained from a 100:1 hexane: toluene solution of **3** at – 30 ^o^C.

^1^H NMR (500 MHz, C_6_D_6_) δ 7.15 - 7.11 (m, 6H, Ar*H*), 4.66 (s, 1H, NC(CH_3_)C*H*), 3.19 (sept, *J* = 6.9 Hz, 4H, C*H*(CH_3_)), 1.52 (s, 6H, NC(C*H*_3_)CH), 1.38 (t, *J* = 8.1 Hz, 3H, YbCH_2_C*H*_3_) 1.17 (d, *J* = 6.9 Hz, 12H, CH(C*H*_3_)_2_), 1.03 (d, *J* = 6.9 Hz, 12H, CH(C*H*_3_)_2_), -0.37 (q, *J* = 8.1 Hz, 2H, YbC*H*_2_CH_3_).

^13^C{^1^H} NMR (126 MHz, C_6_D_6_) δ165.6 (NC(CH_3_)C*H)*, 145.3 (C_ipso_), 142.1 (C_ortho_), 124.8 (C_para_), 124.0 (C_meta_), 94.5 (NC(CH_3_)*C*H), 28.4 (*C*H(CH_3_)_2_), 24.9, 24.8 (CH(*C*H_3_)_2_), 24.5 (NC(*C*H_3_)CH), 15.8 (YbCH_2_*C*H_3_).

Elemental Analysis: Calculated: C, 60.08; H, 7.48; N, 4.52; Found: C, 52.49; H, 6.52; N, 3.96;

Despite repeated attempts, more accurate microanalysis could not be obtained for this compound.

#### **Supplementary Figure 12:** ^1^H NMR spectrum (500 MHz, C_6_D_6_) of [HC{(Me)CN(2,6-^i^Pr_2_C_6_H_3_)}_2_Yb(C_2_H_5_)]_2_ (**3**).

#### **Supplementary Figure 13:** ^13^C{^1^H} NMR spectrum (126 MHz, C_6_D_6_) of [HC{(Me)CN(2,6-^i^Pr_2_C_6_H_3_)}_2_Yb(C_2_H_5_)]_2_ (**3**)

#### **Supplementary Figure 14:** ^1^H – ^13^C HSQC NMR spectrum of [HC{(Me)CN(2,6-^i^Pr_2_C_6_H_3_)}_2_Yb(C_2_H_5_)]_2_ (**3**).

#### **Supplementary Figure 15:** ORTEP representation (ellipsoid 30% probability) of [HC{(Me)CN(2,6-^i^Pr_2_C_6_H_3_)}_2_Yb(C_2_H_5_)]_2_ (**3**). Hydrogen atoms except those of the bridging ethyl groups have been omitted for clarity.

**Preparation of [HC{(Me)CN(2,6-^i^Pr_2_C_6_H_3_)}_2_Yb(C_3_H_7_)]_2_ (4)**

In a J. Youngs tap NMR tube, a black hexane solution of **2** (49 mg, 0.0414 mmol) was degassed *via* three freeze-pump-thaw cycles before being exposed to 1 atmosphere of propene and left at room temperature for 16h. The hexane solution was decanted from the resulting black – brown crystalline solid which was dried *in vacuo* to give **4** (34 mg, 64% isolated yield) as a black solid. Black – brown crystals suitable for X-ray diffraction analysis were obtained from a 100:1 hexane: toluene solution of **4** at – 30 ^o^C.

^1^H NMR (500 MHz, C_6_D_6_) δ 7.20 – 7.17 (m, 4H, Ar*H*), 7.14 – 7.11 (m, 2H, Ar*H*), 4.67 (s, 1H, NC(CH_3_)C*H*), 3.21 (sept, *J* = 6.8 Hz, 4H, C*H*(CH_3_)), 1.52 (s, 6H, NC(C*H*_3_)CH), 1.18 (d, *J* = 6.8 Hz, 12H, CH(C*H*_3_)_2_), 1.14 (m, 5H, YbCH_2_C*H*_2_CH_3_ overlapping YbCH_2_CH_2_C*H*_3_), 1.05 (d, *J* = 6.8 Hz, 12H, CH(C*H*_3_)_2_), -0.29 (t, *J* = 8.8 Hz, 2H, YbC*H*_2_CH_2_CH_3_).

^13^C{^1^H} NMR (126 MHz, C_6_D_6_) δ165.7 (N*C*(CH_3_)CH*)*, 145.4 (C_ipso_), 142.0 (C_ortho_), 124.8 (C_para_), 124.0 (C_meta_), 94.3 (NC(CH_3_)*C*H), 28.4 (*C*H(CH_3_)_2_), 24.9, 24.8 (CH(*C*H_3_)_2_), 24.5 (NC(*C*H_3_)CH), 23.0 (YbCH_2_CH_2_*C*H_3_)

Elemental Analysis: Calculated: C, 60.64; H, 7.63; N, 4.42; Found: C, 59.21; H, 7.59; N, 4.39.

#### **Supplementary Figure 16:** ^1^H NMR spectrum (500 MHz, C_6_D_6_) of [HC{(Me)CN(2,6-^i^Pr_2_C_6_H_3_)}_2_Yb(C_3_H_7_)]_2_ (**4**).

**Supplementary Figure 17:** ^13^C{^1^H} NMR spectrum (126 MHz, C_6_D_6_) of [HC{(Me)CN(2,6-^i^Pr_2_C_6_H_3_)}_2_Yb(C_3_H_7_)]_2_ (**4**).

#### **Supplementary Figure 18:** ^1^H – ^13^C HSQC NMR spectrum (500 MHz, C_6_D_6_) of [HC{(Me)CN(2,6-^i^Pr_2_C_6_H_3_)}_2_Yb(C_3_H_7_)]_2_ (**4**).

**Supplementary Figure 19:** ORTEP representation (ellipsoid 30% probability) of [HC{(Me)CN(2,6-^i^Pr_2_C_6_H_3_)}_2_Yb(C_3_H_7_)]_2_ (**4**) (^1^ = 1 – x, – y, – z). Hydrogen atoms except those of the bridging *n*-propyl groups have been omitted for clarity. Selected bond lengths [Å] and angles [°]: Yb1A-N1 2.3394(16), Yb1A-N2 2.3619(16), Yb1A-C31 2.486(2), Yb1A-C31^1^ 2.598(2), Yb1A-C32^1^ 2.830(3), Yb1B-C31 2.035(3), Yb1B-N2 2.379(2), Yb1B-N1 2.381(2), Yb1B-C31^1^ 3.002(3), C31-C32 1.538(3), C71-C72 1.549(3). N1-Yb1A-N2 81.85(6), N1-Yb1A-C31 104.45(7), N2-Yb1A C31 115.86(7), N1-Yb1A-C311 131.83(7), N2-Yb1A-C31^1^ 124.24(7), C31-Yb1A-C31^1^ 98.54(7), N1-Yb1A-C321 109.48(7), N2-Yb1A-C32^1^ 103.45(7), C31-Yb1A-C32^1^ 130.83(8), C31^1^-Yb1A-C32^1^ 32.55(7), C31-Yb1B-N2 136.96(15), C31-Yb1B-N1 119.32(12), N2-Yb1B-N1 80.63(7), C31-Yb1B-C31^1^ 97.96(9), N2-Yb1B-C31^1^ 108.61(11), N1-Yb1B-C31^1^ 113.26(12).

## 1.3 Reactions of compounds 3 and 4 with *d*_6_-benzene

### Reaction of compound 3 with C_6_D_6_

In a J. Youngs tap NMR tube, C_6_D_6_ (0.6 mL) was added to **3** (20 mg, 0.016 mmol) and monitored by ^1^H NMR spectroscopy at room temperature for ca. 16 hours.

**Supplementary Figure 20:** ^1^H NMR spectrum (500 MHz, C_6_D_6_) resulting from the reaction of compound **3** and C_6_D_6_ for 16h at room temperature. The sample contains predominantly **2*-d­*** and EtC_6_D_5_.


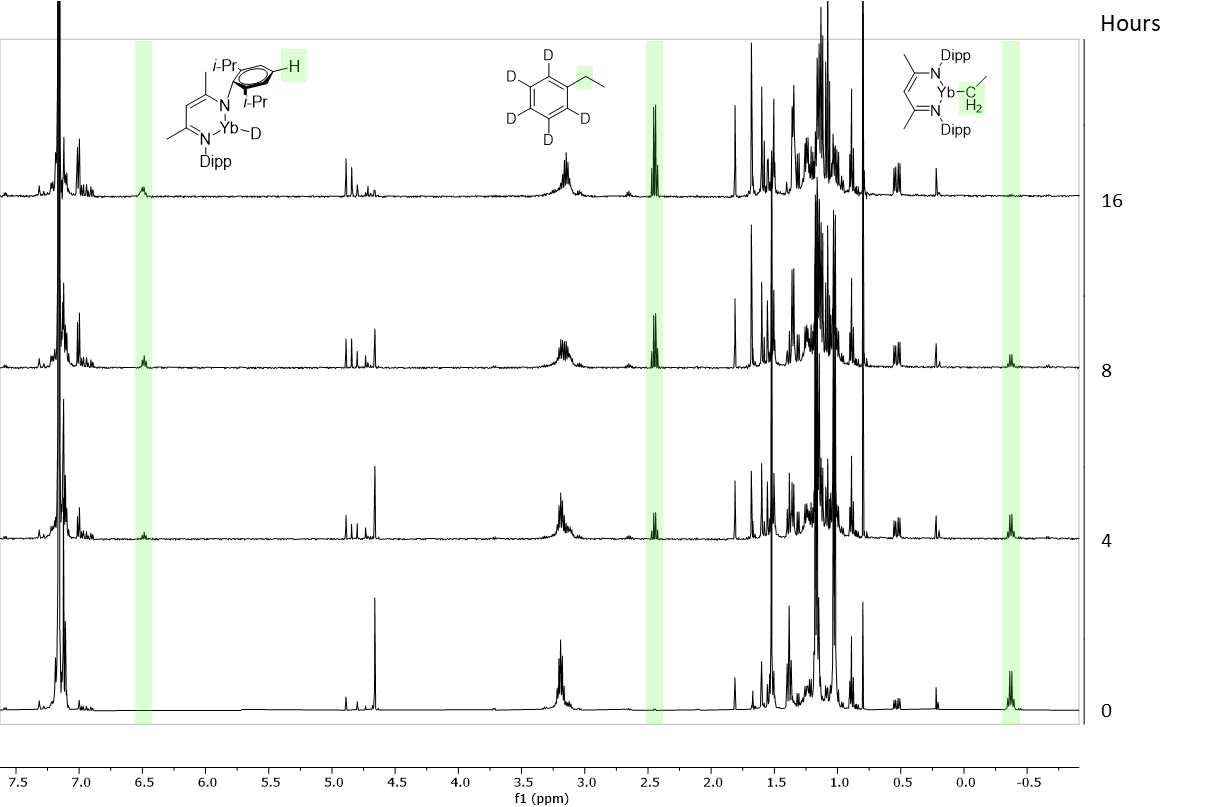


**Supplementary Figure 21:** 1) ^1^H NMR spectrum of predominantly compound **3** in C_6_D_6_; 2) Same sample after ca. 4 hours at room temperature. Production of EtC_6_D_5_ clear by the quartet resonance at 2.45 ppm. The generation of [HC{(Me)CN(2,6-^i^Pr_2_C_6_H_3_)}_2_YbD]_2_ (**2-*d***) is evident from the clear triplet signal centred at 6.51 ppm and the associated methine signal 4.84 ppm. 3) Same sample after ca. 8 hours, new quartet resonance at – 0. 67 ppm assigned to [{HC{(Me)CN(2,6-^i^Pr_2_C_6_H_3_)}_2_Yb}_2_(D)Et] intermediate; 4) Same sample after 16 hours at room temperature contains predominantly **2-*d*** and EtC_6_D_5_.


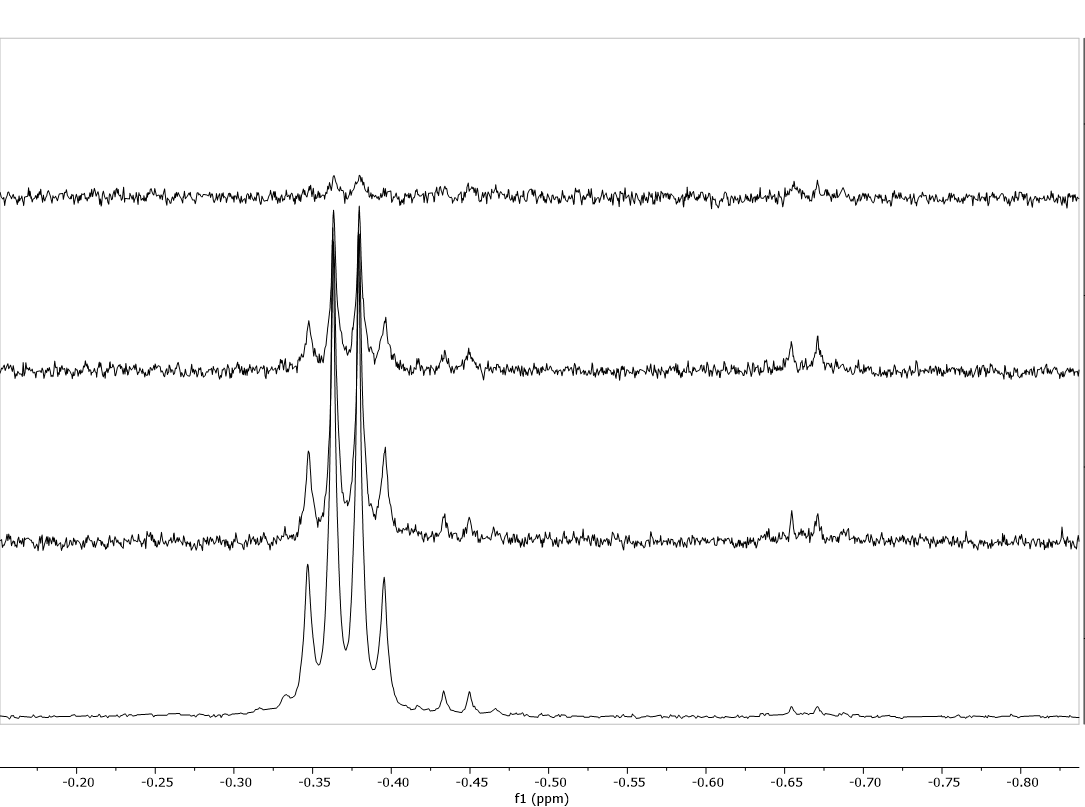


**Figure S22:** Expansion of the upfield region of spectra shown in Supplementary Figure 21, highlighting the disappearance of the α-CH_2_ signal of compound **3** (– 0.36 ppm) and the α-CH_2_ signal of the proposed [{HC{(Me)CN(2,6-^i^Pr_2_C_6_H_3_)}_2_Yb}_2_(D)Et] intermediate at – 0.67 ppm.

### Reaction of compound 4 with C_6_D_6_

In a J. Youngs tap NMR tube, C_6_D_6_ (0.6 mL) was added to **4** (20 mg, 0.016 mmol) and monitored by ^1^H NMR spectroscopy at room temperature for 24 hours.

**Supplementary Figure 23:** ^1^H NMR spectrum (500 MHz, C_6_D_6_) resulting from the reaction of compound **4** and C_6_D_6_ for 24h at room temperature. The sample contains predominantly **2*-d­*** and PrC_6_D_5_.


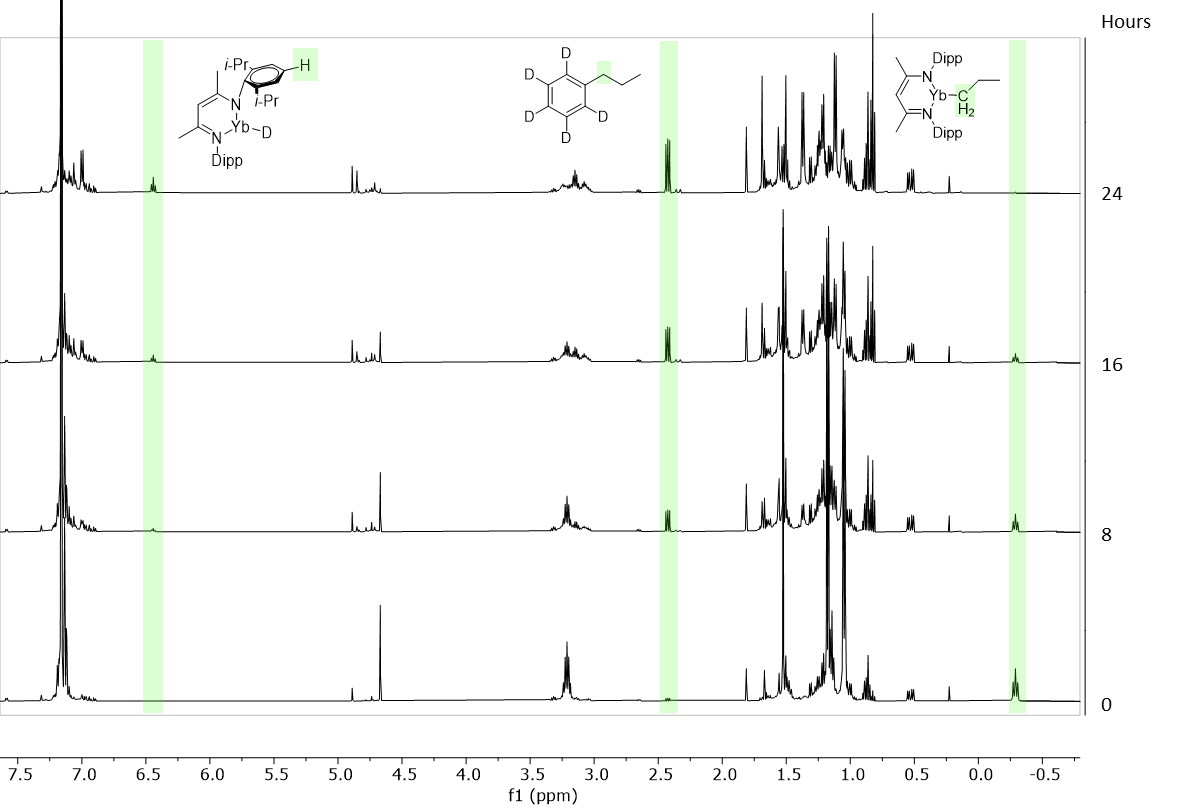


**Supplementary Figure 24:** 1) ^1^H NMR spectrum of predominantly compound **4** in C_6_D_6_; 2) Same sample after ca. 8 hours at room temperature. Production of *n-*PrC_6_D_5_ clear by the triplet resonance at 2.43 ppm; 3) Same sample after ca. 16 hours, the generation of [HC{(Me)CN(2,6-^i^Pr_2_C_6_H_3_)}_2_YbD]_2_ (**2-*d***) is evident from the clear triplet signal centred at 6.44 ppm and the associated methine signal 4.85 ppm. Additionally, a new triplet resonance at – 0.61 ppm is assigned to a [{HC{(Me)CN(2,6-^i^Pr_2_C_6_H_3_)}_2_Yb}_2_(D)Pr] intermediate; 4) Same sample after 24 hours at room temperature contains predominantly **2-*d*** and *n-*PrC_6_D_5_.


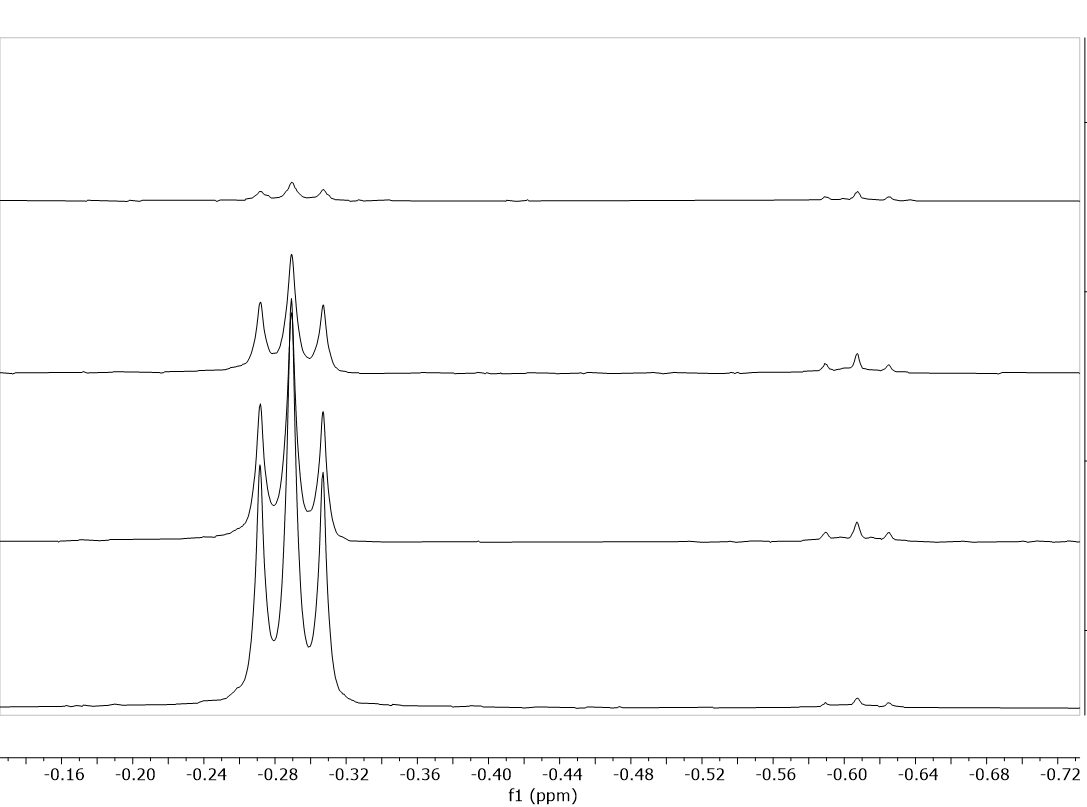


**Figure S25:** Expansion of the upfield region of spectra shown in Supplementary Figure 24, highlighting the disappearance of the α-CH_2_ signal of compound **4** (– 0.29 ppm) and the α-CH_2_ signal of the proposed [{HC{(Me)CN(2,6-^i^Pr_2_C_6_H_3_)}_2_Yb}_2_(D)Pr] intermediate at – 0.61 ppm.

## 1.4 Hydroarylation of ethene or propene with *d*_6_-benzene/benzene

### Hydroarylation of ethene with C_6_D_6_ catalysed by 2

In a J. Youngs tap NMR tube, C_6_D_6_ (0.6 mL) was added to **2** (20 mg, 0.016 mmol), degassed and exposed to 1 atmosphere of ethene. The reaction was monitored by ^1^H NMR spectroscopy at room temperature for ca. 5 days. The volatile *d*_5_-ethylbenzene could be vacuum transferred by trap to trap distillation.


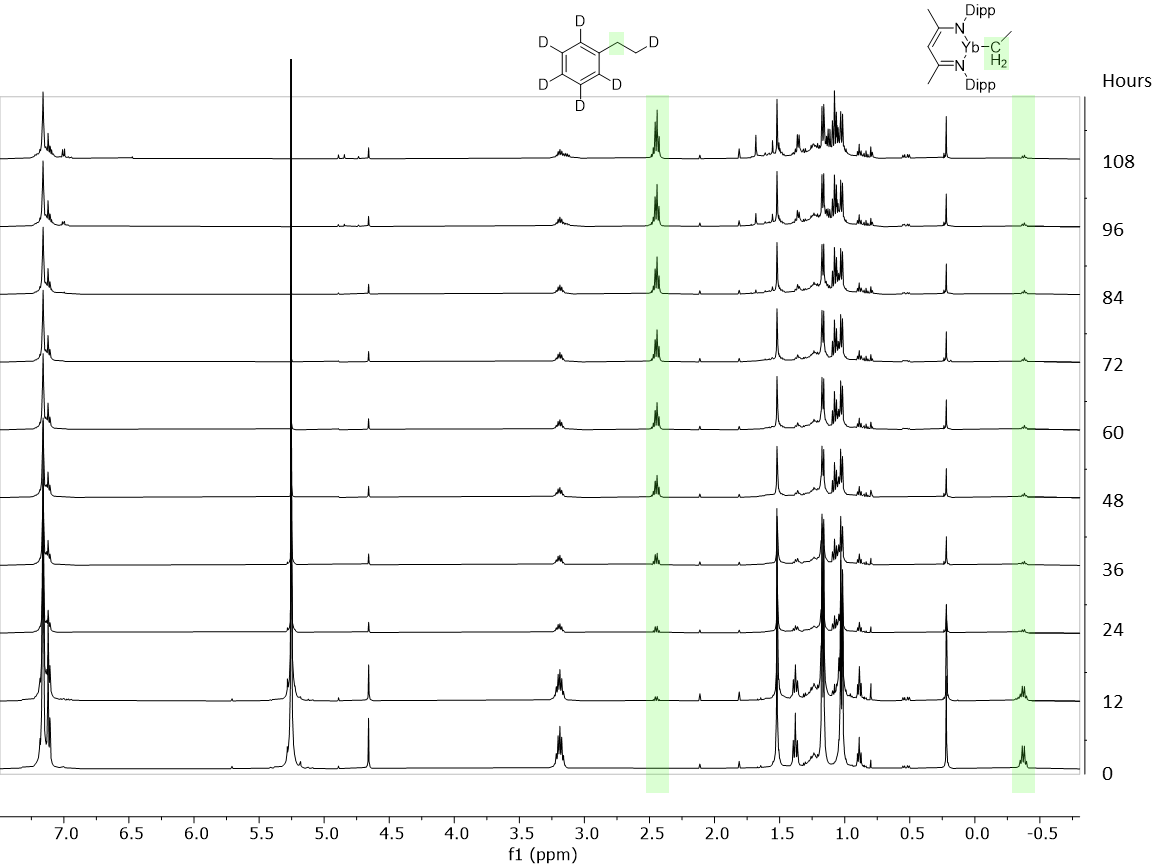


**Supplementary Figure 26:** Stacked ^1^H NMR spectra of a 20 mg sample of compound **2** in 0.6 mL of C_6_D_6_ under 1 atmosphere of ethene at room temperature and monitored for 5 days. Spectra were recorded every 12 hours.

**Supplementary Figure 27:** ^1^H NMR spectrum (500 MHz, C_6_D_6_) of the volatiles isolated by trap to trap distillation from the hydroarylation of ethene with C_6_D_6_ catalysed by **2** at room temperature and monitored for 5 days. Major products observed are *d*_5_-ethylbenzene and *d*_5_-butylbenzene.

**Supplementary Figure 28:** ^13^C{^1^H} NMR spectrum (126 MHz, C_6_D_6_) of the volatiles isolated by trap to trap distillation from the hydroarylation of ethene with C_6_D_6_ catalysed by **2** at room temperature and monitored for 5 days. Major products observed are *d*_5_-ethylbenzene and *d*_5_-butylbenzene.


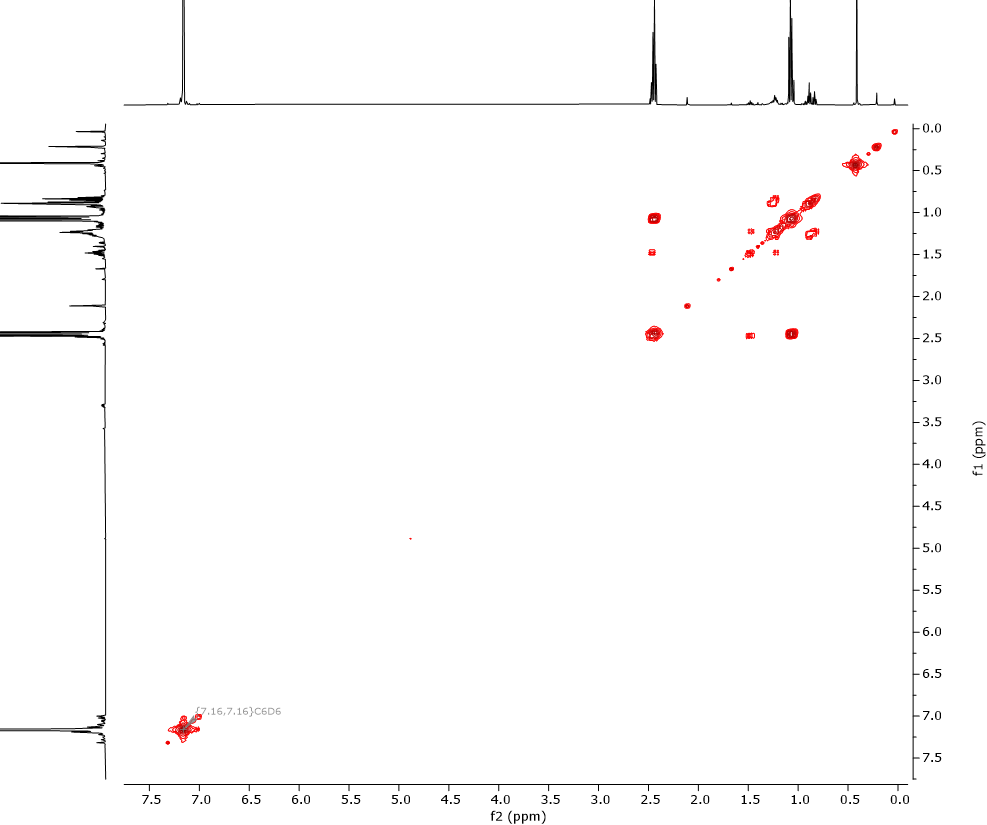


**Supplementary Figure 29:** ^1^H – ^1^H COSY NMR spectrum (500 MHz, C_6_D_6_) of the volatiles isolated by trap to trap distillation from the hydroarylation of ethene with C_6_D_6_ catalysed by **2** at room temperature and monitored for 5 days. Major products observed are *d*_5_-ethylbenzene and *d*_5_-butylbenzene.


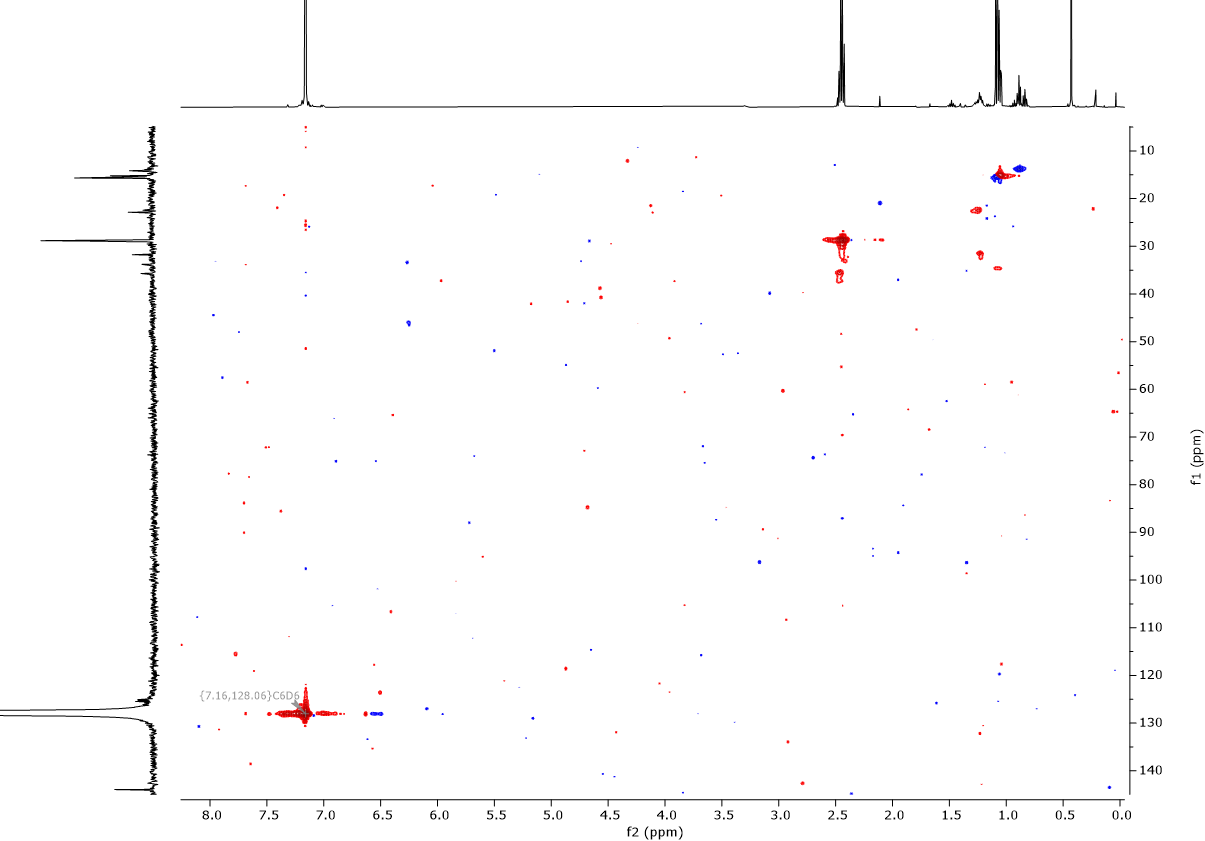


**Supplementary Figure 30:** ^1^H – ^13^C HSQC NMR spectrum (500 MHz, C_6_D_6_) of the volatiles isolated by trap to trap distillation from the hydroarylation of ethene with C_6_D_6_ catalysed by **2** at room temperature and monitored for 5 days. Major products observed are *d*_5_-ethylbenzene and *d*_5_-butylbenzene.


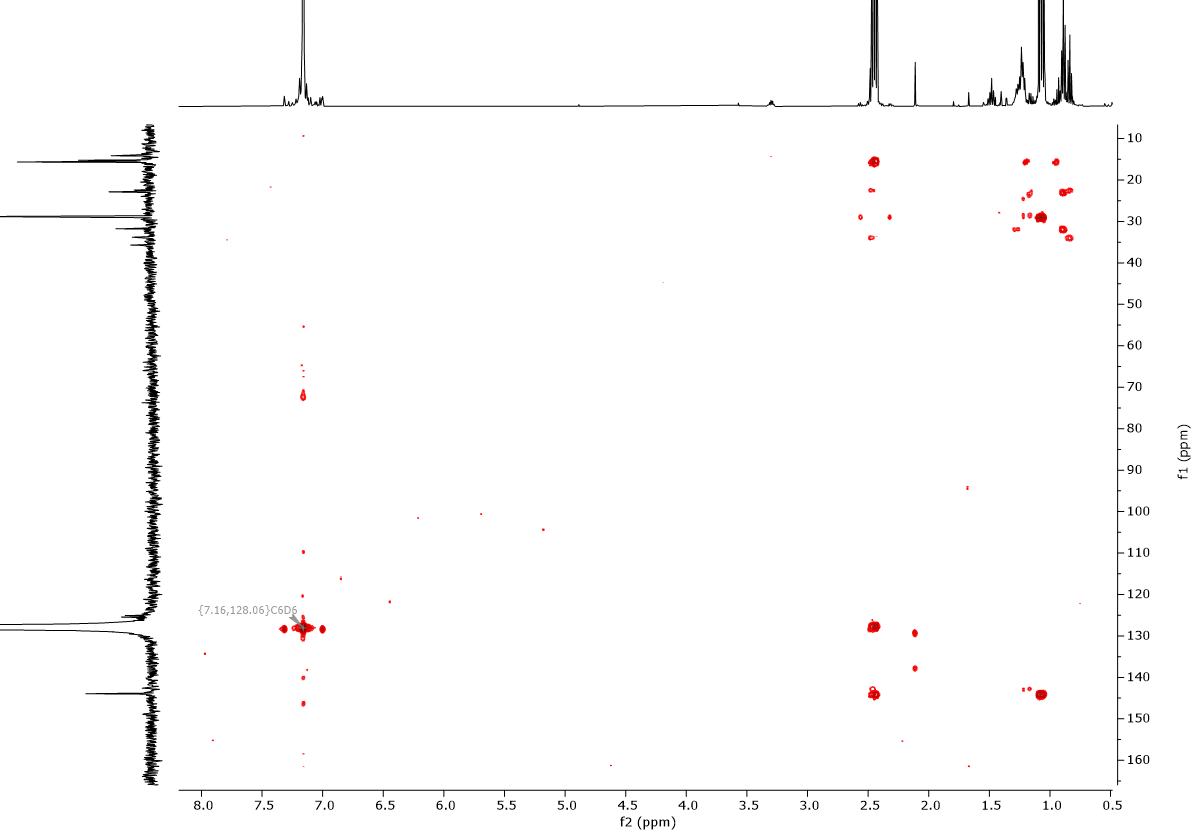


**Supplementary Figure 31:** ^1^H – ^13^C HMBC NMR spectrum (500 MHz, C_6_D_6_) of the volatiles isolated by trap to trap distillation from the hydroarylation of ethene with C_6_D_6_ catalysed by **2** at room temperature and monitored for 5 days. Major products observed are *d*_5_-ethylbenzene and *d*_5_-butylbenzene.


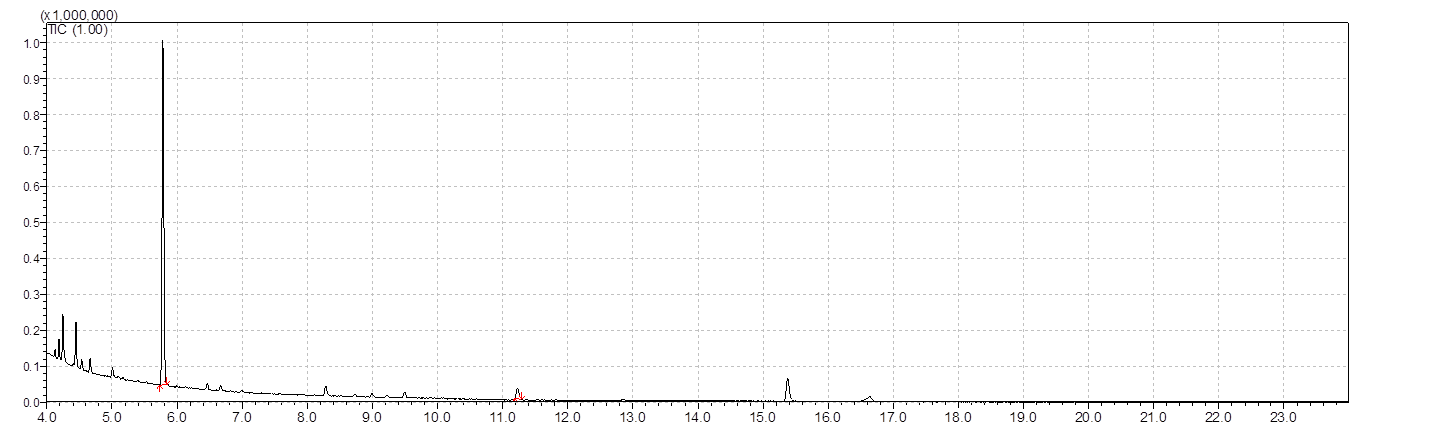


**Supplementary Figure 32:** GC-MS chromatogram of the volatiles isolated by trap to trap distillation from the hydroarylation of ethene with C_6_D_6_ catalysed by **2** at room temperature and monitored for 5 days, showing the peaks of *d*_5_-ethylbenzene (5.8 min) and *d*_5_-butylbenzene (11.2 min).


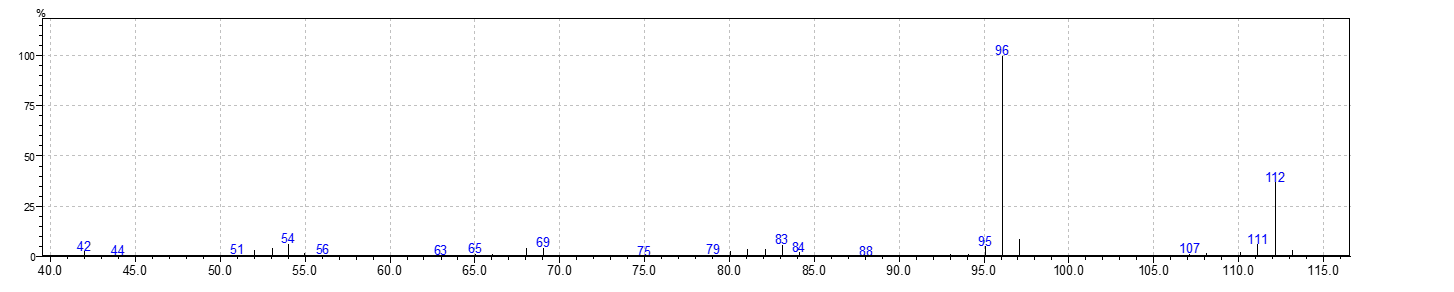


**Supplementary Figure 33:** GC-MS trace showing the molecular ion of *d*_5_-ethylbenzene (5.8 min).


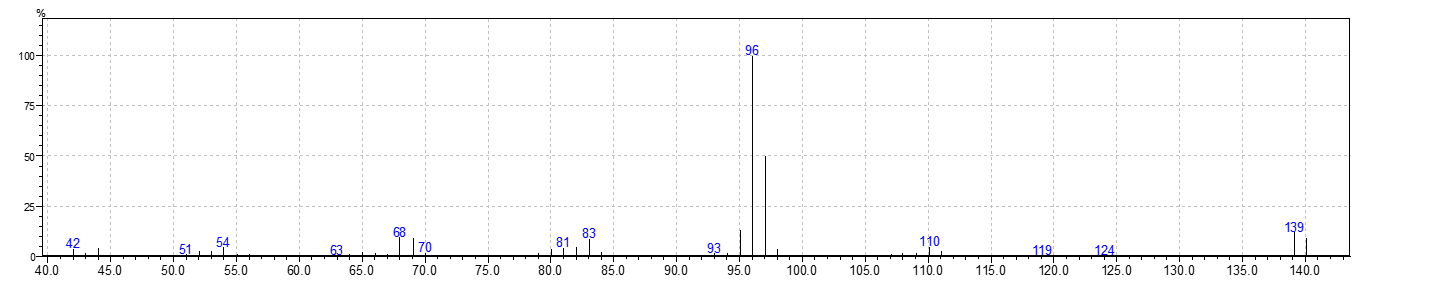


**Supplementary Figure 34:** GC-MS trace showing the molecular ion of *d*_5_-butylbenzene (11.2 min).


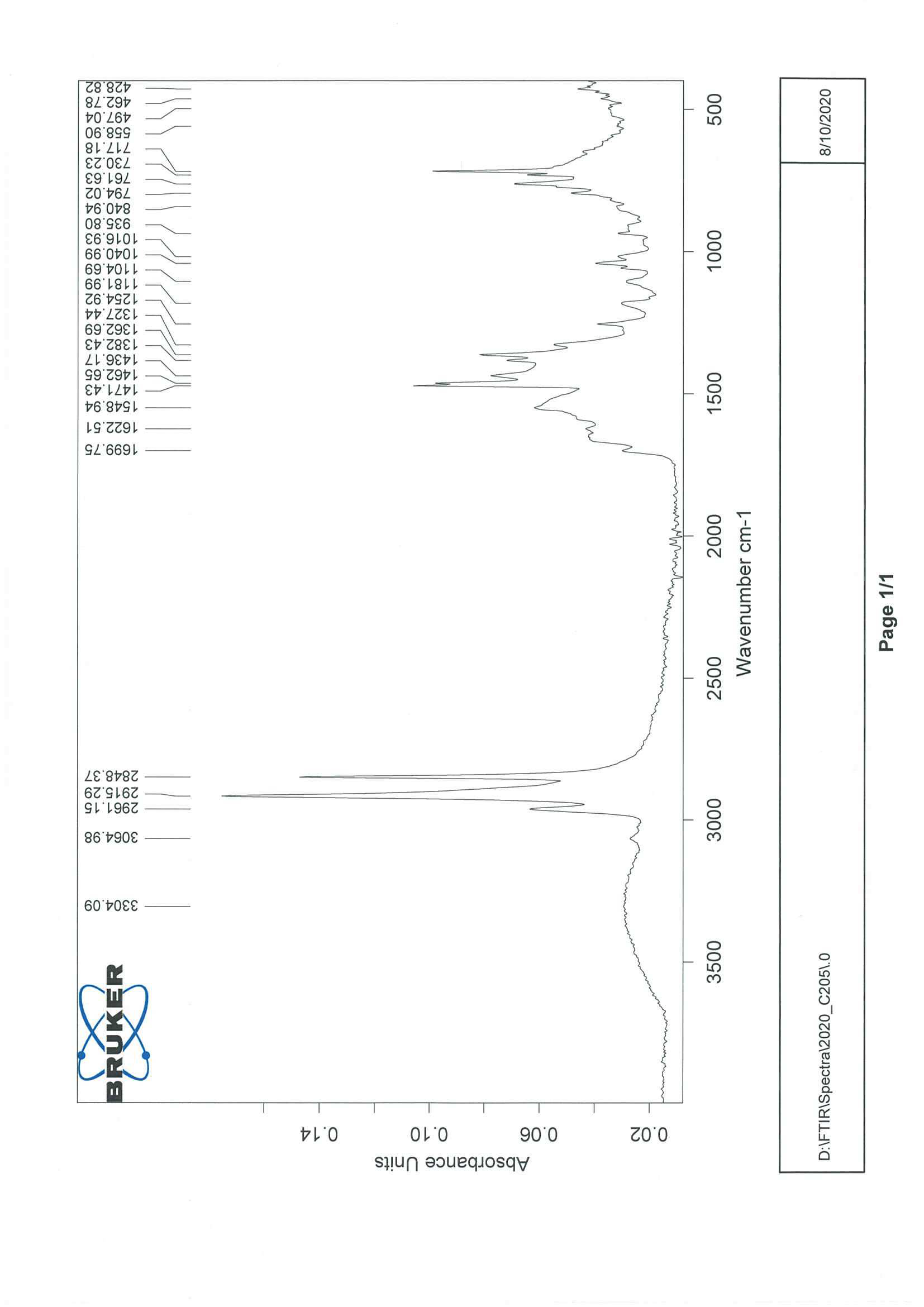


**Supplementary Figure 35:** Polyethene characterisation by FTIR.^3-7^

### Hydroarylation of ethene with C_6_D_6_ catalysed by 2 at 40 ^o^C

In a J. Youngs tap NMR tube, C_6_D_6_ (0.6 mL) was added to **2** (20 mg, 0.016 mmol), degassed and exposed to 1 atmosphere of ethene. The reaction was monitored by ^1^H NMR spectroscopy at 40 ^o^C for ca. 44 hours. The volatile *d*_5_-ethylbenzene could be vacuum transferred by trap to trap distillation.


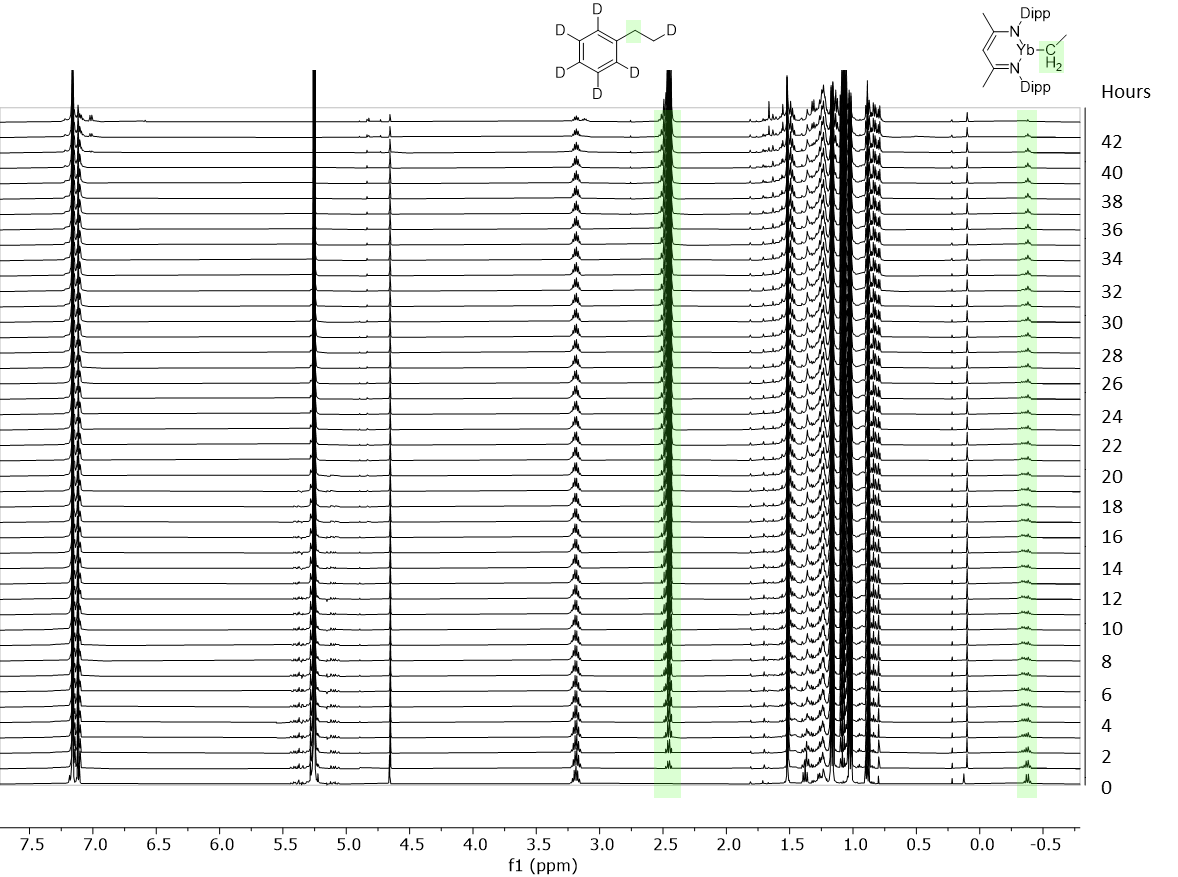


**Supplementary Figure 36:** Stacked ^1^H NMR spectra of a 20 mg sample of compound **2** in 0.6 mL of C_6_D_6_ under 1 atmosphere of ethene, heated to 40 ^o^C and monitored for ca. 44 hours. Spectra were recorded every hour.

**Supplementary Figure 37:** ^1^H NMR spectrum (500 MHz, C_6_D_6_) of the volatiles isolated by trap to trap distillation from the hydroarylation of ethene with C_6_D_6_ catalysed by **2** at 40 ^o^C and monitored for 44 hours. Major products observed are *d*_5_-ethylbenzene and *d*_5_-butylbenzene.

**Supplementary Figure 38:** ^13^C{^1^H} NMR spectrum (126 MHz, C_6_D_6_) of the volatiles isolated by trap to trap distillation from the hydroarylation of ethene with C_6_D_6_ catalysed by **2** at 40 ^o^C and monitored for 44 hours. Major products observed are *d*_5_-ethylbenzene and *d*_5_-butylbenzene.

**Supplementary Figure 39:** ^1^H – ^1^H COSY NMR spectrum (500 MHz, C_6_D_6_) of the volatiles isolated by trap to trap distillation from the hydroarylation of ethene with C_6_D_6_ catalysed by **2** at 40 ^o^C and monitored for 44 hours. Major products observed are *d*_5_-ethylbenzene and *d*_5_-butylbenzene.

**Supplementary Figure 40:** ^1^H – ^13^C HSQC NMR spectrum (500 MHz, C_6_D_6_) of the volatiles isolated by trap to trap distillation from the hydroarylation of ethene with C_6_D_6_ catalysed by **2** at 40 ^o^C and monitored for 44 hours. Major products observed are *d*_5_-ethylbenzene and *d*_5_-butylbenzene.

**Supplementary Figure 41:** ^1^H – ^13^C HMBC NMR spectrum (500 MHz, C_6_D_6_) of the volatiles isolated by trap to trap distillation from the hydroarylation of ethene with C_6_D_6_ catalysed by **2** at 40 ^o^C and monitored for 44 hours. Major products observed are *d*_5_-ethylbenzene and *d*_5_-butylbenzene.

**Supplementary Figure 42:** ^2^H NMR spectrum (77 MHz, C_6_D_6_) of the volatiles isolated by trap to trap distillation from the hydroarylation of ethene with C_6_D_6_ catalysed by **2** at 40 ^o^C and monitored for 44 hours. Major products observed are *d*_5_-ethylbenzene and *d*_5_-butylbenzene.


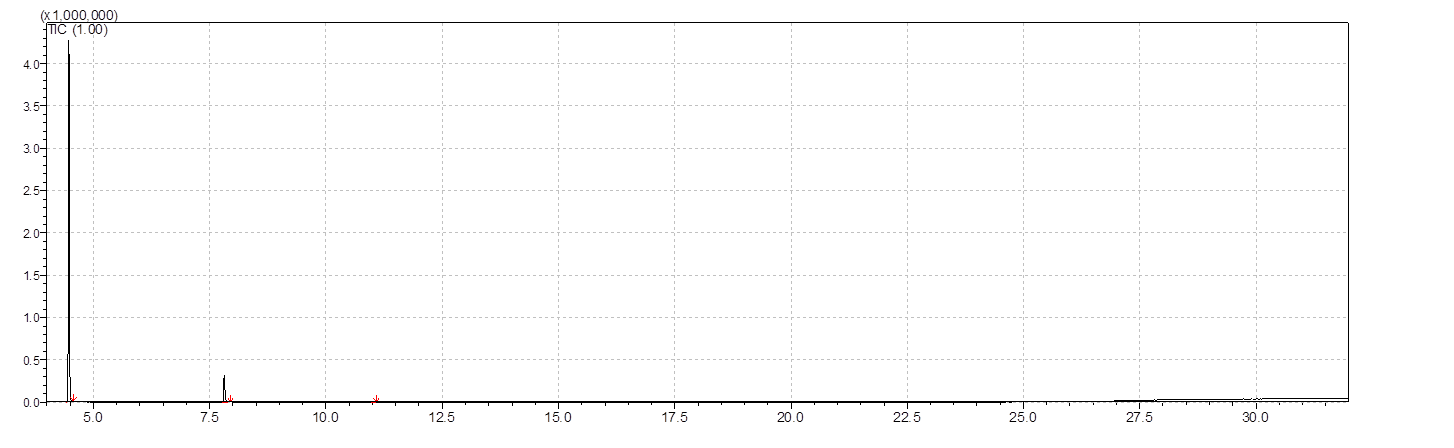


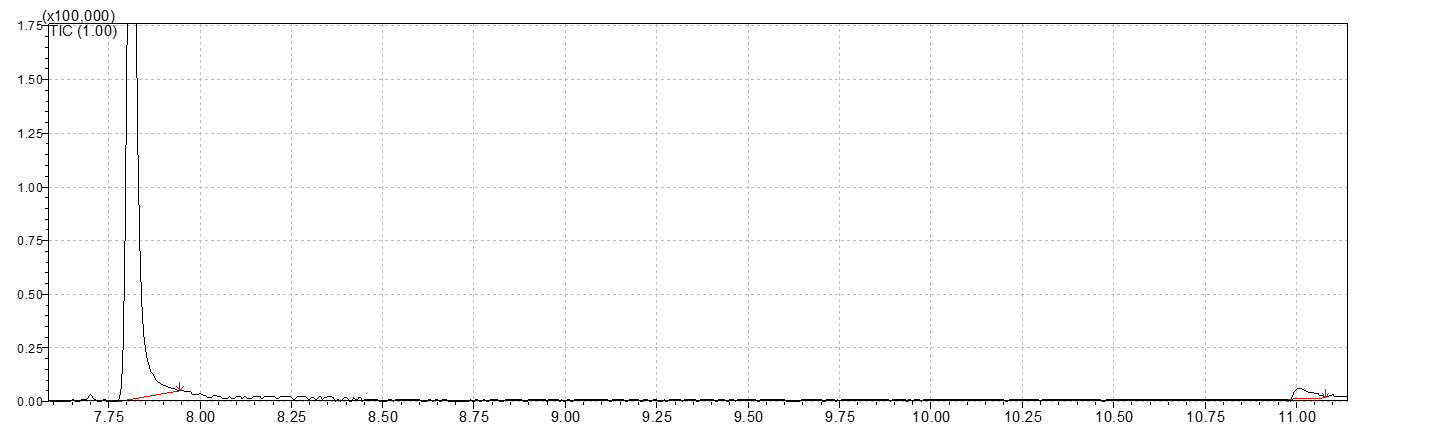


**Figure S43:** GC-MS chromatogram of the volatiles isolated by trap to trap distillation from the hydroarylation of ethene with C_6_D_6_ catalysed by **2** at 40 ^o^C and monitored for 44 hours, showing the peaks of *d*_5_-ethylbenzene (4.5 min), *d*_5_-butylbenzene (7.8 min) and *d*_5_-hexylbenzene (11.1 min).


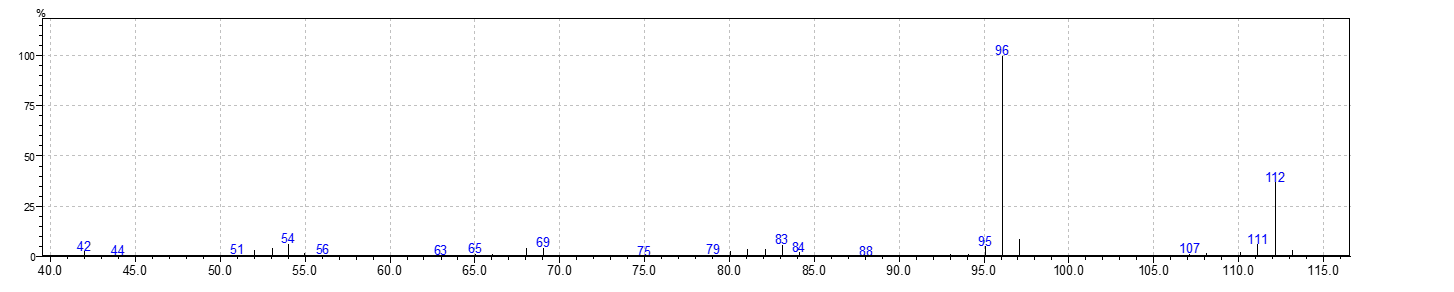


**Supplementary Figure 44:** GC-MS trace showing the molecular ion of *d*_5_-ethylbenzene (4.5 min).


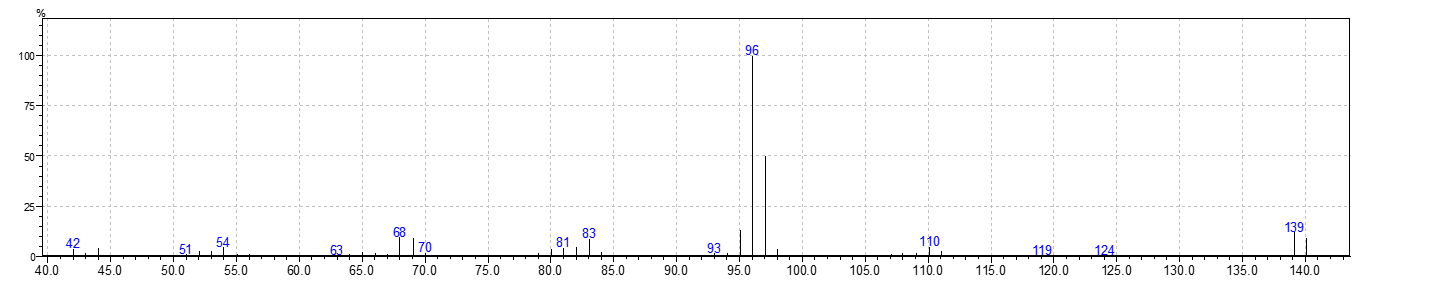


**Supplementary Figure 45:** GC-MS trace showing the molecular ion of *d*_5_-butylbenzene (7.8 min).


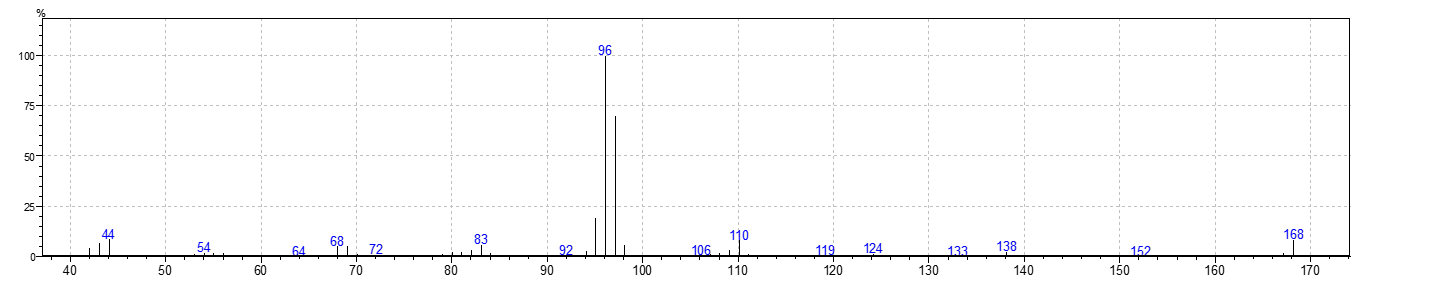


**Supplementary Figure 46:** GC-MS trace showing the molecular ion of *d*_5_-hexylbenzene (11.1 min).

### Hydroarylation of ethene with C_6_H_6_ catalysed by 2

In a J. Youngs tap NMR tube, C_6_H_6_ (0.6 mL) was added to **2** (20 mg, 0.016 mmol), degassed and exposed to 1 atmosphere of ethene. The reaction was monitored by ^1^H NMR spectroscopy at 40 ^o^C for 24 hours. The volatile *d*_5_-ethylbenzene could be vacuum transferred by trap to trap distillation.


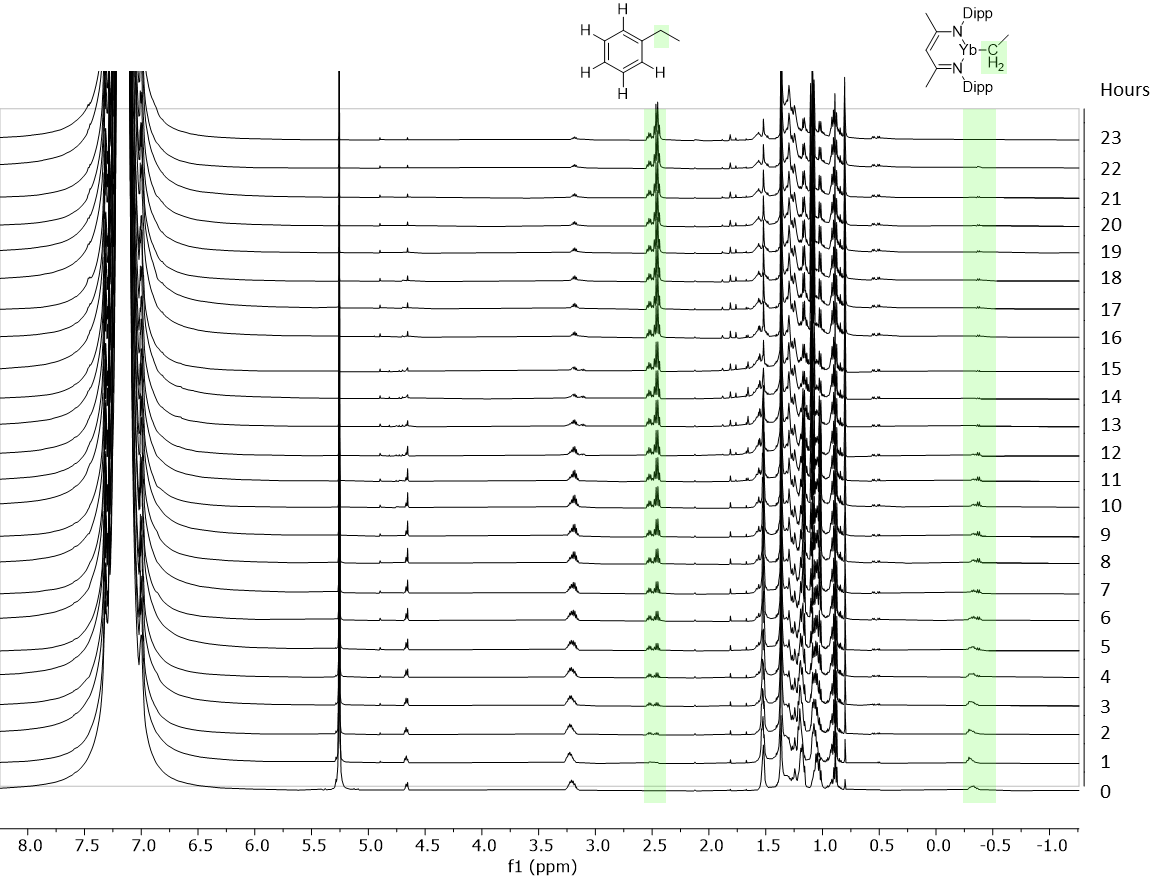


**Supplementary Figure 47:** Stacked ^1^H NMR spectra of a 20 mg sample of compound **2** in 0.6 mL of C_6_H_6_ under 1 atmosphere of ethene, heated to 40 ^o^C and monitored for ca. 24 hours. Spectra were recorded every hour.

### Hydroarylation of propene with C_6_D_6_ catalysed by 2

In a J. Youngs tap NMR tube, C_6_D_6_ (0.6 mL) was added to **2** (20 mg, 0.016 mmol), degassed and exposed to 1 atmosphere of propene. The reaction was monitored by ^1^H NMR spectroscopy at room temperature for ca. 8 days. The volatile *d*_5_-propylbenzene could be vacuum transferred by trap to trap distillation.


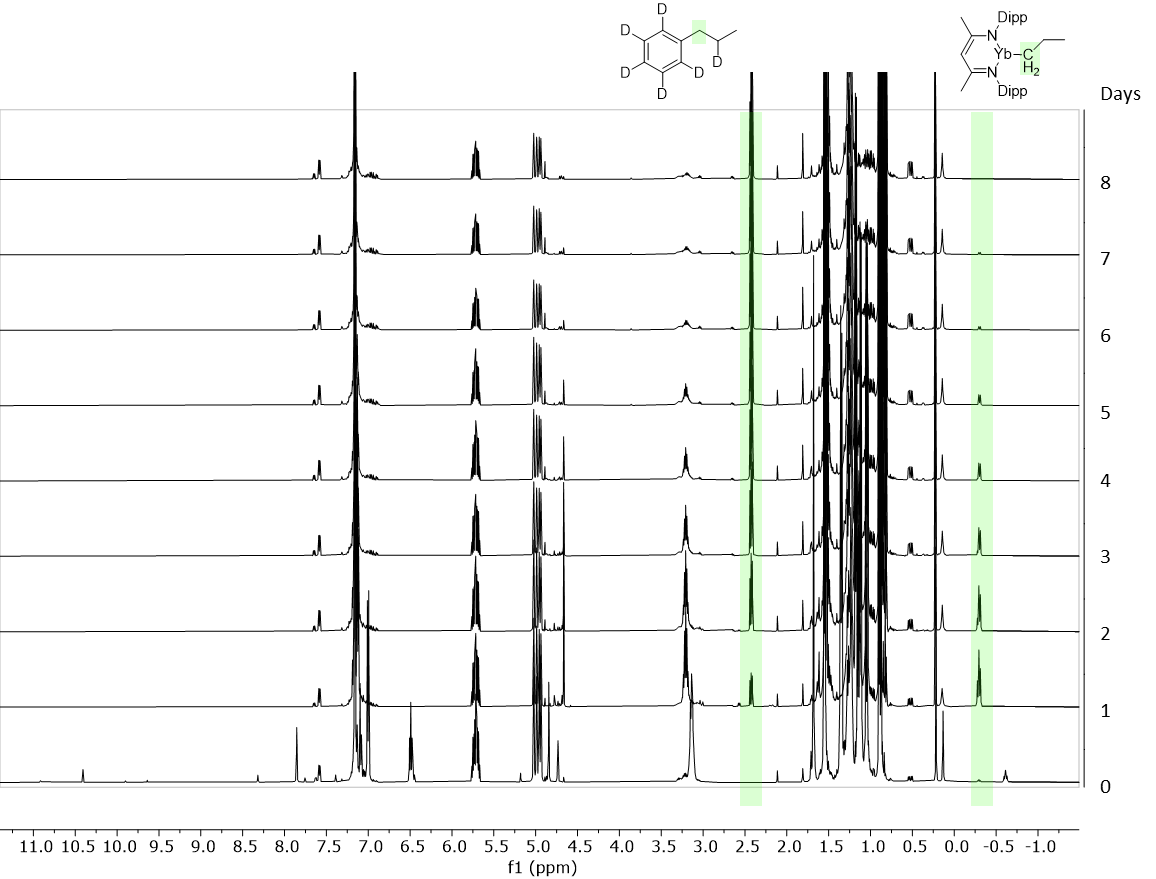


**Supplementary Figure 48:** Stacked ^1^H NMR spectra of a 20 mg sample of compound **2** in 0.6 mL C_6_D_6_ under 1 atmosphere of propene at room temperature and monitored for 8 days. Spectra were recorded every 24 hours.

**Figure S49:** Expansion of the up and down field region of first spectra shown in Supplementary Figure 48, highlighting the new Yb-H signal (10.41ppm, triplet, *J*_Yb-H_ = 251 Hz) which occurs in a 1:2 ratio with the α-CH_2_ signal of the proposed [{HC{(Me)CN(2,6-^i^Pr_2_C_6_H_3_)}_2_Yb}_2_(D)Pr] intermediate at (– 0.61 ppm).

**Supplementary Figure 50:** ^1^H NMR spectrum (500 MHz, C_6_D_6_) of the volatiles isolated by trap to trap distillation from the hydroarylation of propene with C_6_D_6_ catalysed by **2** at room temperature and monitored for 8 days. Major product is *d*_5_-propylbenzene (toluene (Tol), hexane (Hex) and grease also present).

**Supplementary Figure 51:** ^13^C{^1^H} NMR spectrum (126 MHz, C_6_D_6_) of the volatiles isolated by trap to trap distillation from the hydroarylation of propene with C_6_D_6_ catalysed by **2** at room temperature and monitored for 8 days. Major product is *d*_5_-propylbenzene (toluene (Tol), hexane (Hex) and grease also present).

**Supplementary Figure 52:** ^1^H – ^1^H COSY NMR spectrum (500 MHz, C_6_D_6_) of the volatiles isolated by trap to trap distillation from the hydroarylation of propene with C_6_D_6_ catalysed by **2** at room temperature and monitored for 8 days. Major product is *d*_5_-propylbenzene (toluene (Tol), hexane (Hex) and grease also present).

**Supplementary Figure 53:** ^1^H – ^13^C HSQC NMR spectrum (500 MHz, C_6_D_6_) of the volatiles isolated by trap to trap distillation from the hydroarylation of propene with C_6_D_6_ catalysed by **2** at room temperature and monitored for 8 days. Major product is *d*_5_-propylbenzene (toluene (Tol), hexane (Hex) and grease also present).

**Supplementary Figure 54:** ^1^H – ^13^C HMBC NMR spectrum (500 MHz, C_6_D_6_) of the volatiles isolated by trap to trap distillation from the hydroarylation of propene with C_6_D_6_ catalysed by **2** at room temperature and monitored for 8 days. Major product is *d*_5_-propylbenzene (toluene (Tol), hexane (Hex) and grease also present).

**Supplementary Figure 55:** ^2^H NMR spectrum (77 MHz, C_6_D_6_) of the volatiles isolated by trap to trap distillation from the hydroarylation of propene with C_6_D_6_ catalysed by **2** at room temperature and monitored for 8 days. Major product is *d*_5_-propylbenzene (toluene (Tol), hexane (Hex) and grease also present).


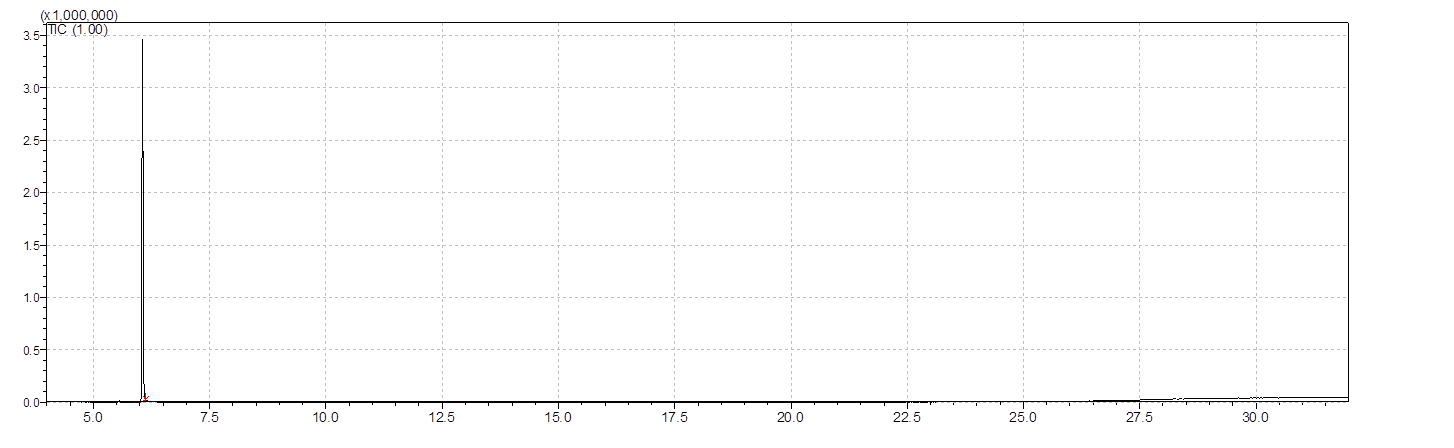


**Supplementary Figure 56:** GC-MS chromatogram of the volatiles isolated by trap to trap distillation from the hydroarylation of propene with C_6_D_6_ catalysed by **2** at room temperature and monitored for 8 days, showing the peak of *d*_5_-propylbenzene (6.2 min).

**
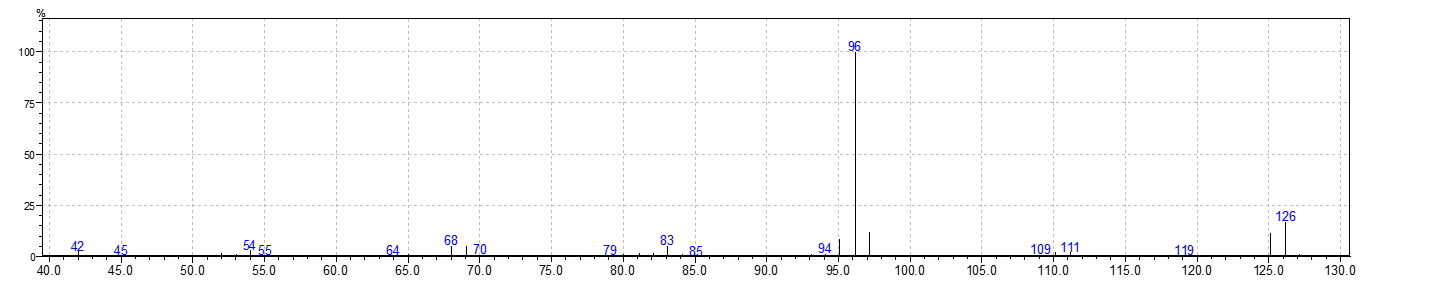
**

**Supplementary Figure 57:** GC-MS trace showing the molecular ion of *d*_5_-propylbenzene (6.2 min).

### Hydroarylation of propene with C_6_H_6_ catalysed by 2

In a J. Youngs tap NMR tube, C_6_H_6_ (0.6 mL) was added to **2** (20 mg, 0.016 mmol), degassed and exposed to 1 atmosphere of propene. The reaction was monitored by ^1^H NMR spectroscopy at 40 ^o^C for ca. 20 hours. The volatile *d*_5_-propylbenzene could be vacuum transferred by trap to trap distillation.


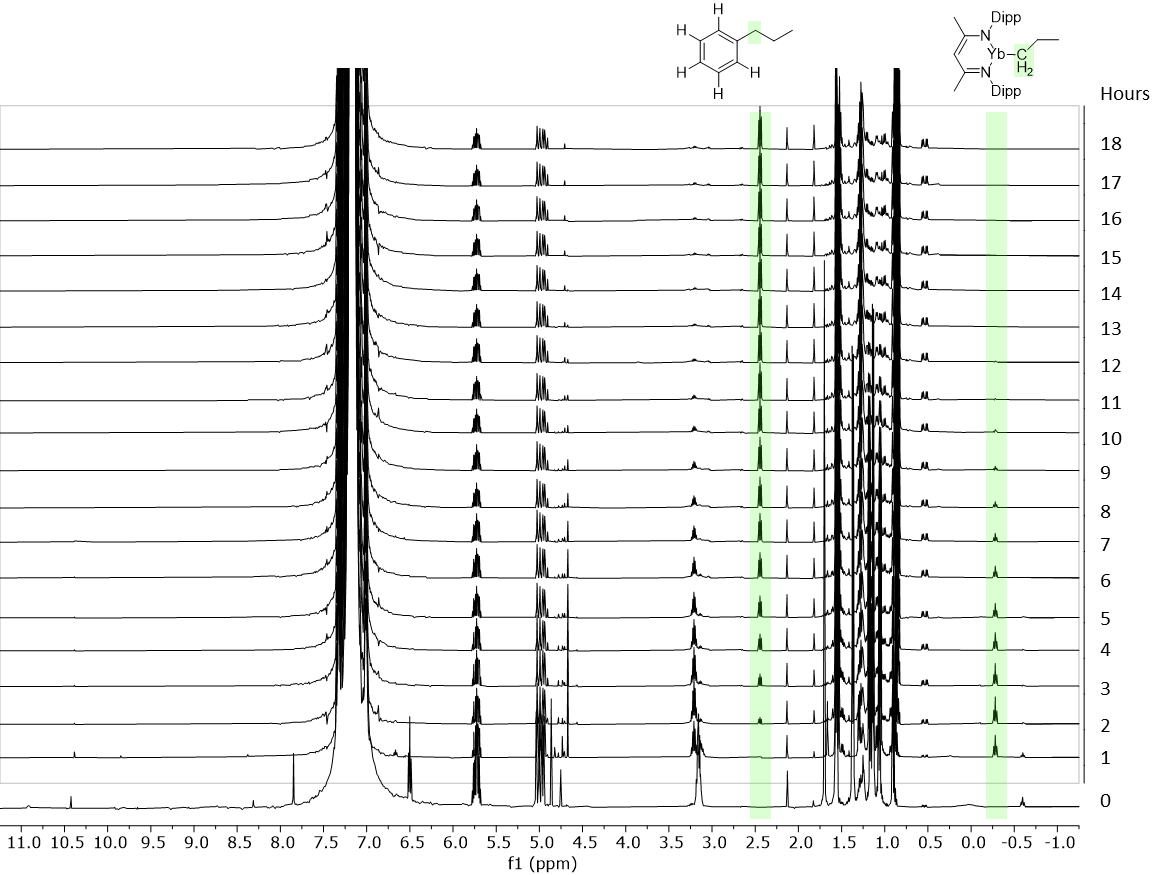


**Supplementary Figure 58:** Stacked ^1^H NMR spectra of a 20 mg sample of compound **2** in 0.6 mL of C_6_H_6_ under 1 atmosphere of propene heated to 40 ^o^C and monitored for ca. 20 hours. Spectra were recorded every hour. First spectrum shows the initial presence of the mixed ytterbium alkyl-hydride complex, **5**, by the appearance of a triplet centred at -0.61 ppm

### Hydroarylation of propene with C_6_H_6_ catalysed by 2

In a J. Youngs tap NMR tube, C_6_H_6_ (0.6 mL) was added to **2** (20 mg, 0.016 mmol), degassed and exposed to 1 atmosphere of propene. The reaction was monitored by ^1^H NMR spectroscopy at room temperature for ca. 64 hours. The volatile *d*_5_-propylbenzene could be vacuum transferred by trap to trap distillation.


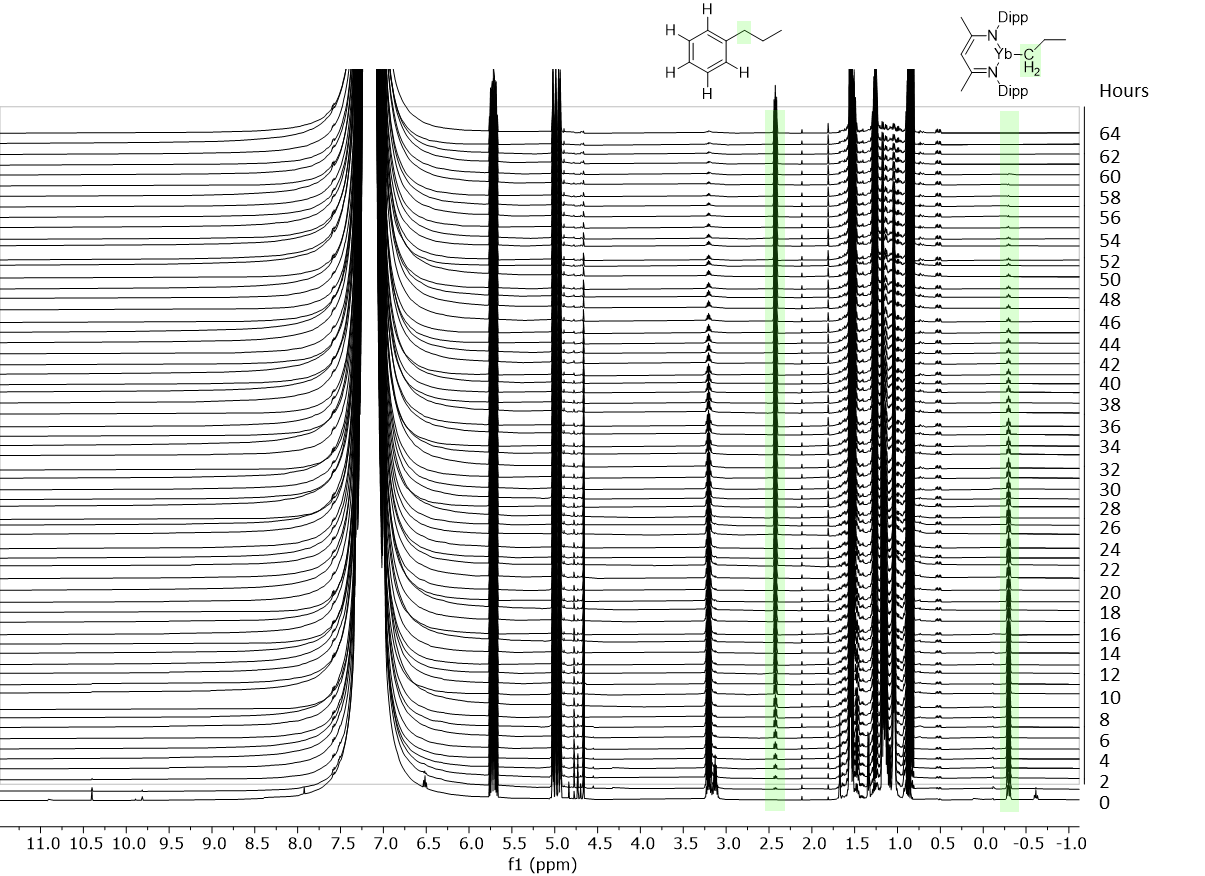


**Supplementary Figure 59:** Stacked ^1^H NMR spectra of a 20 mg sample of compound **2** in 0.6 mL of C_6_H_6_ under 1 atmosphere of propene at room temperature and monitored for ca. 64 hours. Spectra were recorded every hour.

## 1.5 Crystallography

Compounds [Yb{N(SiMe_3_)_2_}_2_]_2_, **1**, **2**, **3**, **4** and **6** were collected on an Agilent SuperNova diffractometer fitted with an EOS S2 detector. All datasets were collected using CuKα radiation (λ = 1.54184 Å). The crystal structure of compound **1** had unresolvable solvent electron density, which was accounted for using SQUEEZE^8^ and produced infinite void channels along the *c*-axis. Crystals of **3** were non-merohedrally twinned; the resulting crystal structure was solved using the major component and finally refined using both twin components (HKLF5 refinement). The structure of **6** contained a disordered propene cation (54:46 disorder), with both parts being restrained to be like the non-disordered propene cation (SAME restraint). This structure also contains half a molecule of benzene per asymmetric unit (the other half generated by symmetry) and was also disordered. The disordered propene cation and benzene atoms needed to be refined isotropically for stable refinement. Crystal data and structure refinement tables can be found in the supplementary information. CCDC 2032027-2032031 and 2034334 contains the supplementary crystallographic data for this paper. These data can be obtained free of charge from The Cambridge Crystallographic Data Centre via [www.ccdc.cam.ac.uk/structures](http://www.ccdc.cam.ac.uk/structures).

All carbon-bound hydrogen atoms were placed at calculated positions and refined using a standard riding model. The treatment of the remaining hydrogen atoms is mentioned per structure below. All non-hydrogen atoms were refined anisotropically, unless specified below.

**[Yb{N(SiMe_3_)_2_}_2_]_2_**

No extra refinement details. No solvent molecules or disorder is present in the structure. The entire Yb_2_L_4_ unit is present in the asymmetric unit.

### [HC{(Me)CN(2,6-^i^Pr_2_C_6_H_3_)}_2_YbN(SiMe_3_)_2_]_4_ (1)

This structure contains infinite solvent channels along the c-axis, bearing highly disordered solvent molecules that could not be reasonably refined. Consequently, SQUEEZE was used to account for the unmodelled electron density in these voids, yielding 320 electrons per 1435 A3 void. This is reasonably consistent with 6 molecules of toluene or hexane (both present in the recrystallisation conditions). The entire LYbL unit is present in the asymmetric unit but forms a pseudo-tetrameric structure via interactions with the Yb atom and a methyl group (C35) from a neighbouring molecule.

### [HC{(Me)CN(2,6-^i^Pr_2_C_6_H_3_)}_2_YbH]_2_ (2)

The hydrogen atom H1 is the hydride atoms that is part of the central Yb_2_H_2_ fragment. The Yb--H bond length is consistent with other reported ytterbium hydride dimer structures (e.g. CCDC 112689). The positions and isotropic displacement parameters for this hydride atom are freely refined. No solvent molecules or disorder is present in the structure. Half of the (LYbH)_2_ dimer is present in the asymmetric unit, with the other generated by symmetry (inversion).

### [HC{(Me)CN(2,6-^i^Pr_2_C_6_H_3_)}_2_Yb(C_2_H_5_)]_2_ (3)

Crystals of this molecule suffered from non-merohedral twinning with two major overlapping lattices observed. The major component was used for structure solution, and both components were used to complete refinement using HKLF5 refinement with BASF refining to 0.44. There was one half of the Yb_2_L_2_ dimer and one molecule of toluene (present in recrystallisation mixture) in the asymmetric unit. This toluene molecule was only partial (30%) occupancy, which necessitated restraining the carbon atoms to have the same Uij components using SIMU.

**[HC{(Me)CN(2,6-^i^Pr_2_C_6_H_3_)}_2_Yb(C_3_H_7_)]_2_ (4)**

There were two half dimers present in the asymmetric unit, with the other halves generated by symmetry (inversion). Both unique ytterbium atoms were slightly disordered (ca. 90:10 occupancy). Anisotropic refinement alone was insufficient to describe this observation. No other parts of the molecule are obviously disordered - this may be difficult to observe for the light atoms at this occupancy.

**[HC{(Me)CN(2,6-^i^Pr_2_C_6_H_3_)}_2_Yb(CH_2_C(H)CH_2_)]_4_ (6)**

There is half of the tetramer and half of a benzene molecule present in the asymmetric unit, with the other halves generated by symmetry (inversion). The benzene molecule and one of the two bridging propene anions is disordered over two positions (both ca. 53:47 occupancy). These disordered components were not stable to anisotropic refinement, so were refined isotropically.

#### **Supplementary Table 1**. Crystal data and structure refinement for [Yb{N(SiMe_3_)_2_}_2_]_2_, **1** and **2**.

| Compound ID | [Yb{N(SiMe_3_)_2_}_2_]_2_ | **1** | **2** |
| --- | --- | --- | --- |
| CCDC code | 2032027 | 2032031 | 2032028 |
| Empirical formula | C_24_H_72_N_4_Si_8_Yb_2_ | C_35_H_59_N_3_Si_2_Yb ·solvent | C_58_H_84_N_4_Yb_2_ |
| Formula weight (g/mol) | 987.65 | 751.07 | 1183.37 |
| Temperature (K) | 150(2) | 120(2) | 120(2) |
| Crystal system | Triclinic | Tetragonal | Orthorhombic |
| Space group | *P-*1 | *P*4_2_/n | *P*bca |
| Unit cell dimensions |  |  |  |
| a (Å) | 8.86063(17) | 27.2642(4) | 15.87460(10) |
| b (Å) | 12.5461(2) | 27.2642(4) | 16.62170(10) |
| c (Å) | 21.9201(4) | 11.4908(3) | 20.5391(2) |
| α (°) | 73.7431(17) | 90 | 90 |
| β (°) | 86.6377(15) | 90 | 90 |
| γ (°) | 71.2755(17) | 90 | 90 |
| Volume (Å^3^) | 2214.23(8) | 8541.5(3) | 5419.50(7) |
| Z | 2 | 8 | 4 |
| Calculated density (Mg/m^3^) | 1.481 | 1.168 | 1.450 |
| Absorption coefficient (mm^-1^) | 9.814 | 4.743 | 6.492 |
| F(000) | 992 | 3104 | 2400 |
| Crystal size (mm^3^) | 0.145 × 0.111 × 0.092 | 0.120 × 0.056 × 0.037 | 0.151 × 0.131 × 0.072 |
| Theta range for data collection | 3.861 to 73.556° | 4.175 to 73.575° | 4.305 to 73.497° |
| Index ranges | -11 ≤ h ≤ 11  -15 ≤ k ≤ 10  -27 ≤ l ≤ 27 | -32 ≤ h ≤ 33  -33 ≤ k ≤ 31  -13 ≤ l ≤ 14 | -19 ≤ h ≤ 19  -20 ≤ k ≤ 18  -25 ≤ l ≤ 23 |
| Reflections collected | 27313 | 30537 | 21881 |
| Independent reflections | 8866 R(int) = 0.0234 | 8526 R(int) = 0.0436 | 5435 R(int) = 0.0237 |
| Completeness to theta | 100.0% | 99.8% | 100.0% |
| Data / restraints / parameters | 8866 / 0 / 367 | 8526 / 0 / 386 | 5435 / 0 / 303 |
| Goodness-of-fit on F2 | 1.079 | 1.018 | 1.065 |
| Final R indices [I>2sigma(I)] | R1 = 0.0230  wR2 = 0.0556 | R1 = 0.0402  wR2 = 0.0973 | R1 = 0.0213  wR2 = 0.0512 |
| R indices (all data) | R1 = 0.0241  wR2 = 0.0563 | R1 = 0.0544  wR2 = 0.1053 | R1 = 0.0234  wR2 = 0.0524 |
| Largest diff. peak  and hole (e.Å^-3^) | 0.780 and ‑1.378 | 1.306 and ‑1.584 | 0.335 and ‑0.719 |

#### **Supplementary Table 2.** Crystal data and structure refinement for **3**, **4** and **6**.

| Compound ID | **3** | **4** | **6** |
| --- | --- | --- | --- |
| CCDC code | 2032030 | 2032029 | 2034334 |
| Empirical formula | C_62_H_92_N_4_Yb_2_ ·0.6C_7_H_8_ | C_64_H_96_N_4_Yb_2_ | C_128_H_184_N_8_Yb_4_ · C_6_H_6_ |
| Formula weight (g/mol) | 1294.75 | 1267.52 | 2605.09 |
| Temperature (K) | 150(2) | 120(2) | 150(2) |
| Crystal system | Triclinic | Monoclinic | Monoclinic |
| Space group | *P-*1 | *P*2_1_/c | *P*2_1_/n |
| Unit cell dimensions |  |  |  |
| a (Å) | 8.9244(7) | 18.5744(2) | 18.4011(6) |
| b (Å) | 14.0012(7) | 14.87030(10) | 16.5304(5) |
| c (Å) | 14.5450(6) | 22.9981(2) | 21.0634(6) |
| α (°) | 92.393(4) | 90 | 90 |
| β (°) | 99.833(5) | 105.0910(10) | 104.094(3) |
| γ (°) | 101.713(5) | 90 | 90 |
| Volume (Å^3^) | 1747.94(18) | 6133.17(10) | 6214.1(3) |
| Z | 1 | 4 | 2 |
| Calculated density (Mg/m^3^) | 1.230 | 1.373 | 1.392 |
| Absorption coefficient (mm^-1^) | 5.075 | 5.772 | 5.715 |
| F(000) | 662 | 2592 | 2660 |
| Crystal size (mm^3^) | 0.083 × 0.047 × 0.023 | 0.226 × 0.098 × 0.065 | 0.066 × 0.047 × 0.034 |
| Theta range for data collection | 4.296 to 74.245° | 3.862 to 73.477° | 3.645 to 73.511° |
| Index ranges | -11 ≤ h ≤ 11  -17 ≤ k ≤ 17  -17 ≤ l ≤ 18 | -22 ≤ h ≤ 23  -16 ≤ k ≤ 18  -28 ≤ l ≤ 28 | -22 ≤ h ≤ 22  -20 ≤ k ≤ 20  -22 ≤ l ≤ 26 |
| Reflections collected | 13108 | 39865 | 45687 |
| Independent reflections | 13108 R(int) = N/A | 12141 R(int) = 0.0267 | 12403 R(int) = 0.0538 |
| Completeness to theta | 99.8% | 98.9% | 99.9% |
| Data / restraints / parameters | 13108 / 42 / 371 | 12141 / 0 / 661 | 12403 / 9 / 673 |
| Goodness-of-fit on F2 | 0.965 | 1.039 | 1.048 |
| Final R indices [I>2sigma(I)] | R1 = 0.0540  wR2 = 0.1492 | R1 = 0.0260  wR2 = 0.0629 | R1 = 0.0446  wR2 = 0.1035 |
| R indices (all data) | R1 = 0.0745  wR2 = 0.1584 | R1 = 0.0297  wR2 = 0.0647 | R1 = 0.0621  wR2 = 0.1175 |
| Largest diff. peak  and hole (e.Å^-3^) | 1.315 and ‑2.203 | 0.700 and ‑0.859 | 3.891 and ‑2.045 |

**Supplementary Figure 60:** ORTEP representation (ellipsoid 30% probability) of [Yb{N(SiMe_3_)_2_}_2_]_2_**.** Hydrogen atoms have been omitted for clarity.

**Supplementary Figure 61:** ORTEP (ellipsoid 30% probability) of the tetrameric ytterbium(II) allyl complex [(BDI^Dipp^)Yb(CH_2_C(H)CH_2_)]_4_ (**6**) (16.7 mg, 41% isolated yield). Hydrogen atoms except those of the bridging allyl groups have been omitted for clarity. Due to the extreme insolubility of the Yb(II) allyl complex **6** in hydrocarbon solvents (benzene, toluene, cyclohexane methylcyclohexane) even at elevated temperatures and compound degradation in coordinating solvents (THF, Et_2_O, Py), we have been unable to obtain accurate NMR spectroscopic data.

## 1.6 Computational details

All calculations were carried out at the DFT level of theory using the hybrid functional B3PW91^9,10^ with the Gaussian 09^11^ suite of programs. The Yb was represented with a small-core Stuttgart-Dresden relativistic effective core potential associated with the adapted basis set.^12-14^ All the other atoms C, N and H were described with a 6-31G (d,p), double –ζ quality basis set.^15^ The nature of the extrema (minimum and transition states) was established with analytical frequencies calculations and geometry optimizations were computed without any symmetry constraints. The enthalpy energy was computed at T=298 k in the gas phase.

#### **Supplementary Figure 62:** Computed enthalpy profile for the alkylation of benzene catalysed by **2**, including the alternative reaction pathway for the polymerization of ethene.

#### **Supplementary Figure 63:** Computed enthalpy profile for the hydroarylation of propene with benzene catalysed by **2**, including the catalyst deactivation pathways (**C** – **E**) to [BDI^Dipp^Yb(CH_2_C(H)CH_2_)]_4_ (**6**) in red.

Cartesian coordinates of all optimized structures

| 148 |  |  |  |
| --- | --- | --- | --- |
| Complex2 |  |  |  |
| Yb | 7.473647 | 8.530007 | 8.625201 |
| Yb | 8.40082 | 8.091744 | 11.91387 |
| N | 7.398919 | 9.49924 | 6.326392 |
| N | 9.234946 | 10.29349 | 8.670935 |
| N | 8.475889 | 7.122257 | 14.21262 |
| N | 6.639932 | 6.327944 | 11.86805 |
| C | 8.75467 | 7.214609 | 3.082273 |
| C | 12.72753 | 9.326231 | 8.734593 |
| C | 4.917502 | 11.72683 | 5.754496 |
| C | 8.6218 | 6.018174 | 5.29081 |
| C | 3.149753 | 10.32147 | 6.818241 |
| C | 8.910061 | 13.68073 | 10.78332 |
| C | 4.539714 | 7.375151 | 4.046598 |
| C | 6.965653 | 12.25691 | 11.47527 |
| C | 8.319173 | 7.328049 | 4.550852 |
| C | 5.882122 | 7.023167 | 3.98319 |
| C | 4.153235 | 8.459079 | 4.830113 |
| C | 10.67722 | 12.21606 | 8.035499 |
| C | 11.94271 | 7.181163 | 9.761453 |
| C | 4.630428 | 10.37319 | 6.425496 |
| C | 6.857128 | 7.735702 | 4.689008 |
| C | 11.506 | 8.566553 | 9.280004 |
| C | 8.014011 | 10.76428 | 4.283711 |
| C | 5.083595 | 9.192114 | 5.572748 |
| C | 8.244133 | 12.30181 | 10.62933 |
| C | 6.455003 | 8.821566 | 5.507962 |
| C | 10.78607 | 9.391126 | 10.33734 |
| C | 11.16828 | 9.291374 | 11.68225 |
| C | 9.628949 | 11.15503 | 7.741754 |
| C | 9.648534 | 11.04849 | 12.29839 |
| C | 9.772972 | 10.32757 | 9.977807 |
| C | 10.59803 | 10.0985 | 12.66896 |
| C | 9.140024 | 11.20033 | 6.422172 |
| C | 8.155475 | 10.44438 | 5.763974 |
| C | 9.22703 | 11.19002 | 10.96923 |
| C | 7.119365 | 9.407311 | 17.45642 |
| C | 3.147504 | 7.295114 | 11.80538 |
| C | 10.95746 | 4.89513 | 14.78464 |
| C | 7.25232 | 10.60315 | 15.24759 |
| C | 12.7254 | 6.300829 | 13.7217 |
| C | 6.964583 | 2.940983 | 9.754987 |
| C | 11.33436 | 9.246888 | 16.49286 |
| C | 8.908892 | 4.365091 | 9.063279 |
| C | 7.55501 | 9.29349 | 15.98791 |
| C | 9.991892 | 9.598688 | 16.556 |
| C | 11.72114 | 8.163 | 15.70945 |
| C | 5.19805 | 4.405018 | 12.50327 |
| C | 3.931776 | 9.440306 | 10.77831 |
| C | 11.24459 | 6.24889 | 14.11391 |
| C | 9.01712 | 8.885985 | 15.85003 |
| C | 4.368773 | 8.054945 | 11.25958 |
| C | 7.860618 | 5.857346 | 16.25532 |
| C | 10.79103 | 7.429806 | 14.96666 |
| C | 7.630441 | 4.319909 | 9.90925 |
| C | 9.419551 | 7.800129 | 15.03119 |
| C | 5.088477 | 7.230457 | 10.20203 |
| C | 4.705979 | 7.33032 | 8.857199 |
| C | 6.246039 | 5.466268 | 12.79716 |
| C | 6.225721 | 5.57337 | 8.24056 |
| C | 6.101686 | 6.294022 | 10.56126 |
| C | 5.276091 | 6.523346 | 7.870278 |
| C | 6.734944 | 5.420987 | 14.11675 |
| C | 7.71936 | 6.177069 | 14.775 |
| C | 6.647462 | 5.431709 | 9.569622 |
| H | 7.014796 | 9.251653 | 10.6541 |
| H | 8.268048 | 6.372101 | 2.578606 |
| H | 9.835863 | 7.049992 | 3.017853 |
| H | 8.514015 | 8.119377 | 2.515925 |
| H | 12.45318 | 10.28521 | 8.291678 |
| H | 13.22893 | 8.731843 | 7.962636 |
| H | 13.45402 | 9.519892 | 9.532334 |
| H | 5.985851 | 11.89457 | 5.611589 |
| H | 4.533335 | 12.54521 | 6.373949 |
| H | 4.423464 | 11.7846 | 4.777988 |
| H | 8.410073 | 6.101853 | 6.360895 |
| H | 9.677185 | 5.745106 | 5.180719 |
| H | 8.018829 | 5.192181 | 4.895832 |
| H | 2.498307 | 10.52807 | 5.961385 |
| H | 2.938324 | 11.08623 | 7.572154 |
| H | 2.856226 | 9.350543 | 7.228844 |
| H | 9.16086 | 13.8766 | 11.83224 |
| H | 8.22609 | 14.47127 | 10.45508 |
| H | 9.831408 | 13.76642 | 10.20269 |
| H | 3.797637 | 6.814308 | 3.484871 |
| H | 6.501518 | 11.26928 | 11.41708 |
| H | 6.251095 | 13.00635 | 11.11672 |
| H | 7.170991 | 12.48664 | 12.52724 |
| H | 8.925992 | 8.10947 | 5.018023 |
| H | 6.182738 | 6.182096 | 3.363159 |
| H | 3.103546 | 8.733598 | 4.866144 |
| H | 11.14397 | 12.08928 | 9.013059 |
| H | 10.21877 | 13.20987 | 7.99836 |
| H | 11.45759 | 12.20019 | 7.268567 |
| H | 12.7493 | 7.23394 | 10.50145 |
| H | 12.32664 | 6.600385 | 8.915942 |
| H | 11.09965 | 6.633613 | 10.19199 |
| H | 5.22581 | 10.34767 | 7.349702 |
| H | 10.79807 | 8.427876 | 8.455832 |
| H | 8.672409 | 10.11523 | 3.695355 |
| H | 8.309263 | 11.79621 | 4.081013 |
| H | 6.997586 | 10.60879 | 3.917966 |
| H | 7.956067 | 12.17214 | 9.58148 |
| H | 11.94169 | 8.58407 | 11.96138 |
| H | 9.236602 | 11.70825 | 13.05638 |
| H | 10.91442 | 10.00694 | 13.70324 |
| H | 9.617997 | 11.95108 | 5.800671 |
| H | 8.859698 | 7.370178 | 9.884995 |
| H | 7.60585 | 10.25002 | 17.95988 |
| H | 6.038149 | 9.571827 | 17.52069 |
| H | 7.360067 | 8.502741 | 18.02306 |
| H | 3.422149 | 6.336226 | 12.24831 |
| H | 2.646217 | 7.889484 | 12.57743 |
| H | 2.420822 | 7.101262 | 11.00786 |
| H | 9.889078 | 4.727199 | 14.92708 |
| H | 11.34201 | 4.076875 | 14.16525 |
| H | 11.45111 | 4.837313 | 15.76134 |
| H | 7.464169 | 10.51924 | 14.17755 |
| H | 6.196898 | 10.87613 | 15.35751 |
| H | 7.855168 | 11.4293 | 15.64242 |
| H | 13.37657 | 6.094126 | 14.57874 |
| H | 12.93717 | 5.536236 | 12.96772 |
| H | 13.01899 | 7.271865 | 13.31139 |
| H | 6.713857 | 2.745271 | 8.706023 |
| H | 7.648565 | 2.150412 | 10.08313 |
| H | 6.043201 | 2.855164 | 10.33555 |
| H | 12.07625 | 9.807863 | 17.0547 |
| H | 9.372935 | 5.352747 | 9.121668 |
| H | 9.623526 | 3.615634 | 9.421645 |
| H | 8.703545 | 4.135591 | 8.011268 |
| H | 6.94832 | 8.511876 | 15.52089 |
| H | 9.691036 | 10.43974 | 17.17594 |
| H | 12.77088 | 7.888653 | 15.67358 |
| H | 4.731018 | 4.532008 | 11.52587 |
| H | 5.656878 | 3.411357 | 12.53988 |
| H | 4.417855 | 4.420289 | 13.27039 |
| H | 3.124989 | 9.387476 | 10.03853 |
| H | 3.548012 | 10.02099 | 11.62396 |
| H | 4.774654 | 9.987959 | 10.34757 |
| H | 10.64951 | 6.274473 | 13.18952 |
| H | 5.076915 | 8.19368 | 12.08356 |
| H | 7.202246 | 6.506561 | 16.84352 |
| H | 7.565212 | 4.825482 | 16.45813 |
| H | 8.87702 | 6.01276 | 16.62117 |
| H | 7.918538 | 4.449378 | 10.95711 |
| H | 3.932473 | 8.037611 | 8.578295 |
| H | 6.637554 | 4.913724 | 7.482426 |
| H | 4.959504 | 6.615024 | 6.83608 |
| H | 6.257056 | 4.670153 | 14.73822 |
|  |  |  |  |
| 148 |  |  |  |
| complex2' |  |  |  |
| Yb | 5.756809 | 9.246921 | 4.233161 |
| Yb | 8.132919 | 6.653342 | 4.382964 |
| N | 5.593712 | 11.60026 | 4.539409 |
| N | 3.453622 | 9.617409 | 3.765271 |
| N | 10.46739 | 6.277528 | 4.116844 |
| N | 8.270566 | 4.313993 | 4.793383 |
| C | 8.16986 | 11.30518 | 1.722413 |
| C | 6.924848 | 5.502535 | 8.280791 |
| C | 6.643803 | 10.54683 | 8.16672 |
| C | 6.320094 | 1.997111 | 1.952271 |
| C | 10.33904 | 8.787946 | 7.002586 |
| C | 12.68424 | 5.217237 | 4.044922 |
| C | 3.250235 | 12.0174 | 4.061088 |
| C | 3.352686 | 7.209247 | 6.771091 |
| C | 10.64607 | 3.891045 | 4.527695 |
| C | 11.18255 | 5.159076 | 4.231061 |
| C | 4.580338 | 8.238542 | 0.29247 |
| C | 9.626212 | 7.517062 | 0.494527 |
| C | 6.017543 | 12.97872 | 8.384431 |
| C | 6.224271 | 11.81315 | 7.405261 |
| C | 7.78099 | 13.79569 | 1.777153 |
| C | 7.466786 | 12.45546 | 2.458083 |
| C | 7.216938 | 12.16186 | 6.304089 |
| C | 8.513476 | 12.55196 | 6.652125 |
| C | 9.454393 | 12.88779 | 5.685449 |
| C | 9.099597 | 12.84393 | 4.342313 |
| C | 7.814961 | 12.46787 | 3.940239 |
| C | 6.864245 | 12.11197 | 4.930708 |
| C | 2.223047 | 9.026917 | -0.12412 |
| C | 3.309399 | 8.808961 | 0.939707 |
| C | 0.93071 | 7.870979 | 6.532281 |
| C | 2.334481 | 7.981301 | 5.919453 |
| C | 2.821159 | 7.931703 | 2.084931 |
| C | 2.332252 | 6.652461 | 1.805214 |
| C | 1.871834 | 5.814388 | 2.814443 |
| C | 1.888639 | 6.25781 | 4.131557 |
| C | 2.360748 | 7.530279 | 4.465286 |
| C | 2.842119 | 8.375042 | 3.432606 |
| C | 4.747798 | 13.90567 | 4.644633 |
| C | 4.553083 | 12.42254 | 4.410741 |
| C | 1.256445 | 10.67376 | 3.452051 |
| C | 2.735159 | 10.73914 | 3.770497 |
| C | 12.78283 | 8.168325 | 6.99782 |
| C | 11.43875 | 8.001026 | 6.27458 |
| C | 11.99609 | 6.673971 | 0.325473 |
| C | 10.82536 | 6.961344 | 1.277596 |
| C | 11.52451 | 8.38857 | 4.804653 |
| C | 12.03109 | 9.642337 | 4.451259 |
| C | 12.14618 | 10.02771 | 3.120807 |
| C | 11.75142 | 9.149314 | 2.118235 |
| C | 11.23102 | 7.886833 | 2.416993 |
| C | 11.1087 | 7.503469 | 3.777462 |
| C | 7.543575 | 3.085639 | 8.638534 |
| C | 7.409683 | 4.2123 | 7.602684 |
| C | 9.108529 | 2.017523 | 5.05131 |
| C | 9.319365 | 3.492457 | 4.781587 |
| C | 5.937424 | 4.482034 | 1.763894 |
| C | 6.576446 | 3.364858 | 2.6019 |
| C | 4.794987 | 3.044664 | 4.360802 |
| C | 6.107261 | 3.411211 | 4.049741 |
| C | 6.973221 | 3.811654 | 5.099068 |
| C | 6.510055 | 3.814953 | 6.440225 |
| C | 5.190554 | 3.431739 | 6.69715 |
| C | 4.332269 | 3.052317 | 5.671509 |
| H | 7.892445 | 2.152645 | 8.185006 |
| H | 8.258409 | 3.368619 | 9.418992 |
| H | 6.586984 | 2.875935 | 9.129736 |
| H | 13.08118 | 9.220431 | 7.061583 |
| H | 12.71394 | 7.786065 | 8.022092 |
| H | 13.58644 | 7.627576 | 6.488084 |
| H | 5.930314 | 5.365733 | 8.720434 |
| H | 7.607046 | 5.792384 | 9.088216 |
| H | 6.860363 | 6.336991 | 7.573735 |
| H | 9.347675 | 8.625278 | 6.563034 |
| H | 10.28449 | 8.492898 | 8.056587 |
| H | 10.53094 | 9.865353 | 6.961356 |
| H | 6.386876 | 12.29291 | 2.37609 |
| H | 3.309933 | 2.758106 | 5.893164 |
| H | 0.738384 | 10.01872 | 4.160209 |
| H | 0.793216 | 11.66094 | 3.489812 |
| H | 1.088692 | 10.2479 | 2.458114 |
| H | 12.86553 | 6.273932 | 0.856438 |
| H | 11.69909 | 5.942022 | -0.43357 |
| H | 12.31888 | 7.579522 | -0.20012 |
| H | 4.124729 | 2.744424 | 3.559633 |
| H | 6.175296 | 5.48084 | 2.148706 |
| H | 6.288701 | 4.436751 | 0.726795 |
| H | 4.845652 | 4.396202 | 1.756016 |
| H | 8.857665 | 13.99651 | 1.752788 |
| H | 7.426996 | 13.78724 | 0.740513 |
| H | 7.30053 | 14.63365 | 2.292001 |
| H | 9.876158 | 8.471589 | 0.017522 |
| H | 9.32653 | 6.819819 | -0.29627 |
| H | 8.759505 | 7.689829 | 1.142089 |
| H | 1.304156 | 9.435848 | 0.307585 |
| H | 2.575773 | 9.725719 | -0.89056 |
| H | 1.961188 | 8.090862 | -0.62961 |
| H | 12.34389 | 10.32837 | 5.234274 |
| H | 7.895256 | 10.32257 | 2.124395 |
| H | 7.909351 | 11.31001 | 0.657951 |
| H | 9.258605 | 11.38931 | 1.805589 |
| H | 5.713609 | 13.89569 | 7.869943 |
| H | 5.24029 | 12.729 | 9.114961 |
| H | 6.933305 | 13.20205 | 8.942922 |
| H | 7.602047 | 10.6948 | 8.677469 |
| H | 5.897967 | 10.29163 | 8.92817 |
| H | 6.756817 | 9.685943 | 7.498433 |
| H | 8.433656 | 1.582794 | 4.306514 |
| H | 10.05011 | 1.466765 | 5.028829 |
| H | 8.635073 | 1.86088 | 6.025127 |
| H | 1.496821 | 4.82301 | 2.574931 |
| H | 0.602454 | 6.828603 | 6.609253 |
| H | 0.922836 | 8.292231 | 7.543496 |
| H | 0.186574 | 8.408454 | 5.936023 |
| H | 8.407589 | 4.404571 | 7.194745 |
| H | 4.386954 | 7.256236 | -0.15338 |
| H | 4.936381 | 8.90086 | -0.50493 |
| H | 5.390497 | 8.114631 | 1.019692 |
| H | 5.259971 | 11.61074 | 6.927205 |
| H | 7.659572 | 3.527412 | 2.602842 |
| H | 5.480975 | 14.31367 | 3.940947 |
| H | 3.81313 | 14.45663 | 4.529382 |
| H | 5.143803 | 14.09458 | 5.646931 |
| H | 1.526059 | 5.603002 | 4.919824 |
| H | 11.17426 | 6.939103 | 6.318404 |
| H | 5.249263 | 1.790835 | 1.848107 |
| H | 6.757775 | 1.965465 | 0.948567 |
| H | 6.757756 | 1.182518 | 2.537943 |
| H | 2.61646 | 9.039341 | 5.939995 |
| H | 4.830405 | 3.430333 | 7.723177 |
| H | 12.54818 | 11.00467 | 2.866285 |
| H | 4.380555 | 7.33865 | 6.411626 |
| H | 3.322325 | 7.548951 | 7.812551 |
| H | 3.144605 | 6.134167 | 6.759462 |
| H | 11.84994 | 9.448306 | 1.077469 |
| H | 2.52443 | 12.82067 | 4.009664 |
| H | 3.56092 | 9.788471 | 1.359954 |
| H | 10.5208 | 6.007428 | 1.720929 |
| H | 10.45698 | 13.18838 | 5.977514 |
| H | 9.834979 | 13.10972 | 3.587348 |
| H | 2.313202 | 6.306973 | 0.774375 |
| H | 11.37509 | 3.090194 | 4.569776 |
| H | 8.789583 | 12.59391 | 7.703029 |
| H | 12.94372 | 5.643221 | 3.071246 |
| H | 13.13834 | 5.869518 | 4.798469 |
| H | 13.13777 | 4.228211 | 4.125953 |
| H | 6.887282 | 7.976776 | 5.699608 |
| H | 6.997887 | 7.918702 | 2.917401 |
|  |  |  |  |
| 154 |  |  |  |
| TS-first-insertion | |  |  |
| Yb | 5.768409 | 9.220634 | 3.525503 |
| Yb | 8.268079 | 6.523891 | 3.907441 |
| N | 10.59838 | 6.216176 | 4.252532 |
| N | 5.809707 | 11.52083 | 4.202015 |
| N | 8.376352 | 4.179142 | 4.229718 |
| N | 3.465769 | 9.399596 | 4.192275 |
| C | 6.216206 | 2.762894 | 7.460735 |
| C | 12.15555 | 8.218725 | 7.532799 |
| C | 5.803843 | 5.197364 | 6.954426 |
| C | 9.775056 | 8.839527 | 6.978431 |
| C | 7.890944 | 12.29133 | 2.256574 |
| C | 4.459378 | 2.780249 | 3.479194 |
| C | 1.319449 | 10.52906 | 3.717166 |
| C | 12.95658 | 6.593352 | 0.8853 |
| C | 5.398224 | 2.81937 | 2.45465 |
| C | 7.380185 | 4.362962 | 0.530772 |
| C | 8.185234 | 13.57016 | 1.459499 |
| C | 11.41787 | 8.376078 | 5.112576 |
| C | 10.558 | 7.29784 | 0.5557 |
| C | 5.602222 | 9.382027 | 0.959326 |
| C | 1.053724 | 7.425086 | 1.413413 |
| C | 11.96408 | 9.634802 | 4.845132 |
| C | 8.77711 | 11.14007 | 1.761711 |
| C | 5.700397 | 13.27945 | 7.8949 |
| C | 6.139642 | 3.558419 | 5.055387 |
| C | 6.551391 | 10.90599 | 7.993424 |
| C | 9.399613 | 3.395674 | 4.56879 |
| C | 9.163838 | 1.912551 | 4.757186 |
| C | 2.83046 | 10.52774 | 3.874373 |
| C | 1.329017 | 5.988019 | 5.51532 |
| C | 2.043357 | 10.1002 | 7.37603 |
| C | 6.509148 | 3.915541 | 6.488323 |
| C | 3.373893 | 6.470703 | 1.63112 |
| C | 6.107923 | 12.04942 | 7.070686 |
| C | 7.708516 | 3.250224 | 1.536616 |
| C | 4.886507 | 13.77914 | 3.88059 |
| C | 1.845418 | 6.903083 | 6.426602 |
| C | 11.00248 | 8.030493 | 6.535525 |
| C | 7.799097 | 1.893999 | 0.822915 |
| C | 11.27802 | 7.452123 | 4.046295 |
| C | 11.28454 | 5.124544 | 4.590622 |
| C | 4.745929 | 12.26943 | 3.909313 |
| C | 3.084745 | 9.022645 | 7.03403 |
| C | 11.71533 | 7.801231 | 2.742676 |
| C | 4.835878 | 3.144717 | 4.766242 |
| C | 2.715114 | 8.263455 | 4.615804 |
| C | 12.38316 | 9.987634 | 3.567983 |
| C | 6.539393 | 8.346803 | 0.877636 |
| C | 3.579596 | 8.346864 | 8.317358 |
| C | 7.085908 | 3.619549 | 4.001152 |
| C | 2.198561 | 7.329819 | 3.684711 |
| C | 12.26184 | 9.070095 | 2.530737 |
| C | 6.71307 | 3.231509 | 2.688512 |
| C | 3.445469 | 11.7814 | 3.649167 |
| C | 7.187061 | 12.3766 | 6.049581 |
| C | 2.546398 | 8.036785 | 6.00719 |
| C | 2.379007 | 7.501005 | 2.184701 |
| C | 11.60582 | 6.829362 | 1.575525 |
| C | 9.427325 | 13.22036 | 5.614312 |
| C | 9.237623 | 13.01003 | 4.254462 |
| C | 1.515867 | 6.204142 | 4.157098 |
| C | 10.72082 | 3.844759 | 4.757451 |
| C | 8.400726 | 12.90647 | 6.497036 |
| C | 7.00278 | 12.15257 | 4.66209 |
| C | 8.039952 | 12.48833 | 3.756724 |
| C | 12.77874 | 5.226933 | 4.810814 |
| H | 7.093349 | 7.774319 | 2.224248 |
| H | 6.203951 | 7.361196 | 0.562898 |
| H | 7.54561 | 8.576003 | 0.53442 |
| H | 4.544867 | 9.170986 | 0.828045 |
| H | 5.911386 | 10.41223 | 0.807472 |
| H | 6.940479 | 7.955209 | 4.91431 |
| H | 6.697811 | 1.831435 | 7.146504 |
| H | 6.580854 | 3.010529 | 8.463543 |
| H | 5.141423 | 2.56783 | 7.542242 |
| H | 12.43739 | 9.272841 | 7.629488 |
| H | 11.85878 | 7.86725 | 8.526902 |
| H | 13.05004 | 7.664447 | 7.230955 |
| H | 4.714935 | 5.084303 | 6.921074 |
| H | 6.087089 | 5.435934 | 7.985978 |
| H | 6.058542 | 6.060926 | 6.32923 |
| H | 8.909577 | 8.664854 | 6.32896 |
| H | 9.484721 | 8.570377 | 8.00027 |
| H | 9.979495 | 9.915259 | 6.958788 |
| H | 6.847766 | 12.0194 | 2.064816 |
| H | 3.440625 | 2.461618 | 3.276798 |
| H | 0.910114 | 11.53661 | 3.810107 |
| H | 1.048033 | 10.14742 | 2.726116 |
| H | 0.835843 | 9.880899 | 4.451239 |
| H | 13.71915 | 6.260628 | 1.596161 |
| H | 12.86062 | 5.825982 | 0.109437 |
| H | 13.32934 | 7.503184 | 0.402536 |
| H | 5.104801 | 2.522312 | 1.450717 |
| H | 7.367398 | 5.350349 | 1.007948 |
| H | 8.118487 | 4.391604 | -0.27813 |
| H | 6.393218 | 4.21045 | 0.080203 |
| H | 9.234108 | 13.87308 | 1.551053 |
| H | 7.98198 | 13.41104 | 0.394757 |
| H | 7.569368 | 14.40872 | 1.799063 |
| H | 10.83517 | 8.26348 | 0.118692 |
| H | 10.45526 | 6.575759 | -0.26185 |
| H | 9.57171 | 7.424421 | 1.018031 |
| H | 0.314362 | 8.127792 | 1.809164 |
| H | 1.213652 | 7.65901 | 0.355089 |
| H | 0.614168 | 6.422865 | 1.462405 |
| H | 12.06031 | 10.35294 | 5.654999 |
| H | 8.554928 | 10.20265 | 2.285024 |
| H | 8.63562 | 10.96978 | 0.688559 |
| H | 9.836591 | 11.35455 | 1.935731 |
| H | 5.371744 | 14.10534 | 7.256239 |
| H | 4.875985 | 13.02931 | 8.571824 |
| H | 6.529511 | 13.64756 | 8.509457 |
| H | 7.459476 | 11.17172 | 8.546385 |
| H | 5.772078 | 10.67887 | 8.72876 |
| H | 6.760015 | 9.994917 | 7.424584 |
| H | 10.07311 | 1.39708 | 5.070146 |
| H | 8.384846 | 1.734565 | 5.504873 |
| H | 8.809195 | 1.456689 | 3.826819 |
| H | 0.78551 | 5.113757 | 5.863849 |
| H | 1.134304 | 9.645634 | 7.786884 |
| H | 2.439215 | 10.79611 | 8.124632 |
| H | 1.761771 | 10.68434 | 6.49656 |
| H | 7.588669 | 4.099495 | 6.514068 |
| H | 3.003059 | 5.450196 | 1.773728 |
| H | 3.540971 | 6.624866 | 0.559076 |
| H | 4.343451 | 6.529987 | 2.137455 |
| H | 5.224945 | 11.70933 | 6.521471 |
| H | 8.695919 | 3.466223 | 1.9577 |
| H | 5.733502 | 14.08651 | 3.260533 |
| H | 3.981271 | 14.25959 | 3.506114 |
| H | 5.085408 | 14.16159 | 4.887255 |
| H | 1.700075 | 6.731404 | 7.489114 |
| H | 10.72705 | 6.970449 | 6.549482 |
| H | 6.861526 | 1.637173 | 0.318145 |
| H | 8.58608 | 1.915372 | 0.061207 |
| H | 8.02898 | 1.085195 | 1.523403 |
| H | 3.938418 | 9.528833 | 6.568543 |
| H | 4.10115 | 3.110002 | 5.56628 |
| H | 12.80772 | 10.97056 | 3.383306 |
| H | 4.297253 | 7.550132 | 8.102796 |
| H | 4.072207 | 9.08185 | 8.961401 |
| H | 2.757556 | 7.916222 | 8.900322 |
| H | 12.59942 | 9.343268 | 1.533842 |
| H | 2.746401 | 12.55438 | 3.35002 |
| H | 2.801747 | 8.496962 | 2.017328 |
| H | 11.26973 | 5.868377 | 1.97836 |
| H | 10.36321 | 13.63243 | 5.982425 |
| H | 10.03522 | 13.25944 | 3.559025 |
| H | 1.118586 | 5.487392 | 3.442622 |
| H | 11.42495 | 3.07253 | 5.044456 |
| H | 8.542337 | 13.07668 | 7.561807 |
| H | 13.21427 | 4.258784 | 5.062307 |
| H | 13.27868 | 5.612728 | 3.916789 |
| H | 13.00375 | 5.930396 | 5.61882 |
|  |  |  |  |
| 154 |  |  |  |
| Product-first-insertion | | |  |
| Yb | 8.078995 | 6.641005 | 4.286092 |
| Yb | 5.805075 | 9.244204 | 3.995392 |
| N | 3.45136 | 9.601626 | 3.803214 |
| N | 8.123138 | 4.262185 | 4.649307 |
| N | 5.737803 | 11.63688 | 4.0204 |
| N | 10.44709 | 6.390324 | 4.524546 |
| C | 12.61666 | 5.251752 | 4.286285 |
| C | 7.513314 | 12.46643 | 2.547461 |
| C | 7.03629 | 12.19959 | 3.858146 |
| C | 9.19062 | 12.8506 | 4.757888 |
| C | 10.50981 | 3.966544 | 4.23064 |
| C | 1.933681 | 6.901767 | 1.732904 |
| C | 8.821638 | 12.93145 | 2.387613 |
| C | 9.662497 | 13.11831 | 3.479046 |
| C | 11.33793 | 8.204282 | 2.353073 |
| C | 2.835003 | 9.13063 | 0.975193 |
| C | 2.389004 | 7.467505 | 4.438517 |
| C | 7.884422 | 12.40145 | 4.976089 |
| C | 3.351204 | 11.97107 | 4.342734 |
| C | 5.962766 | 3.199349 | 4.076994 |
| C | 12.25033 | 9.651083 | 4.191707 |
| C | 2.51629 | 8.125252 | 2.074785 |
| C | 6.882888 | 3.676637 | 5.04587 |
| C | 3.65463 | 6.839861 | 6.540243 |
| C | 7.669082 | 6.741113 | 1.406616 |
| C | 12.5237 | 9.951606 | 5.519487 |
| C | 2.755031 | 8.410504 | 3.445385 |
| C | 5.292265 | 3.1296 | 6.795041 |
| C | 11.59281 | 8.47132 | 3.82923 |
| C | 2.579724 | 7.745036 | 5.923128 |
| C | 4.687384 | 12.41603 | 4.270855 |
| C | 11.10164 | 5.240203 | 4.338533 |
| C | 11.19356 | 7.565224 | 4.843564 |
| C | 6.360302 | 1.757339 | 2.029707 |
| C | 11.08137 | 6.931079 | 7.336845 |
| C | 1.821227 | 6.253009 | 4.040603 |
| C | 4.880102 | 13.89826 | 4.530053 |
| C | 6.277547 | 3.18676 | 2.58764 |
| C | 7.41297 | 12.16634 | 6.404472 |
| C | 3.729713 | 8.540646 | -0.12346 |
| C | 7.494869 | 4.110314 | 7.507358 |
| C | 1.26352 | 7.615297 | 6.704705 |
| C | 1.59263 | 5.963971 | 2.70112 |
| C | 2.771832 | 10.71239 | 4.091818 |
| C | 9.113474 | 1.990019 | 4.645954 |
| C | 9.200882 | 3.498325 | 4.491889 |
| C | 8.143089 | 10.98561 | 7.05972 |
| C | 6.538512 | 3.640976 | 6.420671 |
| C | 7.558376 | 13.42979 | 7.266724 |
| C | 7.291714 | 11.56475 | 0.17261 |
| C | 12.13514 | 9.058313 | 6.510537 |
| C | 1.551163 | 9.707218 | 0.357185 |
| C | 6.825418 | 7.709236 | 2.252356 |
| C | 10.37816 | 9.245099 | 1.759387 |
| C | 11.47466 | 7.86595 | 6.20171 |
| C | 6.100627 | 13.67843 | 0.844337 |
| C | 5.258227 | 4.006606 | 1.784503 |
| C | 4.729681 | 2.69858 | 4.505444 |
| C | 12.63807 | 8.162248 | 1.53534 |
| C | 1.259663 | 10.66359 | 4.186576 |
| C | 4.387621 | 2.659037 | 5.851419 |
| C | 6.614268 | 12.31277 | 1.327808 |
| C | 10.02416 | 7.573368 | 8.246335 |
| C | 6.889572 | 5.240124 | 8.35221 |
| C | 12.29567 | 6.48357 | 8.164054 |
| C | 7.938626 | 2.942969 | 8.402149 |
| H | 13.01346 | 4.283206 | 3.97819 |
| H | 13.02996 | 5.493044 | 5.271294 |
| H | 12.98475 | 6.01952 | 3.601078 |
| H | 9.848353 | 12.99875 | 5.61062 |
| H | 11.22335 | 3.166649 | 4.062123 |
| H | 1.741331 | 6.680545 | 0.685864 |
| H | 9.187701 | 13.15384 | 1.388676 |
| H | 10.67819 | 13.47598 | 3.333606 |
| H | 10.8667 | 7.217971 | 2.273794 |
| H | 3.378603 | 9.960251 | 1.439671 |
| H | 2.636785 | 12.75157 | 4.581428 |
| H | 12.55603 | 10.34638 | 3.413712 |
| H | 3.398688 | 5.781056 | 6.429254 |
| H | 3.76633 | 7.049592 | 7.609994 |
| H | 4.634933 | 6.991296 | 6.074456 |
| H | 13.03865 | 10.87232 | 5.780114 |
| H | 5.03082 | 3.094188 | 7.849838 |
| H | 2.920793 | 8.781424 | 6.022804 |
| H | 7.107701 | 1.156752 | 2.555503 |
| H | 6.630131 | 1.776552 | 0.967904 |
| H | 5.39758 | 1.241541 | 2.1173 |
| H | 10.63801 | 6.035565 | 6.89025 |
| H | 1.548741 | 5.522388 | 4.798038 |
| H | 5.015574 | 14.07158 | 5.604165 |
| H | 4.004104 | 14.46908 | 4.214187 |
| H | 5.764384 | 14.29144 | 4.025059 |
| H | 7.262641 | 3.646696 | 2.455242 |
| H | 6.347382 | 11.91597 | 6.363756 |
| H | 4.666273 | 8.150651 | 0.285739 |
| H | 3.976936 | 9.309216 | -0.8643 |
| H | 3.231469 | 7.723517 | -0.65689 |
| H | 8.388867 | 4.503847 | 7.012228 |
| H | 0.469091 | 8.228549 | 6.268262 |
| H | 1.405122 | 7.92998 | 7.744464 |
| H | 0.907125 | 6.57961 | 6.722801 |
| H | 1.145084 | 5.016615 | 2.413049 |
| H | 8.087356 | 1.623518 | 4.623717 |
| H | 9.557101 | 1.690385 | 5.602162 |
| H | 9.687733 | 1.493927 | 3.858299 |
| H | 7.965216 | 10.0487 | 6.521033 |
| H | 7.797671 | 10.84543 | 8.090361 |
| H | 9.225154 | 11.15295 | 7.087421 |
| H | 8.611014 | 13.69323 | 7.417155 |
| H | 7.116992 | 13.26904 | 8.256437 |
| H | 7.065168 | 14.29286 | 6.809229 |
| H | 8.124615 | 12.13656 | -0.25075 |
| H | 6.57512 | 11.39053 | -0.63722 |
| H | 7.684324 | 10.5947 | 0.491772 |
| H | 12.35248 | 9.289271 | 7.550707 |
| H | 0.959257 | 8.921653 | -0.12609 |
| H | 1.794243 | 10.45751 | -0.40357 |
| H | 0.917037 | 10.18532 | 1.109277 |
| H | 9.417457 | 9.256687 | 2.284758 |
| H | 10.18421 | 9.038023 | 0.700746 |
| H | 10.79459 | 10.25488 | 1.830622 |
| H | 5.549368 | 14.20381 | 1.628998 |
| H | 5.430863 | 13.55803 | -0.01461 |
| H | 6.933453 | 14.31997 | 0.534053 |
| H | 4.258639 | 3.562303 | 1.837033 |
| H | 5.545211 | 4.048777 | 0.728112 |
| H | 5.175924 | 5.031675 | 2.155952 |
| H | 4.025277 | 2.325844 | 3.765994 |
| H | 13.13961 | 9.136159 | 1.532725 |
| H | 12.42572 | 7.898268 | 0.493439 |
| H | 13.34682 | 7.42803 | 1.930067 |
| H | 0.826931 | 10.03613 | 3.404106 |
| H | 0.823535 | 11.66211 | 4.123325 |
| H | 0.958365 | 10.22488 | 5.144859 |
| H | 3.426225 | 2.259301 | 6.162608 |
| H | 5.740772 | 11.7296 | 1.641042 |
| H | 10.40509 | 8.489994 | 8.710245 |
| H | 9.740688 | 6.887978 | 9.05256 |
| H | 9.118768 | 7.836682 | 7.690381 |
| H | 6.635531 | 6.109978 | 7.738015 |
| H | 7.596672 | 5.565617 | 9.12249 |
| H | 5.97644 | 4.913128 | 8.86166 |
| H | 13.06461 | 6.019025 | 7.539077 |
| H | 11.99307 | 5.753521 | 8.922916 |
| H | 12.76049 | 7.327072 | 8.686403 |
| H | 7.091636 | 2.522848 | 8.956091 |
| H | 8.680668 | 3.278716 | 9.135011 |
| H | 8.383312 | 2.133394 | 7.81563 |
| H | 6.851687 | 8.684724 | 1.723522 |
| H | 5.767191 | 7.383614 | 2.160982 |
| H | 7.260756 | 6.518694 | 0.410516 |
| H | 8.681817 | 7.128486 | 1.244946 |
| H | 7.792195 | 5.745677 | 1.872959 |
| H | 6.8839 | 8.041513 | 5.536836 |
|  |  |  |  |
| 160 |  |  |  |
| TS-second-insertion | |  |  |
| Yb | 8.159399 | 6.627472 | 4.205331 |
| Yb | 5.792916 | 9.314822 | 3.77714 |
| N | 3.474573 | 9.480969 | 4.374118 |
| N | 8.138997 | 4.229721 | 4.176561 |
| N | 5.812333 | 11.62172 | 4.450873 |
| N | 10.52681 | 6.320184 | 4.165452 |
| C | 7.559059 | 7.226919 | 6.797882 |
| C | 6.770308 | 8.113107 | 5.81792 |
| C | 12.60272 | 5.211824 | 4.8822 |
| C | 8.018166 | 12.69063 | 4.090829 |
| C | 6.970259 | 12.28554 | 4.953658 |
| C | 8.243772 | 13.11141 | 6.846999 |
| C | 10.44264 | 4.014179 | 4.968478 |
| C | 1.619402 | 6.234293 | 4.396554 |
| C | 9.153181 | 13.29791 | 4.637317 |
| C | 9.269469 | 13.52149 | 6.003123 |
| C | 11.42635 | 6.290062 | 1.377285 |
| C | 2.718022 | 7.382085 | 2.446909 |
| C | 2.295367 | 8.289279 | 6.172647 |
| C | 7.09688 | 12.48377 | 6.353039 |
| C | 3.49327 | 11.83278 | 3.710426 |
| C | 6.818979 | 3.250217 | 2.357354 |
| C | 12.48154 | 8.517222 | 1.872345 |
| C | 2.331869 | 7.33715 | 3.916319 |
| C | 6.951811 | 3.567438 | 3.734779 |
| C | 3.454527 | 8.82728 | 8.355092 |
| C | 6.726885 | 8.48989 | 1.183324 |
| C | 12.81836 | 9.576582 | 2.706129 |
| C | 2.682387 | 8.378596 | 4.810791 |
| C | 4.720289 | 2.699261 | 4.123122 |
| C | 11.75452 | 7.420265 | 2.342607 |
| C | 2.631156 | 9.377252 | 7.182575 |
| C | 4.771364 | 12.34492 | 4.038738 |
| C | 11.10341 | 5.221738 | 4.65938 |
| C | 11.35066 | 7.39221 | 3.702242 |
| C | 8.697737 | 2.197274 | 1.044498 |
| C | 11.32246 | 8.488099 | 6.032934 |
| C | 1.572314 | 7.172102 | 6.600686 |
| C | 4.914838 | 13.84814 | 3.880006 |
| C | 7.94705 | 3.498764 | 1.36682 |
| C | 6.003238 | 12.05251 | 7.31901 |
| C | 3.858618 | 6.399253 | 2.147305 |
| C | 5.960054 | 3.593949 | 6.11338 |
| C | 1.368642 | 10.0821 | 7.701876 |
| C | 1.233575 | 6.14499 | 5.728026 |
| C | 2.864546 | 10.59074 | 3.95307 |
| C | 8.972232 | 2.039649 | 4.968458 |
| C | 9.152592 | 3.517291 | 4.669781 |
| C | 6.55998 | 11.34841 | 8.562697 |
| C | 5.885839 | 3.28724 | 4.625527 |
| C | 5.117545 | 13.23879 | 7.731017 |
| C | 8.912065 | 11.33304 | 2.172577 |
| C | 12.42893 | 9.54008 | 4.038772 |
| C | 1.530943 | 7.121353 | 1.509215 |
| C | 5.755865 | 9.500277 | 1.201381 |
| C | 10.51641 | 6.773195 | 0.239523 |
| C | 11.70334 | 8.464125 | 4.560185 |
| C | 8.250631 | 13.73195 | 1.778103 |
| C | 7.462031 | 4.169934 | 0.075697 |
| C | 5.638778 | 2.650209 | 1.909885 |
| C | 12.69346 | 5.63636 | 0.805682 |
| C | 1.366624 | 10.56766 | 3.712107 |
| C | 4.589977 | 2.374566 | 2.779265 |
| C | 7.958265 | 12.46188 | 2.589131 |
| C | 10.31284 | 9.606525 | 6.326671 |
| C | 4.944031 | 4.673732 | 6.512134 |
| C | 12.54464 | 8.628369 | 6.95305 |
| C | 5.759238 | 2.338011 | 6.975199 |
| H | 7.378344 | 6.14891 | 6.64248 |
| H | 7.329066 | 7.390094 | 7.860034 |
| H | 8.648539 | 7.378227 | 6.721268 |
| H | 5.697919 | 7.968842 | 6.043185 |
| H | 6.972816 | 9.161345 | 6.104799 |
| H | 12.91712 | 6.06449 | 5.489992 |
| H | 13.13126 | 5.302544 | 3.927644 |
| H | 12.93072 | 4.292376 | 5.369156 |
| H | 8.335819 | 13.28168 | 7.916274 |
| H | 11.09397 | 3.267449 | 5.40894 |
| H | 1.355544 | 5.434508 | 3.709182 |
| H | 9.958068 | 13.60785 | 3.975487 |
| H | 10.1524 | 14.00905 | 6.407868 |
| H | 10.88284 | 5.523372 | 1.938434 |
| H | 3.082582 | 8.39309 | 2.236197 |
| H | 2.820066 | 12.58472 | 3.314544 |
| H | 12.79185 | 8.539457 | 0.830429 |
| H | 2.903886 | 8.06099 | 8.912136 |
| H | 3.700284 | 9.629376 | 9.059571 |
| H | 4.39137 | 8.382022 | 8.007831 |
| H | 13.38474 | 10.42041 | 2.321618 |
| H | 3.901144 | 2.485986 | 4.805214 |
| H | 3.242843 | 10.12378 | 6.66738 |
| H | 9.111315 | 1.73684 | 1.946229 |
| H | 9.526696 | 2.388325 | 0.353685 |
| H | 8.029068 | 1.467679 | 0.57347 |
| H | 10.84957 | 7.526821 | 6.264857 |
| H | 1.266552 | 7.108133 | 7.64234 |
| H | 5.465937 | 14.28592 | 4.715806 |
| H | 3.942679 | 14.33759 | 3.801861 |
| H | 5.484653 | 14.07898 | 2.972619 |
| H | 8.661075 | 4.174932 | 1.849844 |
| H | 5.370833 | 11.33578 | 6.784372 |
| H | 4.749054 | 6.611152 | 2.753465 |
| H | 4.151763 | 6.448042 | 1.092973 |
| H | 3.565717 | 5.368393 | 2.370754 |
| H | 6.966036 | 3.976394 | 6.320225 |
| H | 0.78554 | 10.52287 | 6.887801 |
| H | 1.635833 | 10.88744 | 8.395207 |
| H | 0.714499 | 9.385606 | 8.238759 |
| H | 0.669445 | 5.286018 | 6.081589 |
| H | 8.227232 | 1.579089 | 4.317385 |
| H | 8.627117 | 1.910988 | 6.001172 |
| H | 9.915785 | 1.499325 | 4.868435 |
| H | 7.209715 | 10.51112 | 8.291006 |
| H | 5.740285 | 10.95614 | 9.172999 |
| H | 7.136393 | 12.02932 | 9.198879 |
| H | 5.710012 | 14.01241 | 8.233353 |
| H | 4.333332 | 12.91388 | 8.42422 |
| H | 4.628976 | 13.6983 | 6.867314 |
| H | 9.952661 | 11.59105 | 2.395402 |
| H | 8.835973 | 11.13132 | 1.098423 |
| H | 8.69506 | 10.40163 | 2.708321 |
| H | 12.69645 | 10.36421 | 4.695332 |
| H | 1.163504 | 6.092768 | 1.592285 |
| H | 1.8287 | 7.277003 | 0.46654 |
| H | 0.691929 | 7.790063 | 1.725559 |
| H | 9.576279 | 7.18139 | 0.623499 |
| H | 10.27477 | 5.949821 | -0.44111 |
| H | 10.99933 | 7.559168 | -0.35134 |
| H | 7.603451 | 14.56165 | 2.078704 |
| H | 8.092529 | 13.54668 | 0.710068 |
| H | 9.287991 | 14.062 | 1.900917 |
| H | 6.807225 | 3.51334 | -0.50723 |
| H | 8.312634 | 4.425063 | -0.56441 |
| H | 6.906826 | 5.089815 | 0.283771 |
| H | 5.54106 | 2.390571 | 0.859197 |
| H | 13.26838 | 6.340638 | 0.19422 |
| H | 12.43139 | 4.784163 | 0.168975 |
| H | 13.35441 | 5.272235 | 1.598077 |
| H | 0.831556 | 10.23181 | 4.604795 |
| H | 1.117738 | 9.855481 | 2.918033 |
| H | 0.98799 | 11.5503 | 3.427252 |
| H | 3.680314 | 1.907246 | 2.41198 |
| H | 6.939195 | 12.14344 | 2.346459 |
| H | 10.7406 | 10.59232 | 6.119492 |
| H | 10.00755 | 9.592029 | 7.378646 |
| H | 9.410073 | 9.516625 | 5.713739 |
| H | 5.066418 | 5.589265 | 5.925989 |
| H | 5.048057 | 4.933298 | 7.571361 |
| H | 3.917012 | 4.330067 | 6.353519 |
| H | 13.29209 | 7.851698 | 6.764374 |
| H | 12.23993 | 8.555894 | 8.002768 |
| H | 13.03702 | 9.59788 | 6.821622 |
| H | 4.743171 | 1.941916 | 6.871827 |
| H | 5.912161 | 2.573267 | 8.034153 |
| H | 6.453224 | 1.538796 | 6.699423 |
| H | 6.050668 | 10.53837 | 1.078044 |
| H | 4.719779 | 9.263901 | 0.976501 |
| H | 7.750275 | 8.753992 | 0.924609 |
| H | 6.448519 | 7.50306 | 0.818947 |
| H | 7.156537 | 7.923343 | 2.53785 |
|  |  |  |  |
| 160 |  |  |  |
| Product-second-insertion | | |  |
| Yb | 5.791239 | 9.179727 | 4.17431 |
| Yb | 8.050114 | 6.667851 | 4.459063 |
| N | 10.42282 | 6.451994 | 4.756803 |
| N | 5.82606 | 11.57097 | 3.948594 |
| N | 8.094826 | 4.294927 | 4.836019 |
| N | 3.445071 | 9.468974 | 3.756572 |
| C | 8.545023 | 2.785486 | 8.177915 |
| C | 12.31231 | 6.02519 | 8.084934 |
| C | 7.120143 | 4.736878 | 8.892417 |
| C | 10.3392 | 7.442067 | 8.754982 |
| C | 6.312466 | 12.18034 | 1.14474 |
| C | 4.495746 | 2.547088 | 6.234675 |
| C | 1.302499 | 10.61977 | 4.20153 |
| C | 12.6236 | 8.440888 | 1.858866 |
| C | 4.722613 | 2.668757 | 4.870442 |
| C | 5.092684 | 4.292638 | 2.238925 |
| C | 5.570261 | 13.44394 | 0.682163 |
| C | 11.47194 | 7.811703 | 6.510088 |
| C | 10.33299 | 9.43343 | 2.175034 |
| C | 6.879767 | 7.77555 | 2.384718 |
| C | 1.986992 | 10.1488 | 0.375169 |
| C | 12.16556 | 8.964863 | 6.887057 |
| C | 6.949569 | 11.46803 | -0.05519 |
| C | 7.958658 | 13.53976 | 6.940204 |
| C | 5.839111 | 8.84265 | 7.023819 |
| C | 6.665104 | 3.550967 | 6.681227 |
| C | 8.70894 | 11.16543 | 6.570052 |
| C | 9.15915 | 3.561118 | 4.513956 |
| C | 9.039341 | 2.051089 | 4.410119 |
| C | 2.818418 | 10.58896 | 4.114563 |
| C | 1.379053 | 6.111447 | 2.200387 |
| C | 0.838551 | 7.48467 | 6.295369 |
| C | 7.713971 | 3.984915 | 7.695368 |
| C | 6.75481 | 7.883458 | 6.245984 |
| C | 3.874944 | 8.623279 | -0.30668 |
| C | 7.783623 | 12.28485 | 6.07216 |
| C | 6.088725 | 3.304709 | 2.862583 |
| C | 4.919757 | 13.78836 | 4.539506 |
| C | 1.501075 | 6.275365 | 3.573821 |
| C | 11.08484 | 6.798547 | 7.578398 |
| C | 5.965808 | 1.933285 | 2.181053 |
| C | 11.18367 | 7.599097 | 5.136031 |
| C | 11.05643 | 5.325234 | 4.422554 |
| C | 4.760269 | 12.29943 | 4.289691 |
| C | 2.226474 | 7.509979 | 5.637892 |
| C | 11.59481 | 8.558503 | 4.1776 |
| C | 5.467005 | 2.987117 | 7.126791 |
| C | 2.701089 | 8.359158 | 3.253811 |
| C | 12.56456 | 9.911884 | 5.951017 |
| C | 7.764076 | 6.790379 | 1.602828 |
| C | 3.11786 | 6.416537 | 6.242727 |
| C | 6.896549 | 3.661668 | 5.284197 |
| C | 2.584272 | 8.194133 | 1.84838 |
| C | 12.27244 | 9.703343 | 4.609546 |
| C | 5.909648 | 3.215167 | 4.370824 |
| C | 3.444262 | 11.81228 | 4.448178 |
| C | 8.007381 | 12.56115 | 4.59318 |
| C | 2.148024 | 7.387003 | 4.123778 |
| C | 3.118018 | 9.237273 | 0.87734 |
| C | 11.33195 | 8.387377 | 2.68904 |
| C | 9.475428 | 13.43812 | 2.863335 |
| C | 8.53035 | 13.10919 | 1.898463 |
| C | 1.91759 | 7.07142 | 1.351239 |
| C | 10.45101 | 4.064037 | 4.234497 |
| C | 9.209793 | 13.15523 | 4.196729 |
| C | 7.045127 | 12.23116 | 3.606739 |
| C | 7.319673 | 12.50036 | 2.240061 |
| C | 12.55963 | 5.345788 | 4.214276 |
| H | 7.71263 | 5.759379 | 1.995972 |
| H | 8.822999 | 7.081205 | 1.633243 |
| H | 7.514337 | 6.687149 | 0.537006 |
| H | 5.825793 | 7.517258 | 2.154251 |
| H | 7.019342 | 8.768097 | 1.911674 |
| H | 5.749045 | 8.629901 | 8.098862 |
| H | 4.80015 | 8.834367 | 6.652039 |
| H | 6.185004 | 9.88409 | 6.958711 |
| H | 7.758036 | 7.953369 | 6.711527 |
| H | 6.420376 | 6.853872 | 6.486797 |
| H | 9.054386 | 2.286935 | 7.348714 |
| H | 9.307985 | 3.106894 | 8.895968 |
| H | 7.906777 | 2.044958 | 8.67363 |
| H | 13.03747 | 6.700839 | 8.552993 |
| H | 12.0177 | 5.280191 | 8.832644 |
| H | 12.82259 | 5.500287 | 7.272393 |
| H | 6.497159 | 4.088776 | 9.518752 |
| H | 7.923293 | 5.12082 | 9.529962 |
| H | 6.509155 | 5.585454 | 8.57102 |
| H | 9.449643 | 7.983788 | 8.420112 |
| H | 10.01972 | 6.673692 | 9.467169 |
| H | 10.97302 | 8.147467 | 9.303652 |
| H | 5.567597 | 11.50502 | 1.579104 |
| H | 3.570547 | 2.110842 | 6.601564 |
| H | 0.982169 | 10.3195 | 5.206199 |
| H | 0.917044 | 11.62493 | 4.019404 |
| H | 0.840411 | 9.9278 | 3.494948 |
| H | 13.37236 | 7.73136 | 2.223432 |
| H | 12.41361 | 8.205819 | 0.809634 |
| H | 13.07558 | 9.438224 | 1.885778 |
| H | 3.963674 | 2.326715 | 4.17122 |
| H | 5.171243 | 5.288834 | 2.684133 |
| H | 5.264496 | 4.390705 | 1.161409 |
| H | 4.060188 | 3.95824 | 2.382832 |
| H | 6.269716 | 14.17874 | 0.267076 |
| H | 4.838692 | 13.19966 | -0.09626 |
| H | 5.033939 | 13.92132 | 1.506981 |
| H | 10.71809 | 10.44932 | 2.307351 |
| H | 10.13142 | 9.288388 | 1.107752 |
| H | 9.377866 | 9.380233 | 2.707538 |
| H | 1.484509 | 10.66009 | 1.201116 |
| H | 2.378092 | 10.91352 | -0.30551 |
| H | 1.232083 | 9.569024 | -0.16835 |
| H | 12.40087 | 9.121592 | 7.936729 |
| H | 7.490042 | 10.56743 | 0.251071 |
| H | 6.177148 | 11.17224 | -0.7729 |
| H | 7.653646 | 12.11499 | -0.58983 |
| H | 7.341151 | 14.37053 | 6.585564 |
| H | 7.680249 | 13.32672 | 7.978111 |
| H | 8.998895 | 13.88264 | 6.946414 |
| H | 9.762143 | 11.44226 | 6.459829 |
| H | 8.527401 | 10.95061 | 7.629072 |
| H | 8.561369 | 10.23696 | 6.008824 |
| H | 8.725719 | 1.772144 | 3.397569 |
| H | 9.996851 | 1.563975 | 4.604961 |
| H | 8.291647 | 1.654514 | 5.099637 |
| H | 0.864679 | 5.24497 | 1.793571 |
| H | 0.356204 | 6.508306 | 6.175908 |
| H | 0.921488 | 7.678337 | 7.370525 |
| H | 0.171127 | 8.236012 | 5.863384 |
| H | 8.397512 | 4.667472 | 7.178054 |
| H | 3.215318 | 8.041835 | -0.96025 |
| H | 4.317452 | 9.414239 | -0.92124 |
| H | 4.681962 | 7.966061 | 0.030365 |
| H | 6.748039 | 11.94628 | 6.189226 |
| H | 7.100749 | 3.678393 | 2.671342 |
| H | 5.585096 | 14.24816 | 3.804934 |
| H | 3.95777 | 14.30301 | 4.516134 |
| H | 5.370266 | 13.95965 | 5.523766 |
| H | 1.079122 | 5.526688 | 4.23974 |
| H | 10.41063 | 6.074136 | 7.110039 |
| H | 4.948567 | 1.53517 | 2.264673 |
| H | 6.197886 | 2.014743 | 1.113414 |
| H | 6.643548 | 1.196903 | 2.622531 |
| H | 2.679331 | 8.481628 | 5.864666 |
| H | 5.291796 | 2.888374 | 8.194486 |
| H | 13.10327 | 10.80201 | 6.264792 |
| H | 4.124786 | 6.42787 | 5.814695 |
| H | 3.211077 | 6.544578 | 7.326756 |
| H | 2.703192 | 5.420136 | 6.058977 |
| H | 12.58362 | 10.442 | 3.875085 |
| H | 2.742745 | 12.57298 | 4.77343 |
| H | 3.821856 | 9.866201 | 1.433178 |
| H | 10.88984 | 7.394676 | 2.54614 |
| H | 10.41055 | 13.91228 | 2.577462 |
| H | 8.735916 | 13.33266 | 0.854885 |
| H | 1.814789 | 6.948204 | 0.276511 |
| H | 11.15049 | 3.293847 | 3.927779 |
| H | 9.950277 | 13.40587 | 4.952158 |
| H | 12.79686 | 5.78758 | 3.239895 |
| H | 13.06034 | 5.958978 | 4.967055 |
| H | 12.98182 | 4.339851 | 4.238787 |
|  |  |  |  |
| 166 |  |  |  |
| Ts-third-insertion | |  |  |
| Yb | 8.287368 | 6.567813 | 3.794862 |
| Yb | 5.52959 | 9.638201 | 3.971911 |
| N | 3.221256 | 9.683598 | 4.614017 |
| N | 8.176802 | 4.196523 | 4.112857 |
| N | 5.47897 | 11.89801 | 4.79814 |
| N | 10.63018 | 6.195321 | 3.996676 |
| C | 8.71276 | 7.178519 | 0.438271 |
| C | 7.710924 | 7.794528 | 1.387003 |
| C | 7.750767 | 7.214191 | 6.467731 |
| C | 6.975117 | 8.066012 | 5.443209 |
| C | 12.35146 | 5.508731 | 5.628389 |
| C | 7.560252 | 13.1512 | 4.298328 |
| C | 6.697724 | 12.50426 | 5.219352 |
| C | 8.304777 | 12.92544 | 6.9908 |
| C | 10.24266 | 4.24007 | 5.407832 |
| C | 1.464889 | 6.391513 | 4.330958 |
| C | 8.775525 | 13.66837 | 4.760661 |
| C | 9.151833 | 13.56632 | 6.093322 |
| C | 12.08758 | 5.100603 | 1.72743 |
| C | 2.443589 | 7.783646 | 2.480563 |
| C | 2.189622 | 8.234181 | 6.307524 |
| C | 7.084087 | 12.38298 | 6.580133 |
| C | 3.089498 | 12.11015 | 4.3528 |
| C | 7.065618 | 3.037829 | 2.26421 |
| C | 13.22019 | 7.350266 | 1.580472 |
| C | 2.130626 | 7.561747 | 3.951814 |
| C | 7.047364 | 3.479932 | 3.612848 |
| C | 3.57412 | 8.552889 | 8.39288 |
| C | 6.532459 | 9.186558 | 0.512361 |
| C | 13.45893 | 8.650868 | 2.010146 |
| C | 2.497308 | 8.501083 | 4.947613 |
| C | 4.765898 | 2.68825 | 3.818918 |
| C | 12.30414 | 6.520884 | 2.232548 |
| C | 2.570546 | 9.195538 | 7.424782 |
| C | 4.376325 | 12.6241 | 4.646222 |
| C | 11.00644 | 5.330324 | 4.947796 |
| C | 11.61544 | 7.019789 | 3.371219 |
| C | 9.204283 | 1.902186 | 1.590404 |
| C | 11.1674 | 8.926241 | 5.034742 |
| C | 1.517654 | 7.053138 | 6.633655 |
| C | 4.415865 | 14.13734 | 4.775462 |
| C | 8.3255 | 3.151946 | 1.420657 |
| C | 6.166818 | 11.72753 | 7.60127 |
| C | 3.522102 | 6.806047 | 1.992597 |
| C | 5.798524 | 3.72855 | 5.856853 |
| C | 1.343032 | 9.709702 | 8.190844 |
| C | 1.149933 | 6.132641 | 5.658815 |
| C | 2.536466 | 10.81634 | 4.421522 |
| C | 8.842413 | 2.216408 | 5.4507 |
| C | 9.053985 | 3.627728 | 4.932477 |
| C | 6.915199 | 10.83987 | 8.601686 |
| C | 5.88514 | 3.292536 | 4.401682 |
| C | 5.329735 | 12.78355 | 8.339942 |
| C | 8.173621 | 12.51646 | 1.930411 |
| C | 12.77873 | 9.134329 | 3.118983 |
| C | 1.197532 | 7.689068 | 1.588598 |
| C | 5.617216 | 9.848298 | 1.449082 |
| C | 12.03383 | 5.00825 | 0.196645 |
| C | 11.86399 | 8.33952 | 3.81798 |
| C | 7.196756 | 14.78838 | 2.394306 |
| C | 8.050666 | 3.409967 | -0.06455 |
| C | 5.922883 | 2.434161 | 1.73407 |
| C | 13.16062 | 4.138969 | 2.263905 |
| C | 1.026231 | 10.75321 | 4.291352 |
| C | 4.773912 | 2.262573 | 2.497836 |
| C | 7.213989 | 13.31365 | 2.825278 |
| C | 10.18124 | 10.03129 | 4.63335 |
| C | 4.817288 | 4.894717 | 6.031667 |
| C | 12.15824 | 9.451203 | 6.083592 |
| C | 5.418386 | 2.568156 | 6.788798 |
| H | 8.130801 | 8.4382 | 2.170821 |
| H | 6.918588 | 7.126915 | 1.754723 |
| H | 9.394338 | 7.945931 | 0.056871 |
| H | 8.200435 | 6.733559 | -0.42154 |
| H | 9.346382 | 6.388468 | 0.870426 |
| H | 7.515423 | 6.142175 | 6.385364 |
| H | 7.55845 | 7.469595 | 7.519129 |
| H | 8.842772 | 7.301518 | 6.350644 |
| H | 5.90784 | 7.968359 | 5.726323 |
| H | 7.242755 | 9.118567 | 5.665726 |
| H | 12.27999 | 6.311071 | 6.372627 |
| H | 13.12748 | 5.805538 | 4.919789 |
| H | 12.66881 | 4.600605 | 6.143926 |
| H | 8.595124 | 12.85038 | 8.035121 |
| H | 10.73538 | 3.661873 | 6.183211 |
| H | 1.181187 | 5.673066 | 3.565514 |
| H | 9.436681 | 14.16877 | 4.057341 |
| H | 10.09652 | 13.9835 | 6.431576 |
| H | 11.12165 | 4.765341 | 2.121559 |
| H | 2.84345 | 8.797804 | 2.379115 |
| H | 2.354611 | 12.88724 | 4.17029 |
| H | 13.75577 | 6.971626 | 0.714385 |
| H | 3.150624 | 7.667398 | 8.880182 |
| H | 3.855935 | 9.259804 | 9.181244 |
| H | 4.485733 | 8.243377 | 7.872354 |
| H | 14.17087 | 9.280877 | 1.483938 |
| H | 3.869579 | 2.551477 | 4.418328 |
| H | 3.061092 | 10.05801 | 6.964082 |
| H | 9.486146 | 1.747494 | 2.635301 |
| H | 10.12472 | 1.990655 | 1.002434 |
| H | 8.670548 | 1.007501 | 1.250158 |
| H | 10.59923 | 8.115962 | 5.50493 |
| H | 1.274196 | 6.852018 | 7.674431 |
| H | 5.298261 | 14.48312 | 5.315773 |
| H | 3.520862 | 14.50408 | 5.284051 |
| H | 4.433244 | 14.59424 | 3.779234 |
| H | 8.900047 | 4.001375 | 1.810228 |
| H | 5.47054 | 11.09031 | 7.044379 |
| H | 4.442783 | 6.901365 | 2.579928 |
| H | 3.776765 | 6.996159 | 0.944721 |
| H | 3.187099 | 5.766919 | 2.081079 |
| H | 6.789635 | 4.079084 | 6.161716 |
| H | 0.62522 | 10.19794 | 7.524797 |
| H | 1.643693 | 10.43945 | 8.950958 |
| H | 0.818124 | 8.896091 | 8.703964 |
| H | 0.62145 | 5.22317 | 5.932276 |
| H | 8.060531 | 1.688713 | 4.903302 |
| H | 8.556974 | 2.245351 | 6.508431 |
| H | 9.771717 | 1.644405 | 5.384185 |
| H | 7.544651 | 10.10483 | 8.090864 |
| H | 6.202274 | 10.29634 | 9.229966 |
| H | 7.555643 | 11.42194 | 9.273721 |
| H | 5.976177 | 13.47068 | 8.898265 |
| H | 4.645879 | 12.30925 | 9.052915 |
| H | 4.731555 | 13.37825 | 7.644042 |
| H | 9.204176 | 12.87329 | 2.037467 |
| H | 7.8918 | 12.61824 | 0.876726 |
| H | 8.15913 | 11.45078 | 2.176366 |
| H | 12.96466 | 10.15041 | 3.458178 |
| H | 0.782084 | 6.675426 | 1.57747 |
| H | 1.448714 | 7.951558 | 0.555216 |
| H | 0.406347 | 8.365545 | 1.926234 |
| H | 11.32508 | 5.722748 | -0.23173 |
| H | 11.73006 | 4.000971 | -0.10815 |
| H | 13.01225 | 5.195136 | -0.25954 |
| H | 6.542191 | 15.39072 | 3.030473 |
| H | 6.845348 | 14.87918 | 1.360606 |
| H | 8.198099 | 15.23133 | 2.438993 |
| H | 7.626692 | 2.53058 | -0.56171 |
| H | 8.984355 | 3.649932 | -0.58295 |
| H | 7.357687 | 4.244068 | -0.21083 |
| H | 5.931688 | 2.090257 | 0.704074 |
| H | 14.15986 | 4.455435 | 1.94313 |
| H | 12.99162 | 3.124391 | 1.885845 |
| H | 13.15744 | 4.090885 | 3.355415 |
| H | 0.571438 | 10.33742 | 5.195448 |
| H | 0.738294 | 10.09056 | 3.469197 |
| H | 0.596972 | 11.7397 | 4.110264 |
| H | 3.893224 | 1.795192 | 2.065927 |
| H | 6.208173 | 12.90947 | 2.672661 |
| H | 10.70122 | 10.86683 | 4.153911 |
| H | 9.649161 | 10.43019 | 5.502651 |
| H | 9.435119 | 9.66617 | 3.918098 |
| H | 5.082296 | 5.745291 | 5.396528 |
| H | 4.805459 | 5.240846 | 7.07066 |
| H | 3.796162 | 4.603474 | 5.767934 |
| H | 12.88135 | 8.683455 | 6.375071 |
| H | 11.62397 | 9.77887 | 6.981875 |
| H | 12.72257 | 10.31142 | 5.707931 |
| H | 4.402224 | 2.210411 | 6.59108 |
| H | 5.450992 | 2.89406 | 7.834234 |
| H | 6.096336 | 1.717098 | 6.675764 |
| H | 5.743335 | 10.93959 | 1.416567 |
| H | 4.569362 | 9.630687 | 1.202512 |
| H | 7.331845 | 9.794908 | 0.083466 |
| H | 6.102415 | 8.534894 | -0.25229 |
|  |  |  |  |
| 166 |  |  |  |
| Product-third-insertion | | |  |
| Yb | 8.119311 | 6.77596 | 4.096637 |
| Yb | 5.827853 | 9.318534 | 4.481974 |
| N | 3.43931 | 9.427393 | 4.513673 |
| N | 8.038583 | 4.344989 | 4.21632 |
| N | 5.639135 | 11.71554 | 4.723742 |
| N | 10.49505 | 6.369249 | 4.130662 |
| C | 7.766819 | 7.49052 | -1.23814 |
| C | 7.15948 | 7.539219 | 0.162227 |
| C | 7.913714 | 7.061427 | 7.08893 |
| C | 7.13655 | 8.038629 | 6.190811 |
| C | 12.44399 | 5.318906 | 5.225239 |
| C | 7.754945 | 12.92009 | 4.282381 |
| C | 6.782002 | 12.43305 | 5.194107 |
| C | 8.105553 | 13.28515 | 7.039936 |
| C | 10.23503 | 4.233243 | 5.286251 |
| C | 1.641831 | 6.156027 | 4.234143 |
| C | 8.871379 | 13.59381 | 4.788772 |
| C | 9.054236 | 13.77978 | 6.153647 |
| C | 11.85717 | 5.59946 | 1.634683 |
| C | 2.810872 | 7.431617 | 2.40874 |
| C | 2.264339 | 8.065912 | 6.182424 |
| C | 6.965194 | 12.61427 | 6.58828 |
| C | 3.304216 | 11.81435 | 4.005778 |
| C | 6.78396 | 3.241928 | 2.424905 |
| C | 12.82541 | 7.918929 | 1.664357 |
| C | 2.371034 | 7.288783 | 3.858231 |
| C | 6.868179 | 3.653224 | 3.781988 |
| C | 3.274561 | 8.427912 | 8.480485 |
| C | 7.877707 | 8.502966 | 1.108308 |
| C | 13.04235 | 9.171276 | 2.22675 |
| C | 2.681108 | 8.261685 | 4.839751 |
| C | 4.613154 | 2.835787 | 4.144544 |
| C | 12.02831 | 6.960341 | 2.296088 |
| C | 2.576863 | 9.076988 | 7.277336 |
| C | 4.538995 | 12.38634 | 4.395663 |
| C | 10.97326 | 5.354155 | 4.853964 |
| C | 11.4119 | 7.284697 | 3.532603 |
| C | 8.956217 | 2.226025 | 1.643912 |
| C | 11.11179 | 8.916888 | 5.497106 |
| C | 1.522281 | 6.925695 | 6.502305 |
| C | 4.491289 | 13.90399 | 4.463511 |
| C | 7.971721 | 3.396372 | 1.485839 |
| C | 5.933041 | 12.14908 | 7.606115 |
| C | 3.927306 | 6.432614 | 2.074492 |
| C | 5.80143 | 3.82421 | 6.122393 |
| C | 1.317373 | 9.829039 | 7.733876 |
| C | 1.205104 | 5.973688 | 5.540272 |
| C | 2.768339 | 10.52018 | 4.126217 |
| C | 8.760882 | 2.27143 | 5.372344 |
| C | 8.977614 | 3.703029 | 4.90787 |
| C | 6.536368 | 11.22027 | 8.6685 |
| C | 5.770487 | 3.437237 | 4.651337 |
| C | 5.236399 | 13.34448 | 8.274491 |
| C | 8.864417 | 12.19525 | 2.110385 |
| C | 12.46805 | 9.469628 | 3.455251 |
| C | 1.648258 | 7.270856 | 1.417893 |
| C | 7.161931 | 8.678781 | 2.462258 |
| C | 11.07072 | 5.706062 | 0.321817 |
| C | 11.65945 | 8.547083 | 4.127052 |
| C | 7.254396 | 14.11617 | 2.105178 |
| C | 7.577923 | 3.542692 | 0.012288 |
| C | 5.608615 | 2.637702 | 1.972314 |
| C | 13.20633 | 4.907824 | 1.384887 |
| C | 1.29108 | 10.40567 | 3.799192 |
| C | 4.5233 | 2.436109 | 2.818632 |
| C | 7.607302 | 12.77632 | 2.772327 |
| C | 10.22386 | 10.16418 | 5.442598 |
| C | 4.842799 | 4.984221 | 6.418885 |
| C | 12.23656 | 9.114317 | 6.525411 |
| C | 5.487047 | 2.633551 | 7.041351 |
| H | 7.142075 | 6.531778 | 0.600389 |
| H | 6.107159 | 7.845222 | 0.084998 |
| H | 8.818906 | 7.187962 | -1.2116 |
| H | 7.722439 | 8.475141 | -1.71755 |
| H | 7.234784 | 6.783459 | -1.88332 |
| H | 7.346437 | 6.138086 | 7.245947 |
| H | 8.154555 | 7.453428 | 8.087536 |
| H | 8.885614 | 6.74835 | 6.668419 |
| H | 6.13211 | 8.140092 | 6.654497 |
| H | 7.595332 | 9.042202 | 6.325939 |
| H | 12.84626 | 6.31915 | 5.396329 |
| H | 13.02875 | 4.873764 | 4.412811 |
| H | 12.60507 | 4.710602 | 6.117772 |
| H | 8.243732 | 13.4352 | 8.107938 |
| H | 10.80944 | 3.555411 | 5.909562 |
| H | 1.403544 | 5.40658 | 3.483735 |
| H | 9.610964 | 13.98522 | 4.095017 |
| H | 9.928609 | 14.3085 | 6.523365 |
| H | 11.27784 | 4.966869 | 2.31382 |
| H | 3.213112 | 8.443687 | 2.283772 |
| H | 2.580734 | 12.55535 | 3.682218 |
| H | 13.28931 | 7.676012 | 0.711347 |
| H | 2.624468 | 7.705256 | 8.985882 |
| H | 3.549655 | 9.189479 | 9.218339 |
| H | 4.185615 | 7.901273 | 8.18098 |
| H | 13.66413 | 9.902563 | 1.717568 |
| H | 3.767968 | 2.675621 | 4.809015 |
| H | 3.262977 | 9.815666 | 6.849814 |
| H | 9.349787 | 2.161943 | 2.660779 |
| H | 9.806132 | 2.342939 | 0.962123 |
| H | 8.463752 | 1.275401 | 1.409395 |
| H | 10.50062 | 8.076452 | 5.84324 |
| H | 1.183683 | 6.783141 | 7.525662 |
| H | 5.465326 | 14.3543 | 4.651732 |
| H | 3.81072 | 14.21326 | 5.264095 |
| H | 4.08583 | 14.30896 | 3.531261 |
| H | 8.506434 | 4.306214 | 1.787745 |
| H | 5.167874 | 11.58426 | 7.062957 |
| H | 4.816497 | 6.598933 | 2.692613 |
| H | 4.233805 | 6.526044 | 1.027489 |
| H | 3.602762 | 5.40085 | 2.242121 |
| H | 6.816013 | 4.161665 | 6.356223 |
| H | 0.842258 | 10.36221 | 6.905453 |
| H | 1.567096 | 10.56535 | 8.506073 |
| H | 0.576824 | 9.140468 | 8.156534 |
| H | 0.623441 | 5.095394 | 5.807459 |
| H | 7.99155 | 1.760519 | 4.792008 |
| H | 8.446608 | 2.265448 | 6.422305 |
| H | 9.690238 | 1.700392 | 5.309573 |
| H | 6.997883 | 10.33745 | 8.21587 |
| H | 5.759396 | 10.8766 | 9.359934 |
| H | 7.300647 | 11.73084 | 9.264505 |
| H | 5.948327 | 13.94191 | 8.855243 |
| H | 4.452834 | 13.00167 | 8.9595 |
| H | 4.775801 | 14.00623 | 7.535505 |
| H | 9.711947 | 12.88624 | 2.176893 |
| H | 8.680371 | 12.01211 | 1.046546 |
| H | 9.172834 | 11.2511 | 2.566619 |
| H | 12.65708 | 10.43822 | 3.911787 |
| H | 1.258252 | 6.247358 | 1.416189 |
| H | 1.984916 | 7.491851 | 0.399214 |
| H | 0.814393 | 7.940065 | 1.651689 |
| H | 10.07513 | 6.126521 | 0.48851 |
| H | 10.94995 | 4.719412 | -0.13919 |
| H | 11.58636 | 6.34952 | -0.39992 |
| H | 6.333599 | 14.54666 | 2.505046 |
| H | 7.122912 | 13.98306 | 1.025469 |
| H | 8.056239 | 14.84825 | 2.254968 |
| H | 7.203271 | 2.60079 | -0.40432 |
| H | 8.451656 | 3.828261 | -0.58178 |
| H | 6.80588 | 4.304171 | -0.13298 |
| H | 5.539431 | 2.316812 | 0.937535 |
| H | 13.80363 | 5.447125 | 0.641433 |
| H | 13.05062 | 3.891843 | 1.005684 |
| H | 13.80553 | 4.839259 | 2.297913 |
| H | 0.735663 | 9.950384 | 4.623793 |
| H | 1.141543 | 9.755985 | 2.930872 |
| H | 0.853161 | 11.38011 | 3.578516 |
| H | 3.617636 | 1.965032 | 2.445995 |
| H | 6.779054 | 12.08359 | 2.585784 |
| H | 10.78655 | 11.04676 | 5.123225 |
| H | 9.797643 | 10.38937 | 6.425248 |
| H | 9.396325 | 10.03895 | 4.7369 |
| H | 5.086045 | 5.871949 | 5.826608 |
| H | 4.889567 | 5.263943 | 7.477348 |
| H | 3.80708 | 4.71634 | 6.190432 |
| H | 12.87072 | 8.226967 | 6.610201 |
| H | 11.81733 | 9.325296 | 7.51539 |
| H | 12.88087 | 9.956864 | 6.250957 |
| H | 4.449412 | 2.30139 | 6.927561 |
| H | 5.624411 | 2.916422 | 8.090908 |
| H | 6.13256 | 1.775558 | 6.832416 |
| H | 7.581539 | 9.623954 | 2.885302 |
| H | 6.118502 | 8.958375 | 2.177846 |
| H | 8.913548 | 8.15524 | 1.253877 |
| H | 7.983063 | 9.465032 | 0.580577 |
|  |  |  |  |
| 166 |  |  |  |
| Ts-first-SN1 | |  |  |
| Yb | 5.412911 | 9.771774 | 4.773185 |
| Yb | 8.092077 | 6.766014 | 5.568251 |
| N | 10.3807 | 6.45811 | 5.058756 |
| N | 5.795025 | 11.8704 | 3.772964 |
| N | 8.048032 | 4.463112 | 5.040509 |
| N | 3.255755 | 9.928566 | 3.833095 |
| C | 7.680219 | 2.394843 | 7.917977 |
| C | 12.49682 | 6.467242 | 7.892298 |
| C | 6.028328 | 4.070408 | 8.800558 |
| C | 11.14517 | 8.369828 | 8.825855 |
| C | 6.028952 | 12.28468 | 0.911692 |
| C | 4.102413 | 2.938436 | 5.123041 |
| C | 1.29432 | 11.19543 | 4.642935 |
| C | 11.87257 | 7.929032 | 1.440246 |
| C | 4.673825 | 3.324305 | 3.916668 |
| C | 5.930511 | 5.5658 | 2.030537 |
| C | 5.28231 | 13.54333 | 0.444518 |
| C | 11.55921 | 8.210811 | 6.310451 |
| C | 9.573796 | 8.751877 | 2.069184 |
| C | 1.039381 | 10.82126 | 0.564805 |
| C | 12.25183 | 9.425269 | 6.312711 |
| C | 6.532628 | 11.47523 | -0.28975 |
| C | 8.451934 | 13.65876 | 6.504121 |
| C | 7.575336 | 7.411247 | 9.468916 |
| C | 6.13367 | 3.577275 | 6.296378 |
| C | 8.828512 | 11.22151 | 5.984024 |
| C | 9.062884 | 3.66715 | 4.695688 |
| C | 8.803491 | 2.215698 | 4.340196 |
| C | 2.776946 | 11.05355 | 4.362108 |
| C | 0.693674 | 6.863809 | 2.437428 |
| C | 1.39668 | 7.473275 | 6.690127 |
| C | 6.907221 | 3.686103 | 7.605519 |
| C | 7.071187 | 7.941236 | 8.146083 |
| C | 3.083662 | 9.502192 | -0.10179 |
| C | 8.00325 | 12.47062 | 5.643159 |
| C | 6.547454 | 4.23987 | 2.50324 |
| C | 5.08536 | 14.13521 | 4.461338 |
| C | 1.16408 | 6.843018 | 3.744983 |
| C | 11.35039 | 7.452165 | 7.615718 |
| C | 6.369406 | 3.158877 | 1.42693 |
| C | 11.10365 | 7.688819 | 5.0711 |
| C | 11.01111 | 5.338654 | 4.698368 |
| C | 4.837198 | 12.65099 | 4.26906 |
| C | 2.500511 | 7.770207 | 5.666931 |
| C | 11.3058 | 8.416921 | 3.871532 |
| C | 4.829618 | 3.073474 | 6.300335 |
| C | 2.358074 | 8.920952 | 3.378251 |
| C | 12.47599 | 10.13209 | 5.136438 |
| C | 3.622991 | 6.727895 | 5.767534 |
| C | 6.72386 | 3.932355 | 5.054549 |
| C | 1.886608 | 8.946923 | 2.044843 |
| C | 11.9921 | 9.633453 | 3.933468 |
| C | 5.978832 | 3.821203 | 3.852973 |
| C | 3.553702 | 12.19966 | 4.677877 |
| C | 8.071899 | 12.74954 | 4.149602 |
| C | 1.990826 | 7.856604 | 4.237428 |
| C | 2.267773 | 10.05463 | 1.075547 |
| C | 10.78696 | 7.926856 | 2.526501 |
| C | 9.390006 | 13.49801 | 2.248208 |
| C | 8.341519 | 13.17371 | 1.395385 |
| C | 1.057545 | 7.912057 | 1.601628 |
| C | 10.40605 | 4.07245 | 4.584847 |
| C | 9.251696 | 13.27276 | 3.612149 |
| C | 7.000737 | 12.44297 | 3.276061 |
| C | 7.146924 | 12.63441 | 1.880837 |
| C | 12.48109 | 5.375036 | 4.327453 |
| C | 5.806927 | 9.262228 | 8.427071 |
| C | 4.612578 | 8.742505 | 9.04869 |
| C | 6.355467 | 10.4288 | 9.076748 |
| C | 3.953978 | 9.430889 | 10.0544 |
| C | 5.671775 | 11.0935 | 10.08021 |
| C | 4.446326 | 10.63193 | 10.57891 |
| H | 8.312753 | 6.603751 | 9.376189 |
| H | 6.730568 | 7.039409 | 10.05686 |
| H | 8.028959 | 8.226018 | 10.04139 |
| H | 7.834056 | 8.460697 | 7.545071 |
| H | 6.500062 | 7.204969 | 7.557558 |
| H | 8.411576 | 2.160981 | 7.141137 |
| H | 8.21852 | 2.489331 | 8.867515 |
| H | 6.991461 | 1.546837 | 8.003656 |
| H | 13.45187 | 7.000021 | 7.961517 |
| H | 12.33282 | 5.943901 | 8.840767 |
| H | 12.58398 | 5.714425 | 7.105861 |
| H | 5.347256 | 3.259834 | 9.080708 |
| H | 6.653704 | 4.276955 | 9.67429 |
| H | 5.424798 | 4.96052 | 8.599109 |
| H | 10.37625 | 9.126078 | 8.641567 |
| H | 10.83851 | 7.780956 | 9.695581 |
| H | 12.06869 | 8.889664 | 9.102788 |
| H | 5.315559 | 11.66022 | 1.4601 |
| H | 3.091227 | 2.541528 | 5.148126 |
| H | 0.848917 | 10.24977 | 4.958327 |
| H | 1.111568 | 11.95128 | 5.408983 |
| H | 0.771458 | 11.50999 | 3.732663 |
| H | 12.76978 | 7.388759 | 1.758087 |
| H | 11.49543 | 7.455195 | 0.527649 |
| H | 12.17707 | 8.946771 | 1.173639 |
| H | 4.095098 | 3.236088 | 3.001182 |
| H | 6.122063 | 6.377954 | 2.740473 |
| H | 6.35061 | 5.861204 | 1.062429 |
| H | 4.845084 | 5.475931 | 1.912794 |
| H | 5.956467 | 14.22612 | -0.08539 |
| H | 4.466064 | 13.28123 | -0.23804 |
| H | 4.850743 | 14.09017 | 1.288302 |
| H | 9.83427 | 9.80928 | 1.953359 |
| H | 9.204144 | 8.387285 | 1.10388 |
| H | 8.748745 | 8.69108 | 2.78735 |
| H | 0.445343 | 11.22908 | 1.388407 |
| H | 1.344854 | 11.65562 | -0.07624 |
| H | 0.380403 | 10.17549 | -0.02645 |
| H | 12.62203 | 9.827677 | 7.250676 |
| H | 7.075531 | 10.58108 | 0.032067 |
| H | 5.691262 | 11.15539 | -0.91278 |
| H | 7.202687 | 12.06074 | -0.9288 |
| H | 7.913969 | 14.57453 | 6.240274 |
| H | 8.267305 | 13.44839 | 7.562457 |
| H | 9.522759 | 13.86 | 6.392284 |
| H | 9.883387 | 11.36408 | 5.73149 |
| H | 8.760264 | 10.98808 | 7.051047 |
| H | 8.484081 | 10.34667 | 5.417638 |
| H | 8.519551 | 2.133243 | 3.285093 |
| H | 9.701935 | 1.612862 | 4.487284 |
| H | 7.986208 | 1.78753 | 4.923901 |
| H | 0.047616 | 6.069439 | 2.073598 |
| H | 0.989632 | 6.463244 | 6.571906 |
| H | 1.7983 | 7.553288 | 7.704755 |
| H | 0.567412 | 8.18129 | 6.598654 |
| H | 7.662282 | 4.476057 | 7.477325 |
| H | 2.504173 | 8.77724 | -0.68429 |
| H | 3.37698 | 10.3106 | -0.78057 |
| H | 3.992097 | 9.00005 | 0.244299 |
| H | 6.959329 | 12.264 | 5.906937 |
| H | 7.622481 | 4.404403 | 2.629114 |
| H | 5.647082 | 14.54824 | 3.620122 |
| H | 4.14875 | 14.68448 | 4.568165 |
| H | 5.685171 | 14.30899 | 5.361583 |
| H | 0.881345 | 6.024194 | 4.401187 |
| H | 10.44026 | 6.845414 | 7.499012 |
| H | 5.315417 | 3.016547 | 1.165052 |
| H | 6.893605 | 3.448921 | 0.510047 |
| H | 6.76218 | 2.189937 | 1.750778 |
| H | 2.916049 | 8.747987 | 5.942743 |
| H | 4.37331 | 2.781343 | 7.241215 |
| H | 13.01933 | 11.07285 | 5.160237 |
| H | 4.440618 | 6.933496 | 5.063713 |
| H | 4.038119 | 6.699484 | 6.77957 |
| H | 3.252999 | 5.727418 | 5.524215 |
| H | 12.15165 | 10.19985 | 3.019925 |
| H | 2.98305 | 12.96621 | 5.189976 |
| H | 2.904315 | 10.76124 | 1.616796 |
| H | 10.45093 | 6.892911 | 2.654029 |
| H | 10.3104 | 13.91816 | 1.851593 |
| H | 8.452808 | 13.34105 | 0.32733 |
| H | 0.690809 | 7.929974 | 0.578071 |
| H | 11.08768 | 3.27753 | 4.302956 |
| H | 10.07788 | 13.50792 | 4.277942 |
| H | 12.59139 | 5.61079 | 3.262867 |
| H | 13.03155 | 6.135862 | 4.883715 |
| H | 12.94765 | 4.402165 | 4.496329 |
| H | 6.878529 | 8.138301 | 4.391576 |
| H | 4.196721 | 7.801834 | 8.692076 |
| H | 7.309374 | 10.82484 | 8.733567 |
| H | 3.036202 | 9.0041 | 10.45828 |
| H | 6.119377 | 11.9926 | 10.50303 |
| H | 3.922822 | 11.16027 | 11.36896 |
| H | 5.659734 | 9.452662 | 7.068204 |
|  |  |  |  |
| 166 |  |  |  |
| Ts-second-SN1 | |  |  |
| Yb | 5.439031 | 9.801697 | 4.800098 |
| Yb | 8.091265 | 6.733048 | 5.585796 |
| N | 10.38137 | 6.448486 | 5.049729 |
| N | 5.753579 | 11.91286 | 3.775771 |
| N | 8.041256 | 4.442522 | 4.979492 |
| N | 3.256897 | 9.956029 | 3.879971 |
| C | 7.63534 | 2.146728 | 7.713236 |
| C | 12.52896 | 6.440395 | 7.856123 |
| C | 6.069976 | 3.837811 | 8.717237 |
| C | 11.1849 | 8.325473 | 8.831822 |
| C | 6.079297 | 12.26236 | 0.895721 |
| C | 4.068639 | 2.985842 | 4.998056 |
| C | 1.221471 | 11.24415 | 4.410818 |
| C | 11.83747 | 7.959934 | 1.436425 |
| C | 4.637823 | 3.438814 | 3.814641 |
| C | 5.863123 | 5.708556 | 2.002033 |
| C | 5.456073 | 13.49929 | 0.232697 |
| C | 11.55906 | 8.197952 | 6.308427 |
| C | 9.535226 | 8.756006 | 2.085247 |
| C | 1.086192 | 10.53095 | 0.441019 |
| C | 12.23349 | 9.422662 | 6.316432 |
| C | 6.618568 | 11.29513 | -0.16761 |
| C | 8.322114 | 13.73511 | 6.53391 |
| C | 7.67043 | 7.151521 | 9.277254 |
| C | 6.127626 | 3.491761 | 6.18818 |
| C | 8.759463 | 11.29704 | 6.068144 |
| C | 9.056969 | 3.664707 | 4.603009 |
| C | 8.806659 | 2.232038 | 4.170788 |
| C | 2.723472 | 11.12151 | 4.246619 |
| C | 0.84847 | 6.721255 | 2.5862 |
| C | 1.341922 | 7.755698 | 6.796692 |
| C | 6.91913 | 3.488236 | 7.490003 |
| C | 7.142745 | 7.838824 | 8.050261 |
| C | 3.260714 | 9.327753 | 0.019364 |
| C | 7.920515 | 12.52375 | 5.681584 |
| C | 6.521115 | 4.391842 | 2.442824 |
| C | 5.014399 | 14.22141 | 4.223646 |
| C | 1.287307 | 6.787362 | 3.902075 |
| C | 11.37425 | 7.423255 | 7.607621 |
| C | 6.390823 | 3.335527 | 1.335383 |
| C | 11.09754 | 7.681918 | 5.068896 |
| C | 11.00878 | 5.335485 | 4.663938 |
| C | 4.763375 | 12.72833 | 4.140159 |
| C | 2.515035 | 7.860777 | 5.811875 |
| C | 11.28105 | 8.424167 | 3.874617 |
| C | 4.81226 | 3.018777 | 6.172497 |
| C | 2.405284 | 8.894823 | 3.459828 |
| C | 12.43531 | 10.14531 | 5.145793 |
| C | 3.533443 | 6.740224 | 6.061309 |
| C | 6.712537 | 3.923337 | 4.968337 |
| C | 1.961219 | 8.833036 | 2.115584 |
| C | 11.94918 | 9.650344 | 3.942297 |
| C | 5.953946 | 3.908638 | 3.770478 |
| C | 3.450335 | 12.316 | 4.478152 |
| C | 8.02854 | 12.77939 | 4.186468 |
| C | 2.060546 | 7.858398 | 4.360783 |
| C | 2.324554 | 9.899725 | 1.093428 |
| C | 10.75856 | 7.939078 | 2.529047 |
| C | 9.395869 | 13.50005 | 2.30935 |
| C | 8.36599 | 13.16965 | 1.436838 |
| C | 1.188704 | 7.742406 | 1.707148 |
| C | 10.40228 | 4.073536 | 4.518024 |
| C | 9.223866 | 13.28985 | 3.671959 |
| C | 6.97485 | 12.46705 | 3.293512 |
| C | 7.157503 | 12.64077 | 1.899846 |
| C | 12.47841 | 5.378845 | 4.290734 |
| C | 5.780605 | 9.419211 | 8.716011 |
| C | 4.621889 | 8.894074 | 9.314033 |
| C | 6.433691 | 10.50051 | 9.33252 |
| C | 4.081502 | 9.504983 | 10.44245 |
| C | 5.876985 | 11.09712 | 10.46074 |
| C | 4.693231 | 10.61666 | 11.02555 |
| H | 8.340224 | 6.295755 | 9.095525 |
| H | 6.835144 | 6.781653 | 9.881651 |
| H | 8.222483 | 7.865285 | 9.897077 |
| H | 7.801353 | 8.513724 | 7.49832 |
| H | 6.375667 | 7.334913 | 7.457125 |
| H | 8.340702 | 1.924256 | 6.909305 |
| H | 8.194083 | 2.160785 | 8.655668 |
| H | 6.909253 | 1.327465 | 7.763494 |
| H | 13.48331 | 6.975787 | 7.914348 |
| H | 12.38278 | 5.907391 | 8.802212 |
| H | 12.60545 | 5.695173 | 7.061451 |
| H | 5.3601 | 3.040226 | 8.961209 |
| H | 6.712738 | 3.971255 | 9.592868 |
| H | 5.500176 | 4.760229 | 8.571412 |
| H | 10.41144 | 9.081802 | 8.668013 |
| H | 10.89431 | 7.723964 | 9.698505 |
| H | 12.11102 | 8.844866 | 9.101074 |
| H | 5.289889 | 11.74436 | 1.449936 |
| H | 3.048033 | 2.613018 | 5.00759 |
| H | 0.796513 | 10.35259 | 4.878177 |
| H | 0.959621 | 12.12048 | 5.00641 |
| H | 0.741782 | 11.34806 | 3.431521 |
| H | 12.74166 | 7.425332 | 1.74411 |
| H | 11.45916 | 7.49016 | 0.52219 |
| H | 12.1311 | 8.982642 | 1.176635 |
| H | 4.049077 | 3.426685 | 2.901246 |
| H | 6.018199 | 6.502687 | 2.740325 |
| H | 6.285232 | 6.048348 | 1.049443 |
| H | 4.783193 | 5.585811 | 1.865827 |
| H | 6.208046 | 14.07355 | -0.32043 |
| H | 4.673835 | 13.20517 | -0.4762 |
| H | 5.003966 | 14.16818 | 0.971136 |
| H | 9.785462 | 9.816405 | 1.974099 |
| H | 9.162319 | 8.394279 | 1.120101 |
| H | 8.716299 | 8.682896 | 2.809231 |
| H | 0.397378 | 10.93969 | 1.186705 |
| H | 1.38121 | 11.34626 | -0.22872 |
| H | 0.52816 | 9.801662 | -0.15657 |
| H | 12.60738 | 9.820692 | 7.254833 |
| H | 7.052824 | 10.40172 | 0.291514 |
| H | 5.815717 | 10.97596 | -0.83988 |
| H | 7.39421 | 11.76233 | -0.78418 |
| H | 7.762015 | 14.63193 | 6.25157 |
| H | 8.127437 | 13.53409 | 7.592482 |
| H | 9.388294 | 13.96611 | 6.4344 |
| H | 9.819861 | 11.45832 | 5.850994 |
| H | 8.658816 | 11.07507 | 7.135002 |
| H | 8.455129 | 10.40647 | 5.504024 |
| H | 8.680201 | 2.190936 | 3.082734 |
| H | 9.660567 | 1.598129 | 4.421336 |
| H | 7.904977 | 1.812236 | 4.619385 |
| H | 0.245625 | 5.882599 | 2.24846 |
| H | 0.825753 | 6.793477 | 6.709365 |
| H | 1.701086 | 7.848901 | 7.826638 |
| H | 0.6039 | 8.545411 | 6.625933 |
| H | 7.705101 | 4.250573 | 7.395697 |
| H | 2.77894 | 8.512486 | -0.53196 |
| H | 3.535734 | 10.10128 | -0.70593 |
| H | 4.179665 | 8.932399 | 0.46218 |
| H | 6.8721 | 12.30187 | 5.914951 |
| H | 7.588088 | 4.588918 | 2.588329 |
| H | 5.454269 | 14.59243 | 3.293696 |
| H | 4.094974 | 14.77355 | 4.422475 |
| H | 5.733495 | 14.4465 | 5.018357 |
| H | 1.023415 | 5.991391 | 4.593964 |
| H | 10.46476 | 6.815052 | 7.498207 |
| H | 5.343173 | 3.15724 | 1.069716 |
| H | 6.903253 | 3.672181 | 0.427688 |
| H | 6.823064 | 2.375615 | 1.633934 |
| H | 3.007109 | 8.820252 | 6.017381 |
| H | 4.360466 | 2.669286 | 7.096054 |
| H | 12.96414 | 11.09418 | 5.174146 |
| H | 4.401894 | 6.827233 | 5.396279 |
| H | 3.89075 | 6.760742 | 7.095512 |
| H | 3.095972 | 5.754685 | 5.875505 |
| H | 12.09115 | 10.2283 | 3.033055 |
| H | 2.833236 | 13.12334 | 4.855348 |
| H | 2.867005 | 10.69215 | 1.617708 |
| H | 10.43287 | 6.901173 | 2.650767 |
| H | 10.32749 | 13.91119 | 1.929815 |
| H | 8.502658 | 13.32173 | 0.368964 |
| H | 0.848322 | 7.692367 | 0.675552 |
| H | 11.08351 | 3.286521 | 4.212508 |
| H | 10.03527 | 13.52752 | 4.355068 |
| H | 12.58701 | 5.633331 | 3.230324 |
| H | 13.02965 | 6.130289 | 4.858879 |
| H | 12.94574 | 4.403317 | 4.441706 |
| H | 6.873538 | 8.129797 | 4.444396 |
| H | 4.130291 | 8.02459 | 8.88839 |
| H | 7.35821 | 10.89195 | 8.919376 |
| H | 3.172651 | 9.094251 | 10.87861 |
| H | 6.387116 | 11.94677 | 10.91108 |
| H | 4.267559 | 11.08684 | 11.9068 |
| H | 5.562303 | 9.697799 | 7.053207 |
|  |  |  |  |
| 157 |  |  |  |
| Ts-First | Insertion-Propene | |  |
| Yb | 5.768409 | 9.220634 | 3.525503 |
| Yb | 8.268079 | 6.523891 | 3.907441 |
| N | 10.59838 | 6.216176 | 4.252532 |
| N | 5.809707 | 11.52083 | 4.202015 |
| N | 8.376352 | 4.179142 | 4.229718 |
| N | 3.465769 | 9.399596 | 4.192275 |
| C | 6.216206 | 2.762894 | 7.460735 |
| C | 12.15555 | 8.218725 | 7.532799 |
| C | 5.803843 | 5.197364 | 6.954426 |
| C | 9.775056 | 8.839527 | 6.978431 |
| C | 7.890944 | 12.29133 | 2.256574 |
| C | 4.459378 | 2.780249 | 3.479194 |
| C | 1.319449 | 10.52906 | 3.717166 |
| C | 12.95658 | 6.593352 | 0.8853 |
| C | 5.398224 | 2.81937 | 2.45465 |
| C | 7.380185 | 4.362962 | 0.530772 |
| C | 8.185234 | 13.57016 | 1.459499 |
| C | 11.41787 | 8.376078 | 5.112576 |
| C | 10.558 | 7.29784 | 0.5557 |
| C | 5.602222 | 9.382027 | 0.959326 |
| C | 1.053724 | 7.425086 | 1.413413 |
| C | 11.96408 | 9.634802 | 4.845132 |
| C | 8.77711 | 11.14007 | 1.761711 |
| C | 5.700397 | 13.27945 | 7.8949 |
| C | 6.139642 | 3.558419 | 5.055387 |
| C | 6.551391 | 10.90599 | 7.993424 |
| C | 9.399613 | 3.395674 | 4.56879 |
| C | 9.163838 | 1.912551 | 4.757186 |
| C | 2.83046 | 10.52774 | 3.874373 |
| C | 1.329017 | 5.988019 | 5.51532 |
| C | 2.043357 | 10.1002 | 7.37603 |
| C | 6.509148 | 3.915541 | 6.488323 |
| C | 3.373893 | 6.470703 | 1.63112 |
| C | 6.107923 | 12.04942 | 7.070686 |
| C | 7.708516 | 3.250224 | 1.536616 |
| C | 4.886507 | 13.77914 | 3.88059 |
| C | 1.845418 | 6.903083 | 6.426602 |
| C | 11.00248 | 8.030493 | 6.535525 |
| C | 7.799097 | 1.893999 | 0.822915 |
| C | 11.27802 | 7.452123 | 4.046295 |
| C | 11.28454 | 5.124544 | 4.590622 |
| C | 4.745929 | 12.26943 | 3.909313 |
| C | 3.084745 | 9.022645 | 7.03403 |
| C | 11.71533 | 7.801231 | 2.742676 |
| C | 4.835878 | 3.144717 | 4.766242 |
| C | 2.715114 | 8.263455 | 4.615804 |
| C | 12.38316 | 9.987634 | 3.567983 |
| C | 6.539393 | 8.346803 | 0.877636 |
| C | 3.579596 | 8.346864 | 8.317358 |
| C | 7.085908 | 3.619549 | 4.001152 |
| C | 2.198561 | 7.329819 | 3.684711 |
| C | 12.26184 | 9.070095 | 2.530737 |
| C | 6.71307 | 3.231509 | 2.688512 |
| C | 3.445469 | 11.7814 | 3.649167 |
| C | 7.187061 | 12.3766 | 6.049581 |
| C | 2.546398 | 8.036785 | 6.00719 |
| C | 2.379007 | 7.501005 | 2.184701 |
| C | 11.60582 | 6.829362 | 1.575525 |
| C | 9.427325 | 13.22036 | 5.614312 |
| C | 9.237623 | 13.01003 | 4.254462 |
| C | 1.515867 | 6.204142 | 4.157098 |
| C | 10.72082 | 3.844759 | 4.757451 |
| C | 8.400726 | 12.90647 | 6.497036 |
| C | 7.00278 | 12.15257 | 4.66209 |
| C | 8.039952 | 12.48833 | 3.756724 |
| C | 12.77874 | 5.226933 | 4.810814 |
| H | 7.093349 | 7.774319 | 2.224248 |
| H | 7.54561 | 8.576003 | 0.53442 |
| H | 4.544867 | 9.170986 | 0.828045 |
| H | 5.911386 | 10.41223 | 0.807472 |
| H | 6.940479 | 7.955209 | 4.91431 |
| H | 6.697811 | 1.831435 | 7.146504 |
| H | 6.580854 | 3.010529 | 8.463543 |
| H | 5.141423 | 2.56783 | 7.542242 |
| H | 12.43739 | 9.272841 | 7.629488 |
| H | 11.85878 | 7.86725 | 8.526902 |
| H | 13.05004 | 7.664447 | 7.230955 |
| H | 4.714935 | 5.084303 | 6.921074 |
| H | 6.087089 | 5.435934 | 7.985978 |
| H | 6.058542 | 6.060926 | 6.32923 |
| H | 8.909577 | 8.664854 | 6.32896 |
| H | 9.484721 | 8.570377 | 8.00027 |
| H | 9.979495 | 9.915259 | 6.958788 |
| H | 6.847766 | 12.0194 | 2.064816 |
| H | 3.440625 | 2.461618 | 3.276798 |
| H | 0.910114 | 11.53661 | 3.810107 |
| H | 1.048033 | 10.14742 | 2.726116 |
| H | 0.835843 | 9.880899 | 4.451239 |
| H | 13.71915 | 6.260628 | 1.596161 |
| H | 12.86062 | 5.825982 | 0.109437 |
| H | 13.32934 | 7.503184 | 0.402536 |
| H | 5.104801 | 2.522312 | 1.450717 |
| H | 7.367398 | 5.350349 | 1.007948 |
| H | 8.118487 | 4.391604 | -0.27813 |
| H | 6.393218 | 4.21045 | 0.080203 |
| H | 9.234108 | 13.87308 | 1.551053 |
| H | 7.98198 | 13.41104 | 0.394757 |
| H | 7.569368 | 14.40872 | 1.799063 |
| H | 10.83517 | 8.26348 | 0.118692 |
| H | 10.45526 | 6.575759 | -0.26185 |
| H | 9.57171 | 7.424421 | 1.018031 |
| H | 0.314362 | 8.127792 | 1.809164 |
| H | 1.213652 | 7.65901 | 0.355089 |
| H | 0.614168 | 6.422865 | 1.462405 |
| H | 12.06031 | 10.35294 | 5.654999 |
| H | 8.554928 | 10.20265 | 2.285024 |
| H | 8.63562 | 10.96978 | 0.688559 |
| H | 9.836591 | 11.35455 | 1.935731 |
| H | 5.371744 | 14.10534 | 7.256239 |
| H | 4.875985 | 13.02931 | 8.571824 |
| H | 6.529511 | 13.64756 | 8.509457 |
| H | 7.459476 | 11.17172 | 8.546385 |
| H | 5.772078 | 10.67887 | 8.72876 |
| H | 6.760015 | 9.994917 | 7.424584 |
| H | 10.07311 | 1.39708 | 5.070146 |
| H | 8.384846 | 1.734565 | 5.504873 |
| H | 8.809195 | 1.456689 | 3.826819 |
| H | 0.78551 | 5.113757 | 5.863849 |
| H | 1.134304 | 9.645634 | 7.786884 |
| H | 2.439215 | 10.79611 | 8.124632 |
| H | 1.761771 | 10.68434 | 6.49656 |
| H | 7.588669 | 4.099495 | 6.514068 |
| H | 3.003059 | 5.450196 | 1.773728 |
| H | 3.540971 | 6.624866 | 0.559076 |
| H | 4.343451 | 6.529987 | 2.137455 |
| H | 5.224945 | 11.70933 | 6.521471 |
| H | 8.695919 | 3.466223 | 1.9577 |
| H | 5.733502 | 14.08651 | 3.260533 |
| H | 3.981271 | 14.25959 | 3.506114 |
| H | 5.085408 | 14.16159 | 4.887255 |
| H | 1.700075 | 6.731404 | 7.489114 |
| H | 10.72705 | 6.970449 | 6.549482 |
| H | 6.861526 | 1.637173 | 0.318145 |
| H | 8.58608 | 1.915372 | 0.061207 |
| H | 8.02898 | 1.085195 | 1.523403 |
| H | 3.938418 | 9.528833 | 6.568543 |
| H | 4.10115 | 3.110002 | 5.56628 |
| H | 12.80772 | 10.97056 | 3.383306 |
| H | 4.297253 | 7.550132 | 8.102796 |
| H | 4.072207 | 9.08185 | 8.961401 |
| H | 2.757556 | 7.916222 | 8.900322 |
| H | 12.59942 | 9.343268 | 1.533842 |
| H | 2.746401 | 12.55438 | 3.35002 |
| H | 2.801747 | 8.496962 | 2.017328 |
| H | 11.26973 | 5.868377 | 1.97836 |
| H | 10.36321 | 13.63243 | 5.982425 |
| H | 10.03522 | 13.25944 | 3.559025 |
| H | 1.118586 | 5.487392 | 3.442622 |
| H | 11.42495 | 3.07253 | 5.044456 |
| H | 8.542337 | 13.07668 | 7.561807 |
| H | 13.21427 | 4.258784 | 5.062307 |
| H | 13.27868 | 5.612728 | 3.916789 |
| H | 13.00375 | 5.930396 | 5.61882 |
| C | 6.05003 | 7.09832 | 0.120395 |
| H | 6.672717 | 6.894983 | -0.75825 |
| H | 6.078795 | 6.207794 | 0.758553 |
| H | 5.018191 | 7.221721 | -0.22773 |
|  |  |  |  |
| 157 |  |  |  |
| Product-First | Insertion-Propene | |  |
| Yb | 8.078995 | 6.641005 | 4.286092 |
| Yb | 5.805075 | 9.244204 | 3.995392 |
| N | 3.45136 | 9.601626 | 3.803214 |
| N | 8.123138 | 4.262185 | 4.649307 |
| N | 5.737803 | 11.63688 | 4.0204 |
| N | 10.44709 | 6.390324 | 4.524546 |
| C | 12.61666 | 5.251752 | 4.286285 |
| C | 7.513314 | 12.46643 | 2.547461 |
| C | 7.03629 | 12.19959 | 3.858146 |
| C | 9.19062 | 12.8506 | 4.757888 |
| C | 10.50981 | 3.966544 | 4.23064 |
| C | 1.933681 | 6.901767 | 1.732904 |
| C | 8.821638 | 12.93145 | 2.387613 |
| C | 9.662497 | 13.11831 | 3.479046 |
| C | 11.33793 | 8.204282 | 2.353073 |
| C | 2.835003 | 9.13063 | 0.975193 |
| C | 2.389004 | 7.467505 | 4.438517 |
| C | 7.884422 | 12.40145 | 4.976089 |
| C | 3.351204 | 11.97107 | 4.342734 |
| C | 5.962766 | 3.199349 | 4.076994 |
| C | 12.25033 | 9.651083 | 4.191707 |
| C | 2.51629 | 8.125252 | 2.074785 |
| C | 6.882888 | 3.676637 | 5.04587 |
| C | 3.65463 | 6.839861 | 6.540243 |
| C | 7.669082 | 6.741113 | 1.406616 |
| C | 12.5237 | 9.951606 | 5.519487 |
| C | 2.755031 | 8.410504 | 3.445385 |
| C | 5.292265 | 3.1296 | 6.795041 |
| C | 11.59281 | 8.47132 | 3.82923 |
| C | 2.579724 | 7.745036 | 5.923128 |
| C | 4.687384 | 12.41603 | 4.270855 |
| C | 11.10164 | 5.240203 | 4.338533 |
| C | 11.19356 | 7.565224 | 4.843564 |
| C | 6.360302 | 1.757339 | 2.029707 |
| C | 11.08137 | 6.931079 | 7.336845 |
| C | 1.821227 | 6.253009 | 4.040603 |
| C | 4.880102 | 13.89826 | 4.530053 |
| C | 6.277547 | 3.18676 | 2.58764 |
| C | 7.41297 | 12.16634 | 6.404472 |
| C | 3.729713 | 8.540646 | -0.12346 |
| C | 7.494869 | 4.110314 | 7.507358 |
| C | 1.26352 | 7.615297 | 6.704705 |
| C | 1.59263 | 5.963971 | 2.70112 |
| C | 2.771832 | 10.71239 | 4.091818 |
| C | 9.113474 | 1.990019 | 4.645954 |
| C | 9.200882 | 3.498325 | 4.491889 |
| C | 8.143089 | 10.98561 | 7.05972 |
| C | 6.538512 | 3.640976 | 6.420671 |
| C | 7.558376 | 13.42979 | 7.266724 |
| C | 7.291714 | 11.56475 | 0.17261 |
| C | 12.13514 | 9.058313 | 6.510537 |
| C | 1.551163 | 9.707218 | 0.357185 |
| C | 6.825418 | 7.709236 | 2.252356 |
| C | 10.37816 | 9.245099 | 1.759387 |
| C | 11.47466 | 7.86595 | 6.20171 |
| C | 6.100627 | 13.67843 | 0.844337 |
| C | 5.258227 | 4.006606 | 1.784503 |
| C | 4.729681 | 2.69858 | 4.505444 |
| C | 12.63807 | 8.162248 | 1.53534 |
| C | 1.259663 | 10.66359 | 4.186576 |
| C | 4.387621 | 2.659037 | 5.851419 |
| C | 6.614268 | 12.31277 | 1.327808 |
| C | 10.02416 | 7.573368 | 8.246335 |
| C | 6.889572 | 5.240124 | 8.35221 |
| C | 12.29567 | 6.48357 | 8.164054 |
| C | 7.938626 | 2.942969 | 8.402149 |
| H | 13.01346 | 4.283206 | 3.97819 |
| H | 13.02996 | 5.493044 | 5.271294 |
| H | 12.98475 | 6.01952 | 3.601078 |
| H | 9.848353 | 12.99875 | 5.61062 |
| H | 11.22335 | 3.166649 | 4.062123 |
| H | 1.741331 | 6.680545 | 0.685864 |
| H | 9.187701 | 13.15384 | 1.388676 |
| H | 10.67819 | 13.47598 | 3.333606 |
| H | 10.8667 | 7.217971 | 2.273794 |
| H | 3.378603 | 9.960251 | 1.439671 |
| H | 2.636785 | 12.75157 | 4.581428 |
| H | 12.55603 | 10.34638 | 3.413712 |
| H | 3.398688 | 5.781056 | 6.429254 |
| H | 3.76633 | 7.049592 | 7.609994 |
| H | 4.634933 | 6.991296 | 6.074456 |
| H | 13.03865 | 10.87232 | 5.780114 |
| H | 5.03082 | 3.094188 | 7.849838 |
| H | 2.920793 | 8.781424 | 6.022804 |
| H | 7.107701 | 1.156752 | 2.555503 |
| H | 6.630131 | 1.776552 | 0.967904 |
| H | 5.39758 | 1.241541 | 2.1173 |
| H | 10.63801 | 6.035565 | 6.89025 |
| H | 1.548741 | 5.522388 | 4.798038 |
| H | 5.015574 | 14.07158 | 5.604165 |
| H | 4.004104 | 14.46908 | 4.214187 |
| H | 5.764384 | 14.29144 | 4.025059 |
| H | 7.262641 | 3.646696 | 2.455242 |
| H | 6.347382 | 11.91597 | 6.363756 |
| H | 4.666273 | 8.150651 | 0.285739 |
| H | 3.976936 | 9.309216 | -0.8643 |
| H | 3.231469 | 7.723517 | -0.65689 |
| H | 8.388867 | 4.503847 | 7.012228 |
| H | 0.469091 | 8.228549 | 6.268262 |
| H | 1.405122 | 7.92998 | 7.744464 |
| H | 0.907125 | 6.57961 | 6.722801 |
| H | 1.145084 | 5.016615 | 2.413049 |
| H | 8.087356 | 1.623518 | 4.623717 |
| H | 9.557101 | 1.690385 | 5.602162 |
| H | 9.687733 | 1.493927 | 3.858299 |
| H | 7.965216 | 10.0487 | 6.521033 |
| H | 7.797671 | 10.84543 | 8.090361 |
| H | 9.225154 | 11.15295 | 7.087421 |
| H | 8.611014 | 13.69323 | 7.417155 |
| H | 7.116992 | 13.26904 | 8.256437 |
| H | 7.065168 | 14.29286 | 6.809229 |
| H | 8.124615 | 12.13656 | -0.25075 |
| H | 6.57512 | 11.39053 | -0.63722 |
| H | 7.684324 | 10.5947 | 0.491772 |
| H | 12.35248 | 9.289271 | 7.550707 |
| H | 0.959257 | 8.921653 | -0.12609 |
| H | 1.794243 | 10.45751 | -0.40357 |
| H | 0.917037 | 10.18532 | 1.109277 |
| H | 9.417457 | 9.256687 | 2.284758 |
| H | 10.18421 | 9.038023 | 0.700746 |
| H | 10.79459 | 10.25488 | 1.830622 |
| H | 5.549368 | 14.20381 | 1.628998 |
| H | 5.430863 | 13.55803 | -0.01461 |
| H | 6.933453 | 14.31997 | 0.534053 |
| H | 4.258639 | 3.562303 | 1.837033 |
| H | 5.545211 | 4.048777 | 0.728112 |
| H | 5.175924 | 5.031675 | 2.155952 |
| H | 4.025277 | 2.325844 | 3.765994 |
| H | 13.13961 | 9.136159 | 1.532725 |
| H | 12.42572 | 7.898268 | 0.493439 |
| H | 13.34682 | 7.42803 | 1.930067 |
| H | 0.826931 | 10.03613 | 3.404106 |
| H | 0.823535 | 11.66211 | 4.123325 |
| H | 0.958365 | 10.22488 | 5.144859 |
| H | 3.426225 | 2.259301 | 6.162608 |
| H | 5.740772 | 11.7296 | 1.641042 |
| H | 10.40509 | 8.489994 | 8.710245 |
| H | 9.740688 | 6.887978 | 9.05256 |
| H | 9.118768 | 7.836682 | 7.690381 |
| H | 6.635531 | 6.109978 | 7.738015 |
| H | 7.596672 | 5.565617 | 9.12249 |
| H | 5.97644 | 4.913128 | 8.86166 |
| H | 13.06461 | 6.019025 | 7.539077 |
| H | 11.99307 | 5.753521 | 8.922916 |
| H | 12.76049 | 7.327072 | 8.686403 |
| H | 7.091636 | 2.522848 | 8.956091 |
| H | 8.680668 | 3.278716 | 9.135011 |
| H | 8.383312 | 2.133394 | 7.81563 |
| H | 6.851687 | 8.684724 | 1.723522 |
| H | 5.767191 | 7.383614 | 2.160982 |
| H | 8.681817 | 7.128486 | 1.244946 |
| H | 7.792195 | 5.745677 | 1.872959 |
| H | 6.8839 | 8.041513 | 5.536836 |
| C | 7.097051 | 6.429522 | 0.011162 |
| H | 7.893941 | 6.340065 | -0.73588 |
| H | 6.536982 | 5.487487 | 0.010743 |
| H | 6.415583 | 7.218812 | -0.32608 |
|  |  |  |  |
| 157 |  |  |  |
| Ts-first-Allyl | |  |  |
| Yb | 5.880734 | 9.306085 | 3.444745 |
| Yb | 8.298226 | 6.655376 | 3.971978 |
| N | 10.65832 | 6.31822 | 4.115013 |
| N | 5.963798 | 11.46797 | 4.444967 |
| N | 8.3844 | 4.319232 | 4.405287 |
| N | 3.540148 | 9.430719 | 3.979644 |
| C | 6.481961 | 3.167864 | 7.863387 |
| C | 12.5639 | 8.345279 | 7.221299 |
| C | 5.951915 | 5.546063 | 7.218335 |
| C | 10.16682 | 8.992604 | 6.784508 |
| C | 8.180095 | 12.35765 | 2.745887 |
| C | 4.435159 | 2.856818 | 4.032379 |
| C | 1.473279 | 10.75981 | 3.643254 |
| C | 12.64697 | 6.452468 | 0.551165 |
| C | 5.303497 | 2.817874 | 2.946814 |
| C | 7.123141 | 4.222677 | 0.772197 |
| C | 8.707624 | 13.6053 | 2.026599 |
| C | 11.67874 | 8.439722 | 4.839822 |
| C | 10.32771 | 7.42561 | 0.367552 |
| C | 5.650031 | 11.28739 | 0.539383 |
| C | 2.011309 | 8.008006 | 1.056112 |
| C | 12.29519 | 9.646666 | 4.498604 |
| C | 8.943701 | 11.11039 | 2.278338 |
| C | 5.492844 | 12.70106 | 8.22102 |
| C | 6.206074 | 3.778591 | 5.420531 |
| C | 6.639758 | 10.44919 | 8.277028 |
| C | 9.424475 | 3.540122 | 4.700927 |
| C | 9.190552 | 2.074172 | 4.999436 |
| C | 2.981138 | 10.62471 | 3.778178 |
| C | 1.149468 | 6.108502 | 5.092925 |
| C | 1.120551 | 9.847003 | 7.32967 |
| C | 6.66463 | 4.254229 | 6.792301 |
| C | 3.415402 | 5.956797 | 1.442333 |
| C | 6.117815 | 11.57966 | 7.378614 |
| C | 7.536891 | 3.19604 | 1.837227 |
| C | 5.13542 | 13.7787 | 4.253546 |
| C | 1.393187 | 7.134262 | 5.995703 |
| C | 11.35323 | 8.147758 | 6.297922 |
| C | 7.597817 | 1.788778 | 1.225672 |
| C | 11.36891 | 7.515471 | 3.809624 |
| C | 11.34056 | 5.223014 | 4.45163 |
| C | 4.950983 | 12.27741 | 4.13437 |
| C | 2.420342 | 9.303659 | 6.71726 |
| C | 11.68834 | 7.819646 | 2.462003 |
| C | 4.891116 | 3.332947 | 5.25536 |
| C | 2.711155 | 8.328607 | 4.344045 |
| C | 12.61663 | 9.949123 | 3.179878 |
| C | 5.223481 | 10.01379 | 0.592908 |
| C | 3.333195 | 8.746287 | 7.818132 |
| C | 7.080607 | 3.754061 | 4.304923 |
| C | 2.480728 | 7.27293 | 3.424628 |
| C | 12.3114 | 9.037956 | 2.175318 |
| C | 6.625365 | 3.258567 | 3.055558 |
| C | 3.66779 | 11.86185 | 3.70537 |
| C | 7.219744 | 12.0948 | 6.466154 |
| C | 2.165861 | 8.247447 | 5.651163 |
| C | 3.012048 | 7.324689 | 2.000675 |
| C | 11.38298 | 6.853199 | 1.325888 |
| C | 9.432985 | 13.08809 | 6.269834 |
| C | 9.351445 | 12.98781 | 4.886153 |
| C | 1.697398 | 6.184723 | 3.818824 |
| C | 10.7641 | 3.971391 | 4.744467 |
| C | 8.371692 | 12.6375 | 7.044341 |
| C | 7.127586 | 12.02799 | 5.055094 |
| C | 8.214957 | 12.46902 | 4.260424 |
| C | 12.85192 | 5.285819 | 4.530681 |
| C | 5.99919 | 8.809784 | 0.220314 |
| H | 7.152564 | 7.698358 | 2.304655 |
| H | 4.20697 | 9.850275 | 0.958464 |
| H | 5.018622 | 12.10552 | 0.870534 |
| H | 6.627384 | 11.54139 | 0.135524 |
| H | 7.019673 | 8.15273 | 4.984334 |
| H | 6.978581 | 2.232894 | 7.585258 |
| H | 6.900888 | 3.501265 | 8.819098 |
| H | 5.422911 | 2.942125 | 8.029903 |
| H | 12.86158 | 9.397617 | 7.282387 |
| H | 12.32107 | 8.01749 | 8.237843 |
| H | 13.43383 | 7.774618 | 6.880587 |
| H | 4.868128 | 5.39855 | 7.27743 |
| H | 6.2999 | 5.868334 | 8.206328 |
| H | 6.133737 | 6.368093 | 6.51652 |
| H | 9.261066 | 8.817779 | 6.191985 |
| H | 9.930396 | 8.759488 | 7.828827 |
| H | 10.38739 | 10.0631 | 6.719906 |
| H | 7.132603 | 12.23609 | 2.450953 |
| H | 3.409532 | 2.514637 | 3.926367 |
| H | 1.040335 | 11.13265 | 4.577453 |
| H | 1.230706 | 11.48966 | 2.865569 |
| H | 0.988629 | 9.811389 | 3.408042 |
| H | 13.40851 | 6.026203 | 1.211437 |
| H | 12.4057 | 5.70439 | -0.21205 |
| H | 13.09553 | 7.311182 | 0.039487 |
| H | 4.947284 | 2.438096 | 1.992311 |
| H | 7.115977 | 5.244715 | 1.168257 |
| H | 7.81148 | 4.196022 | -0.08002 |
| H | 6.115888 | 4.014 | 0.394749 |
| H | 9.784908 | 13.74019 | 2.171522 |
| H | 8.537386 | 13.52171 | 0.947551 |
| H | 8.208513 | 14.51383 | 2.378227 |
| H | 10.6861 | 8.343802 | -0.11135 |
| H | 10.09477 | 6.705822 | -0.42505 |
| H | 9.393736 | 7.668721 | 0.886702 |
| H | 1.780017 | 9.027138 | 1.377231 |
| H | 2.40631 | 8.054927 | 0.034934 |
| H | 1.071219 | 7.445728 | 1.027741 |
| H | 12.52976 | 10.36261 | 5.281873 |
| H | 8.553754 | 10.19067 | 2.735085 |
| H | 8.88418 | 10.99049 | 1.19117 |
| H | 10.00008 | 11.16462 | 2.558889 |
| H | 5.086538 | 13.50125 | 7.594279 |
| H | 4.674352 | 12.30994 | 8.835767 |
| H | 6.227052 | 13.1518 | 8.898572 |
| H | 7.451907 | 10.79449 | 8.926672 |
| H | 5.841978 | 10.06785 | 8.92235 |
| H | 7.018618 | 9.617989 | 7.675343 |
| H | 10.11494 | 1.570999 | 5.28709 |
| H | 8.459301 | 1.951417 | 5.804013 |
| H | 8.776116 | 1.562737 | 4.124521 |
| H | 0.541536 | 5.254726 | 5.379782 |
| H | 0.598636 | 9.081601 | 7.914491 |
| H | 1.338197 | 10.68056 | 8.006292 |
| H | 0.425359 | 10.20406 | 6.563945 |
| H | 7.736581 | 4.469575 | 6.723367 |
| H | 2.541118 | 5.327602 | 1.242153 |
| H | 3.941797 | 6.082174 | 0.490768 |
| H | 4.071235 | 5.41332 | 2.12764 |
| H | 5.332986 | 11.16111 | 6.741277 |
| H | 8.548447 | 3.452853 | 2.167777 |
| H | 5.989763 | 14.11705 | 3.658539 |
| H | 4.245237 | 14.32098 | 3.931389 |
| H | 5.35424 | 14.05656 | 5.289241 |
| H | 0.976618 | 7.070948 | 6.998024 |
| H | 11.05986 | 7.094801 | 6.364283 |
| H | 6.632024 | 1.485589 | 0.806787 |
| H | 8.33056 | 1.75897 | 0.411808 |
| H | 7.886374 | 1.03686 | 1.966825 |
| H | 2.944999 | 10.14226 | 6.250601 |
| H | 4.211781 | 3.358632 | 6.103461 |
| H | 13.10484 | 10.8892 | 2.937028 |
| H | 4.296566 | 8.427952 | 7.41066 |
| H | 3.519614 | 9.505498 | 8.585674 |
| H | 2.873591 | 7.881898 | 8.310471 |
| H | 12.5655 | 9.274718 | 1.144901 |
| H | 3.017096 | 12.68828 | 3.439234 |
| H | 3.916498 | 7.9465 | 2.011492 |
| H | 10.97031 | 5.942348 | 1.772642 |
| H | 10.31741 | 13.51007 | 6.739785 |
| H | 10.18529 | 13.32627 | 4.277204 |
| H | 1.511615 | 5.380919 | 3.113032 |
| H | 11.47643 | 3.199939 | 5.012701 |
| H | 8.436804 | 12.70436 | 8.127671 |
| H | 13.28089 | 4.313875 | 4.778757 |
| H | 13.27735 | 5.623575 | 3.580614 |
| H | 13.17227 | 6.010005 | 5.286442 |
| H | 6.555573 | 8.230953 | 1.228698 |
| H | 5.385234 | 8.04113 | -0.25748 |
| H | 6.862423 | 9.045156 | -0.40964 |
|  |  |  |  |
| 155 |  |  |  |
| Product-first-allyl | |  |  |
| Yb | 5.792798 | 9.403587 | 3.698532 |
| Yb | 8.260601 | 6.676783 | 3.98273 |
| N | 10.60266 | 6.260627 | 4.132233 |
| N | 5.8615 | 11.69141 | 4.402169 |
| N | 8.267179 | 4.308048 | 4.21956 |
| N | 3.445779 | 9.611245 | 4.186943 |
| C | 6.055835 | 2.833496 | 7.410299 |
| C | 12.4584 | 8.386065 | 7.17247 |
| C | 5.66278 | 5.280342 | 6.94541 |
| C | 10.09603 | 9.126356 | 6.694243 |
| C | 7.929028 | 12.76543 | 2.543915 |
| C | 4.348139 | 2.941313 | 3.412941 |
| C | 1.329718 | 10.89591 | 4.014608 |
| C | 12.80528 | 5.832164 | 1.097003 |
| C | 5.285772 | 3.038563 | 2.390647 |
| C | 7.025845 | 4.148622 | 0.220325 |
| C | 8.071259 | 14.15928 | 1.913979 |
| C | 11.60093 | 8.419044 | 4.78753 |
| C | 10.94225 | 7.171893 | 0.067206 |
| C | 5.880644 | 9.659548 | 1.030621 |
| C | 1.389546 | 8.32251 | 1.285738 |
| C | 12.21495 | 9.614406 | 4.400575 |
| C | 8.95217 | 11.80697 | 1.916855 |
| C | 5.665392 | 12.91678 | 8.204064 |
| C | 6.016719 | 3.679927 | 5.020387 |
| C | 6.777264 | 10.65059 | 8.15566 |
| C | 9.25559 | 3.518574 | 4.638059 |
| C | 8.979722 | 2.065971 | 4.975307 |
| C | 2.845791 | 10.78781 | 4.02366 |
| C | 1.208534 | 6.154399 | 5.202048 |
| C | 1.420514 | 9.72988 | 7.69963 |
| C | 6.368357 | 4.003667 | 6.465623 |
| C | 3.115534 | 6.498348 | 1.342014 |
| C | 6.215072 | 11.79855 | 7.305913 |
| C | 7.600015 | 3.526699 | 1.499338 |
| C | 4.961234 | 13.98262 | 4.300659 |
| C | 1.585049 | 7.063814 | 6.181892 |
| C | 11.24244 | 8.206903 | 6.251377 |
| C | 8.181897 | 2.136999 | 1.194684 |
| C | 11.33943 | 7.434167 | 3.802426 |
| C | 11.22091 | 5.16793 | 4.578048 |
| C | 4.801711 | 12.47819 | 4.190038 |
| C | 2.66905 | 9.176023 | 6.996215 |
| C | 11.71615 | 7.661345 | 2.451417 |
| C | 4.71754 | 3.264922 | 4.712359 |
| C | 2.662441 | 8.464633 | 4.518914 |
| C | 12.58675 | 9.843355 | 3.081794 |
| C | 6.343237 | 8.376122 | 1.184145 |
| C | 3.602544 | 8.508508 | 8.015226 |
| C | 6.967055 | 3.783141 | 3.973354 |
| C | 2.288626 | 7.534816 | 3.51467 |
| C | 12.3418 | 8.86691 | 2.12232 |
| C | 6.593535 | 3.464228 | 2.640425 |
| C | 3.493405 | 12.04034 | 3.899062 |
| C | 7.254384 | 12.32069 | 6.32449 |
| C | 2.305308 | 8.220504 | 5.869192 |
| C | 2.604811 | 7.763784 | 2.043427 |
| C | 11.5046 | 6.603001 | 1.376292 |
| C | 9.429408 | 13.3579 | 5.98979 |
| C | 9.234578 | 13.31036 | 4.615621 |
| C | 1.561546 | 6.397842 | 3.880963 |
| C | 10.58792 | 3.934548 | 4.829748 |
| C | 8.439774 | 12.86242 | 6.829868 |
| C | 7.055025 | 12.28342 | 4.92055 |
| C | 8.063735 | 12.78558 | 4.059436 |
| C | 12.70579 | 5.204201 | 4.883766 |
| C | 7.551496 | 7.927324 | 1.803714 |
| H | 5.651439 | 7.583471 | 0.889731 |
| H | 4.930658 | 9.856418 | 0.545131 |
| H | 6.543368 | 10.51469 | 1.158221 |
| H | 6.935338 | 8.083055 | 5.059021 |
| H | 6.526282 | 1.904083 | 7.075017 |
| H | 6.416334 | 3.053367 | 8.42113 |
| H | 4.97809 | 2.650796 | 7.48131 |
| H | 12.81077 | 9.423229 | 7.177225 |
| H | 12.19671 | 8.125779 | 8.203924 |
| H | 13.29645 | 7.753964 | 6.863102 |
| H | 4.573877 | 5.175003 | 6.89341 |
| H | 5.930279 | 5.500116 | 7.985254 |
| H | 5.935362 | 6.151688 | 6.339163 |
| H | 9.184471 | 8.96271 | 6.107606 |
| H | 9.846128 | 8.951206 | 7.746548 |
| H | 10.36353 | 10.1823 | 6.583959 |
| H | 6.923729 | 12.39823 | 2.30924 |
| H | 3.334683 | 2.614845 | 3.196995 |
| H | 0.98266 | 11.36147 | 4.943325 |
| H | 1.00635 | 11.54402 | 3.194564 |
| H | 0.837898 | 9.927855 | 3.919233 |
| H | 13.19081 | 5.34527 | 1.996649 |
| H | 12.63937 | 5.056937 | 0.340451 |
| H | 13.58335 | 6.50692 | 0.721937 |
| H | 4.995731 | 2.777782 | 1.376448 |
| H | 6.561521 | 5.120756 | 0.411977 |
| H | 7.82025 | 4.292928 | -0.51957 |
| H | 6.26931 | 3.506072 | -0.24306 |
| H | 9.078865 | 14.56367 | 2.059822 |
| H | 7.890911 | 14.10958 | 0.834534 |
| H | 7.365034 | 14.87807 | 2.340013 |
| H | 11.65899 | 7.833008 | -0.43208 |
| H | 10.7202 | 6.357056 | -0.6306 |
| H | 10.02021 | 7.7373 | 0.229395 |
| H | 1.049422 | 9.2722 | 1.705673 |
| H | 1.63567 | 8.489985 | 0.230966 |
| H | 0.550689 | 7.618258 | 1.327253 |
| H | 12.40784 | 10.37791 | 5.149796 |
| H | 8.885007 | 10.8038 | 2.347994 |
| H | 8.804239 | 11.7244 | 0.834277 |
| H | 9.975343 | 12.15734 | 2.089015 |
| H | 5.229733 | 13.73102 | 7.616816 |
| H | 4.885176 | 12.52744 | 8.867654 |
| H | 6.451143 | 13.34723 | 8.835135 |
| H | 7.641544 | 10.97517 | 8.745873 |
| H | 6.020383 | 10.28244 | 8.855824 |
| H | 7.093896 | 9.812721 | 7.5275 |
| H | 9.872027 | 1.452809 | 4.831696 |
| H | 8.68546 | 1.977732 | 6.027814 |
| H | 8.163503 | 1.654976 | 4.377854 |
| H | 0.641107 | 5.26554 | 5.464544 |
| H | 0.869427 | 8.937993 | 8.219294 |
| H | 1.702518 | 10.47932 | 8.4475 |
| H | 0.731211 | 10.19951 | 6.991919 |
| H | 7.44767 | 4.184519 | 6.511204 |
| H | 2.326499 | 5.745601 | 1.238558 |
| H | 3.456444 | 6.738671 | 0.328822 |
| H | 3.942 | 6.030852 | 1.88582 |
| H | 5.380128 | 11.40214 | 6.720076 |
| H | 8.432512 | 4.155758 | 1.837533 |
| H | 5.735168 | 14.34798 | 3.620429 |
| H | 4.028032 | 14.50243 | 4.079595 |
| H | 5.282227 | 14.26137 | 5.309246 |
| H | 1.30799 | 6.878014 | 7.216867 |
| H | 10.89938 | 7.172268 | 6.360038 |
| H | 7.390726 | 1.445364 | 0.883254 |
| H | 8.917509 | 2.194718 | 0.384529 |
| H | 8.678454 | 1.706378 | 2.068168 |
| H | 3.207519 | 10.02124 | 6.557346 |
| H | 3.981197 | 3.194027 | 5.508585 |
| H | 13.06956 | 10.77591 | 2.802575 |
| H | 4.532429 | 8.175165 | 7.545937 |
| H | 3.857234 | 9.208257 | 8.8186 |
| H | 3.128726 | 7.635514 | 8.477854 |
| H | 12.64384 | 9.044902 | 1.093734 |
| H | 2.801217 | 12.85278 | 3.703983 |
| H | 3.394087 | 8.523044 | 1.989645 |
| H | 10.7788 | 5.882101 | 1.770593 |
| H | 10.34217 | 13.77949 | 6.402358 |
| H | 10.00707 | 13.69535 | 3.954366 |
| H | 1.261461 | 5.690973 | 3.112482 |
| H | 11.25024 | 3.158686 | 5.199265 |
| H | 8.587009 | 12.89893 | 7.906625 |
| H | 13.15797 | 4.217335 | 4.763409 |
| H | 13.23687 | 5.920595 | 4.254194 |
| H | 12.86072 | 5.512633 | 5.924678 |
| H | 7.874474 | 6.946714 | 1.426101 |
| H | 8.382795 | 8.642234 | 1.781669 |
|  |  |  |  |
| 166 |  |  |  |
| Ts-insertion-second-propene | | |  |
| Yb | 8.159399 | 6.627472 | 4.205331 |
| Yb | 5.792916 | 9.314822 | 3.77714 |
| N | 3.474573 | 9.480969 | 4.374118 |
| N | 8.138997 | 4.229721 | 4.176561 |
| N | 5.812333 | 11.62172 | 4.450873 |
| N | 10.52681 | 6.320184 | 4.165452 |
| C | 7.559059 | 7.226919 | 6.797882 |
| C | 6.770308 | 8.113107 | 5.81792 |
| C | 12.60272 | 5.211824 | 4.8822 |
| C | 8.018166 | 12.69063 | 4.090829 |
| C | 6.970259 | 12.28554 | 4.953658 |
| C | 8.243772 | 13.11141 | 6.846999 |
| C | 10.44264 | 4.014179 | 4.968478 |
| C | 1.619402 | 6.234293 | 4.396554 |
| C | 9.153181 | 13.29791 | 4.637317 |
| C | 9.269469 | 13.52149 | 6.003123 |
| C | 11.42635 | 6.290062 | 1.377285 |
| C | 2.718022 | 7.382085 | 2.446909 |
| C | 2.295367 | 8.289279 | 6.172647 |
| C | 7.09688 | 12.48377 | 6.353039 |
| C | 3.49327 | 11.83278 | 3.710426 |
| C | 6.818979 | 3.250217 | 2.357354 |
| C | 12.48154 | 8.517222 | 1.872345 |
| C | 2.331869 | 7.33715 | 3.916319 |
| C | 6.951811 | 3.567438 | 3.734779 |
| C | 3.454527 | 8.82728 | 8.355092 |
| C | 6.726885 | 8.48989 | 1.183324 |
| C | 12.81836 | 9.576582 | 2.706129 |
| C | 2.682387 | 8.378596 | 4.810791 |
| C | 4.720289 | 2.699261 | 4.123122 |
| C | 11.75452 | 7.420265 | 2.342607 |
| C | 2.631156 | 9.377252 | 7.182575 |
| C | 4.771364 | 12.34492 | 4.038738 |
| C | 11.10341 | 5.221738 | 4.65938 |
| C | 11.35066 | 7.39221 | 3.702242 |
| C | 8.697737 | 2.197274 | 1.044498 |
| C | 11.32246 | 8.488099 | 6.032934 |
| C | 1.572314 | 7.172102 | 6.600686 |
| C | 4.914838 | 13.84814 | 3.880006 |
| C | 7.94705 | 3.498764 | 1.36682 |
| C | 6.003238 | 12.05251 | 7.31901 |
| C | 3.858618 | 6.399253 | 2.147305 |
| C | 5.960054 | 3.593949 | 6.11338 |
| C | 1.368642 | 10.0821 | 7.701876 |
| C | 1.233575 | 6.14499 | 5.728026 |
| C | 2.864546 | 10.59074 | 3.95307 |
| C | 8.972232 | 2.039649 | 4.968458 |
| C | 9.152592 | 3.517291 | 4.669781 |
| C | 6.55998 | 11.34841 | 8.562697 |
| C | 5.885839 | 3.28724 | 4.625527 |
| C | 5.117545 | 13.23879 | 7.731017 |
| C | 8.912065 | 11.33304 | 2.172577 |
| C | 12.42893 | 9.54008 | 4.038772 |
| C | 1.530943 | 7.121353 | 1.509215 |
| C | 5.755865 | 9.500277 | 1.201381 |
| C | 10.51641 | 6.773195 | 0.239523 |
| C | 11.70334 | 8.464125 | 4.560185 |
| C | 8.250631 | 13.73195 | 1.778103 |
| C | 7.462031 | 4.169934 | 0.075697 |
| C | 5.638778 | 2.650209 | 1.909885 |
| C | 12.69346 | 5.63636 | 0.805682 |
| C | 1.366624 | 10.56766 | 3.712107 |
| C | 4.589977 | 2.374566 | 2.779265 |
| C | 7.958265 | 12.46188 | 2.589131 |
| C | 10.31284 | 9.606525 | 6.326671 |
| C | 4.944031 | 4.673732 | 6.512134 |
| C | 12.54464 | 8.628369 | 6.95305 |
| C | 5.759238 | 2.338011 | 6.975199 |
| H | 7.378344 | 6.14891 | 6.64248 |
| H | 8.648539 | 7.378227 | 6.721268 |
| H | 5.697919 | 7.968842 | 6.043185 |
| H | 6.972816 | 9.161345 | 6.104799 |
| H | 12.91712 | 6.06449 | 5.489992 |
| H | 13.13126 | 5.302544 | 3.927644 |
| H | 12.93072 | 4.292376 | 5.369156 |
| H | 8.335819 | 13.28168 | 7.916274 |
| H | 11.09397 | 3.267449 | 5.40894 |
| H | 1.355544 | 5.434508 | 3.709182 |
| H | 9.958068 | 13.60785 | 3.975487 |
| H | 10.1524 | 14.00905 | 6.407868 |
| H | 10.88284 | 5.523372 | 1.938434 |
| H | 3.082582 | 8.39309 | 2.236197 |
| H | 2.820066 | 12.58472 | 3.314544 |
| H | 12.79185 | 8.539457 | 0.830429 |
| H | 2.903886 | 8.06099 | 8.912136 |
| H | 3.700284 | 9.629376 | 9.059571 |
| H | 4.39137 | 8.382022 | 8.007831 |
| H | 13.38474 | 10.42041 | 2.321618 |
| H | 3.901144 | 2.485986 | 4.805214 |
| H | 3.242843 | 10.12378 | 6.66738 |
| H | 9.111315 | 1.73684 | 1.946229 |
| H | 9.526696 | 2.388325 | 0.353685 |
| H | 8.029068 | 1.467679 | 0.57347 |
| H | 10.84957 | 7.526821 | 6.264857 |
| H | 1.266552 | 7.108133 | 7.64234 |
| H | 5.465937 | 14.28592 | 4.715806 |
| H | 3.942679 | 14.33759 | 3.801861 |
| H | 5.484653 | 14.07898 | 2.972619 |
| H | 8.661075 | 4.174932 | 1.849844 |
| H | 5.370833 | 11.33578 | 6.784372 |
| H | 4.749054 | 6.611152 | 2.753465 |
| H | 4.151763 | 6.448042 | 1.092973 |
| H | 3.565717 | 5.368393 | 2.370754 |
| H | 6.966036 | 3.976394 | 6.320225 |
| H | 0.78554 | 10.52287 | 6.887801 |
| H | 1.635833 | 10.88744 | 8.395207 |
| H | 0.714499 | 9.385606 | 8.238759 |
| H | 0.669445 | 5.286018 | 6.081589 |
| H | 8.227232 | 1.579089 | 4.317385 |
| H | 8.627117 | 1.910988 | 6.001172 |
| H | 9.915785 | 1.499325 | 4.868435 |
| H | 7.209715 | 10.51112 | 8.291006 |
| H | 5.740285 | 10.95614 | 9.172999 |
| H | 7.136393 | 12.02932 | 9.198879 |
| H | 5.710012 | 14.01241 | 8.233353 |
| H | 4.333332 | 12.91388 | 8.42422 |
| H | 4.628976 | 13.6983 | 6.867314 |
| H | 9.952661 | 11.59105 | 2.395402 |
| H | 8.835973 | 11.13132 | 1.098423 |
| H | 8.69506 | 10.40163 | 2.708321 |
| H | 12.69645 | 10.36421 | 4.695332 |
| H | 1.163504 | 6.092768 | 1.592285 |
| H | 1.8287 | 7.277003 | 0.46654 |
| H | 0.691929 | 7.790063 | 1.725559 |
| H | 9.576279 | 7.18139 | 0.623499 |
| H | 10.27477 | 5.949821 | -0.44111 |
| H | 10.99933 | 7.559168 | -0.35134 |
| H | 7.603451 | 14.56165 | 2.078704 |
| H | 8.092529 | 13.54668 | 0.710068 |
| H | 9.287991 | 14.062 | 1.900917 |
| H | 6.807225 | 3.51334 | -0.50723 |
| H | 8.312634 | 4.425063 | -0.56441 |
| H | 6.906826 | 5.089815 | 0.283771 |
| H | 5.54106 | 2.390571 | 0.859197 |
| H | 13.26838 | 6.340638 | 0.19422 |
| H | 12.43139 | 4.784163 | 0.168975 |
| H | 13.35441 | 5.272235 | 1.598077 |
| H | 0.831556 | 10.23181 | 4.604795 |
| H | 1.117738 | 9.855481 | 2.918033 |
| H | 0.98799 | 11.5503 | 3.427252 |
| H | 3.680314 | 1.907246 | 2.41198 |
| H | 6.939195 | 12.14344 | 2.346459 |
| H | 10.7406 | 10.59232 | 6.119492 |
| H | 10.00755 | 9.592029 | 7.378646 |
| H | 9.410073 | 9.516625 | 5.713739 |
| H | 5.066418 | 5.589265 | 5.925989 |
| H | 5.048057 | 4.933298 | 7.571361 |
| H | 3.917012 | 4.330067 | 6.353519 |
| H | 13.29209 | 7.851698 | 6.764374 |
| H | 12.23993 | 8.555894 | 8.002768 |
| H | 13.03702 | 9.59788 | 6.821622 |
| H | 4.743171 | 1.941916 | 6.871827 |
| H | 5.912161 | 2.573267 | 8.034153 |
| H | 6.453224 | 1.538796 | 6.699423 |
| H | 6.050668 | 10.53837 | 1.078044 |
| H | 4.719779 | 9.263901 | 0.976501 |
| H | 7.750275 | 8.753992 | 0.924609 |
| H | 7.156537 | 7.923343 | 2.53785 |
| C | 7.236761 | 7.455582 | 8.286316 |
| H | 6.155399 | 7.506022 | 8.457259 |
| H | 7.631941 | 6.645088 | 8.909244 |
| H | 7.672416 | 8.393381 | 8.649424 |
| C | 6.396695 | 7.315143 | 0.243886 |
| H | 7.175818 | 7.179876 | -0.51491 |
| H | 6.308623 | 6.373699 | 0.797993 |
| H | 5.448982 | 7.478709 | -0.28166 |
|  |  |  |  |
| 166 |  |  |  |
| Product-second-insertion | | |  |
| Yb | 5.791239 | 9.179727 | 4.17431 |
| Yb | 8.050114 | 6.667851 | 4.459063 |
| N | 10.42282 | 6.451994 | 4.756803 |
| N | 5.82606 | 11.57097 | 3.948594 |
| N | 8.094826 | 4.294927 | 4.836019 |
| N | 3.445071 | 9.468974 | 3.756572 |
| C | 8.545023 | 2.785486 | 8.177915 |
| C | 12.31231 | 6.02519 | 8.084934 |
| C | 7.120143 | 4.736878 | 8.892417 |
| C | 10.3392 | 7.442067 | 8.754982 |
| C | 6.312466 | 12.18034 | 1.14474 |
| C | 4.495746 | 2.547088 | 6.234675 |
| C | 1.302499 | 10.61977 | 4.20153 |
| C | 12.6236 | 8.440888 | 1.858866 |
| C | 4.722613 | 2.668757 | 4.870442 |
| C | 5.092684 | 4.292638 | 2.238925 |
| C | 5.570261 | 13.44394 | 0.682163 |
| C | 11.47194 | 7.811703 | 6.510088 |
| C | 10.33299 | 9.43343 | 2.175034 |
| C | 6.879767 | 7.77555 | 2.384718 |
| C | 1.986992 | 10.1488 | 0.375169 |
| C | 12.16556 | 8.964863 | 6.887057 |
| C | 6.949569 | 11.46803 | -0.05519 |
| C | 7.958658 | 13.53976 | 6.940204 |
| C | 5.839111 | 8.84265 | 7.023819 |
| C | 6.665104 | 3.550967 | 6.681227 |
| C | 8.70894 | 11.16543 | 6.570052 |
| C | 9.15915 | 3.561118 | 4.513956 |
| C | 9.039341 | 2.051089 | 4.410119 |
| C | 2.818418 | 10.58896 | 4.114563 |
| C | 1.379053 | 6.111447 | 2.200387 |
| C | 0.838551 | 7.48467 | 6.295369 |
| C | 7.713971 | 3.984915 | 7.695368 |
| C | 6.75481 | 7.883458 | 6.245984 |
| C | 3.874944 | 8.623279 | -0.30668 |
| C | 7.783623 | 12.28485 | 6.07216 |
| C | 6.088725 | 3.304709 | 2.862583 |
| C | 4.919757 | 13.78836 | 4.539506 |
| C | 1.501075 | 6.275365 | 3.573821 |
| C | 11.08484 | 6.798547 | 7.578398 |
| C | 5.965808 | 1.933285 | 2.181053 |
| C | 11.18367 | 7.599097 | 5.136031 |
| C | 11.05643 | 5.325234 | 4.422554 |
| C | 4.760269 | 12.29943 | 4.289691 |
| C | 2.226474 | 7.509979 | 5.637892 |
| C | 11.59481 | 8.558503 | 4.1776 |
| C | 5.467005 | 2.987117 | 7.126791 |
| C | 2.701089 | 8.359158 | 3.253811 |
| C | 12.56456 | 9.911884 | 5.951017 |
| C | 7.764076 | 6.790379 | 1.602828 |
| C | 3.11786 | 6.416537 | 6.242727 |
| C | 6.896549 | 3.661668 | 5.284197 |
| C | 2.584272 | 8.194133 | 1.84838 |
| C | 12.27244 | 9.703343 | 4.609546 |
| C | 5.909648 | 3.215167 | 4.370824 |
| C | 3.444262 | 11.81228 | 4.448178 |
| C | 8.007381 | 12.56115 | 4.59318 |
| C | 2.148024 | 7.387003 | 4.123778 |
| C | 3.118018 | 9.237273 | 0.87734 |
| C | 11.33195 | 8.387377 | 2.68904 |
| C | 9.475428 | 13.43812 | 2.863335 |
| C | 8.53035 | 13.10919 | 1.898463 |
| C | 1.91759 | 7.07142 | 1.351239 |
| C | 10.45101 | 4.064037 | 4.234497 |
| C | 9.209793 | 13.15523 | 4.196729 |
| C | 7.045127 | 12.23116 | 3.606739 |
| C | 7.319673 | 12.50036 | 2.240061 |
| C | 12.55963 | 5.345788 | 4.214276 |
| H | 7.71263 | 5.759379 | 1.995972 |
| H | 8.822999 | 7.081205 | 1.633243 |
| H | 5.825793 | 7.517258 | 2.154251 |
| H | 7.019342 | 8.768097 | 1.911674 |
| H | 4.80015 | 8.834367 | 6.652039 |
| H | 6.185004 | 9.88409 | 6.958711 |
| H | 7.758036 | 7.953369 | 6.711527 |
| H | 6.420376 | 6.853872 | 6.486797 |
| H | 9.054386 | 2.286935 | 7.348714 |
| H | 9.307985 | 3.106894 | 8.895968 |
| H | 7.906777 | 2.044958 | 8.67363 |
| H | 13.03747 | 6.700839 | 8.552993 |
| H | 12.0177 | 5.280191 | 8.832644 |
| H | 12.82259 | 5.500287 | 7.272393 |
| H | 6.497159 | 4.088776 | 9.518752 |
| H | 7.923293 | 5.12082 | 9.529962 |
| H | 6.509155 | 5.585454 | 8.57102 |
| H | 9.449643 | 7.983788 | 8.420112 |
| H | 10.01972 | 6.673692 | 9.467169 |
| H | 10.97302 | 8.147467 | 9.303652 |
| H | 5.567597 | 11.50502 | 1.579104 |
| H | 3.570547 | 2.110842 | 6.601564 |
| H | 0.982169 | 10.3195 | 5.206199 |
| H | 0.917044 | 11.62493 | 4.019404 |
| H | 0.840411 | 9.9278 | 3.494948 |
| H | 13.37236 | 7.73136 | 2.223432 |
| H | 12.41361 | 8.205819 | 0.809634 |
| H | 13.07558 | 9.438224 | 1.885778 |
| H | 3.963674 | 2.326715 | 4.17122 |
| H | 5.171243 | 5.288834 | 2.684133 |
| H | 5.264496 | 4.390705 | 1.161409 |
| H | 4.060188 | 3.95824 | 2.382832 |
| H | 6.269716 | 14.17874 | 0.267076 |
| H | 4.838692 | 13.19966 | -0.09626 |
| H | 5.033939 | 13.92132 | 1.506981 |
| H | 10.71809 | 10.44932 | 2.307351 |
| H | 10.13142 | 9.288388 | 1.107752 |
| H | 9.377866 | 9.380233 | 2.707538 |
| H | 1.484509 | 10.66009 | 1.201116 |
| H | 2.378092 | 10.91352 | -0.30551 |
| H | 1.232083 | 9.569024 | -0.16835 |
| H | 12.40087 | 9.121592 | 7.936729 |
| H | 7.490042 | 10.56743 | 0.251071 |
| H | 6.177148 | 11.17224 | -0.7729 |
| H | 7.653646 | 12.11499 | -0.58983 |
| H | 7.341151 | 14.37053 | 6.585564 |
| H | 7.680249 | 13.32672 | 7.978111 |
| H | 8.998895 | 13.88264 | 6.946414 |
| H | 9.762143 | 11.44226 | 6.459829 |
| H | 8.527401 | 10.95061 | 7.629072 |
| H | 8.561369 | 10.23696 | 6.008824 |
| H | 8.725719 | 1.772144 | 3.397569 |
| H | 9.996851 | 1.563975 | 4.604961 |
| H | 8.291647 | 1.654514 | 5.099637 |
| H | 0.864679 | 5.24497 | 1.793571 |
| H | 0.356204 | 6.508306 | 6.175908 |
| H | 0.921488 | 7.678337 | 7.370525 |
| H | 0.171127 | 8.236012 | 5.863384 |
| H | 8.397512 | 4.667472 | 7.178054 |
| H | 3.215318 | 8.041835 | -0.96025 |
| H | 4.317452 | 9.414239 | -0.92124 |
| H | 4.681962 | 7.966061 | 0.030365 |
| H | 6.748039 | 11.94628 | 6.189226 |
| H | 7.100749 | 3.678393 | 2.671342 |
| H | 5.585096 | 14.24816 | 3.804934 |
| H | 3.95777 | 14.30301 | 4.516134 |
| H | 5.370266 | 13.95965 | 5.523766 |
| H | 1.079122 | 5.526688 | 4.23974 |
| H | 10.41063 | 6.074136 | 7.110039 |
| H | 4.948567 | 1.53517 | 2.264673 |
| H | 6.197886 | 2.014743 | 1.113414 |
| H | 6.643548 | 1.196903 | 2.622531 |
| H | 2.679331 | 8.481628 | 5.864666 |
| H | 5.291796 | 2.888374 | 8.194486 |
| H | 13.10327 | 10.80201 | 6.264792 |
| H | 4.124786 | 6.42787 | 5.814695 |
| H | 3.211077 | 6.544578 | 7.326756 |
| H | 2.703192 | 5.420136 | 6.058977 |
| H | 12.58362 | 10.442 | 3.875085 |
| H | 2.742745 | 12.57298 | 4.77343 |
| H | 3.821856 | 9.866201 | 1.433178 |
| H | 10.88984 | 7.394676 | 2.54614 |
| H | 10.41055 | 13.91228 | 2.577462 |
| H | 8.735916 | 13.33266 | 0.854885 |
| H | 1.814789 | 6.948204 | 0.276511 |
| H | 11.15049 | 3.293847 | 3.927779 |
| H | 9.950277 | 13.40587 | 4.952158 |
| H | 12.79686 | 5.78758 | 3.239895 |
| H | 13.06034 | 5.958978 | 4.967055 |
| H | 12.98182 | 4.339851 | 4.238787 |
| C | 7.414297 | 6.645797 | 0.110062 |
| H | 8.317634 | 6.599468 | -0.50876 |
| H | 6.838107 | 5.732729 | -0.07814 |
| H | 6.815263 | 7.492811 | -0.24326 |
| C | 5.712971 | 8.54469 | 8.529445 |
| H | 4.663303 | 8.458748 | 8.832627 |
| H | 6.210746 | 7.604615 | 8.793238 |
| H | 6.166815 | 9.339099 | 9.132809 |
|  |  |  |  |
| 166 |  |  |  |
| Ts-allyl-after-first | |  |  |
| Yb | 5.772587 | 9.310784 | 3.871266 |
| Yb | 8.162134 | 6.700744 | 4.24174 |
| N | 10.54995 | 6.518558 | 4.325062 |
| N | 5.843988 | 11.65077 | 4.400953 |
| N | 8.277246 | 4.293857 | 4.225059 |
| N | 3.45689 | 9.57338 | 4.442061 |
| C | 7.178014 | 7.284384 | 8.363726 |
| C | 7.496725 | 7.166239 | 6.870125 |
| C | 6.697065 | 8.074608 | 5.922651 |
| C | 5.928736 | 2.206988 | 6.941573 |
| C | 12.47372 | 8.743013 | 7.263129 |
| C | 5.007756 | 4.497632 | 6.472109 |
| C | 10.20601 | 9.657149 | 6.676742 |
| C | 7.988403 | 12.35752 | 2.478512 |
| C | 4.897532 | 2.220118 | 2.711226 |
| C | 1.357288 | 10.70426 | 3.820894 |
| C | 12.68849 | 6.078954 | 0.979718 |
| C | 5.944678 | 2.583334 | 1.873292 |
| C | 7.695544 | 4.411696 | 0.194075 |
| C | 8.319437 | 13.58853 | 1.622477 |
| C | 11.6495 | 8.67702 | 4.858231 |
| C | 10.54355 | 7.273954 | 0.394908 |
| C | 5.03431 | 10.22129 | 0.861132 |
| C | 1.655106 | 6.983793 | 1.779902 |
| C | 12.34599 | 9.802952 | 4.408 |
| C | 8.884575 | 11.17611 | 2.080556 |
| C | 5.146658 | 13.418 | 7.510726 |
| C | 6.083698 | 3.189103 | 4.603118 |
| C | 6.62295 | 11.67422 | 8.563649 |
| C | 9.327245 | 3.624211 | 4.705496 |
| C | 9.220931 | 2.126846 | 4.92863 |
| C | 2.863581 | 10.68177 | 3.997147 |
| C | 1.219896 | 6.357237 | 6.06286 |
| C | 1.147007 | 10.37575 | 7.766297 |
| C | 6.0942 | 3.479682 | 6.096506 |
| C | 3.919327 | 6.196908 | 2.56597 |
| C | 6.07136 | 12.22035 | 7.241131 |
| C | 8.195485 | 3.595206 | 1.392772 |
| C | 5.000618 | 13.87358 | 3.736933 |
| C | 1.479982 | 7.490246 | 6.824448 |
| C | 11.26338 | 8.600103 | 6.327793 |
| C | 8.928463 | 2.331862 | 0.915879 |
| C | 11.32424 | 7.654047 | 3.932262 |
| C | 11.17884 | 5.435178 | 4.78451 |
| C | 4.815995 | 12.38539 | 3.973875 |
| C | 2.446153 | 9.776006 | 7.207372 |
| C | 11.7143 | 7.786945 | 2.574687 |
| C | 4.975214 | 2.528197 | 4.06328 |
| C | 2.653232 | 8.518274 | 4.966339 |
| C | 12.73441 | 9.935628 | 3.080942 |
| C | 5.288301 | 8.970215 | 0.432089 |
| C | 3.393745 | 9.403951 | 8.356529 |
| C | 7.148513 | 3.5601 | 3.743699 |
| C | 2.378753 | 7.371018 | 4.182253 |
| C | 12.41659 | 8.928458 | 2.177987 |
| C | 7.071053 | 3.252678 | 2.359825 |
| C | 3.519102 | 11.8956 | 3.687266 |
| C | 7.169247 | 12.57229 | 6.247591 |
| C | 2.187076 | 8.57793 | 6.305006 |
| C | 2.826275 | 7.261256 | 2.733627 |
| C | 11.40717 | 6.71821 | 1.535608 |
| C | 9.367411 | 13.52739 | 5.827498 |
| C | 9.233525 | 13.23807 | 4.476351 |
| C | 1.669612 | 6.307939 | 4.75007 |
| C | 10.58365 | 4.179549 | 5.035007 |
| C | 8.337505 | 13.19235 | 6.699317 |
| C | 7.026545 | 12.3071 | 4.860822 |
| C | 8.075869 | 12.63879 | 3.969869 |
| C | 12.67084 | 5.50065 | 5.048122 |
| C | 6.613464 | 8.408188 | 0.119673 |
| H | 7.357972 | 8.306023 | 8.713773 |
| H | 7.787408 | 6.604276 | 8.971441 |
| H | 6.123656 | 7.054437 | 8.548712 |
| H | 7.354422 | 6.102097 | 6.602378 |
| H | 8.580934 | 7.349875 | 6.757335 |
| H | 5.624737 | 7.885731 | 6.128047 |
| H | 6.861848 | 9.114999 | 6.268014 |
| H | 7.212212 | 7.892907 | 2.59311 |
| H | 4.448255 | 8.27502 | 0.387555 |
| H | 4.03374 | 10.55243 | 1.121944 |
| H | 5.815257 | 10.97876 | 0.86979 |
| H | 6.664533 | 1.44107 | 6.679315 |
| H | 6.043359 | 2.438649 | 8.006124 |
| H | 4.935072 | 1.765369 | 6.808604 |
| H | 12.91482 | 9.743543 | 7.198103 |
| H | 12.1707 | 8.5866 | 8.304188 |
| H | 13.26232 | 8.021522 | 7.028547 |
| H | 4.007855 | 4.100688 | 6.26807 |
| H | 5.055657 | 4.742677 | 7.538683 |
| H | 5.103739 | 5.428938 | 5.906236 |
| H | 9.314234 | 9.56692 | 6.04874 |
| H | 9.894337 | 9.564398 | 7.723167 |
| H | 10.59512 | 10.67003 | 6.53308 |
| H | 6.951506 | 12.08009 | 2.262287 |
| H | 4.030977 | 1.697942 | 2.314624 |
| H | 0.859326 | 10.81454 | 4.78976 |
| H | 1.0453 | 11.53782 | 3.188861 |
| H | 0.994023 | 9.769351 | 3.385917 |
| H | 13.30044 | 5.642301 | 1.774865 |
| H | 12.44288 | 5.281806 | 0.269238 |
| H | 13.30587 | 6.814487 | 0.451927 |
| H | 5.891169 | 2.336949 | 0.815752 |
| H | 7.202058 | 5.333628 | 0.515039 |
| H | 8.532101 | 4.685801 | -0.45731 |
| H | 6.982465 | 3.844259 | -0.41414 |
| H | 9.374692 | 13.86925 | 1.707817 |
| H | 8.124766 | 13.38031 | 0.564703 |
| H | 7.722227 | 14.45795 | 1.914365 |
| H | 11.06363 | 8.069732 | -0.14999 |
| H | 10.30407 | 6.485364 | -0.32647 |
| H | 9.602312 | 7.688302 | 0.768679 |
| H | 0.865342 | 7.734085 | 1.884646 |
| H | 1.996394 | 6.994706 | 0.738693 |
| H | 1.206944 | 6.001896 | 1.965813 |
| H | 12.59197 | 10.58989 | 5.116474 |
| H | 8.601794 | 10.25155 | 2.597194 |
| H | 8.821492 | 10.98337 | 1.004036 |
| H | 9.932716 | 11.37324 | 2.327056 |
| H | 4.646662 | 13.75837 | 6.600184 |
| H | 4.370297 | 13.15003 | 8.236396 |
| H | 5.712797 | 14.26221 | 7.921251 |
| H | 7.13163 | 12.44836 | 9.148922 |
| H | 5.806677 | 11.29001 | 9.182986 |
| H | 7.333557 | 10.85963 | 8.39539 |
| H | 10.20271 | 1.674029 | 5.074746 |
| H | 8.616161 | 1.917764 | 5.817533 |
| H | 8.725658 | 1.632875 | 4.089157 |
| H | 0.667673 | 5.523434 | 6.487762 |
| H | 0.640554 | 9.679957 | 8.444416 |
| H | 1.36155 | 11.28784 | 8.333958 |
| H | 0.43871 | 10.63256 | 6.97252 |
| H | 7.069727 | 3.914686 | 6.340908 |
| H | 3.565388 | 5.207289 | 2.870543 |
| H | 4.251113 | 6.126505 | 1.52506 |
| H | 4.80143 | 6.419746 | 3.17937 |
| H | 5.463837 | 11.43365 | 6.779696 |
| H | 8.920769 | 4.208379 | 1.937503 |
| H | 5.544176 | 14.0395 | 2.799519 |
| H | 4.041822 | 14.38992 | 3.666466 |
| H | 5.594224 | 14.33456 | 4.529605 |
| H | 1.126477 | 7.532903 | 7.851844 |
| H | 10.82743 | 7.609965 | 6.502269 |
| H | 8.253334 | 1.66683 | 0.365383 |
| H | 9.755839 | 2.595238 | 0.247475 |
| H | 9.342951 | 1.764931 | 1.754686 |
| H | 2.940669 | 10.54693 | 6.609028 |
| H | 4.159152 | 2.242518 | 4.722508 |
| H | 13.28343 | 10.81459 | 2.753885 |
| H | 4.356426 | 9.04829 | 7.978545 |
| H | 3.577754 | 10.27124 | 9.000314 |
| H | 2.965475 | 8.612697 | 8.982379 |
| H | 12.72252 | 9.026892 | 1.139222 |
| H | 2.857139 | 12.64927 | 3.275166 |
| H | 3.246798 | 8.232436 | 2.447316 |
| H | 10.83651 | 5.928621 | 2.034931 |
| H | 10.26766 | 14.00852 | 6.200486 |
| H | 10.04127 | 13.48936 | 3.793903 |
| H | 1.462309 | 5.427569 | 4.146689 |
| H | 11.27305 | 3.455661 | 5.453803 |
| H | 8.445509 | 13.41421 | 7.757143 |
| H | 13.0702 | 4.529247 | 5.3425 |
| H | 13.20791 | 5.844738 | 4.159391 |
| H | 12.8932 | 6.221117 | 5.841162 |
| H | 6.985675 | 8.055218 | 1.284856 |
| H | 6.584198 | 7.570294 | -0.57947 |
| H | 7.340756 | 9.158028 | -0.20307 |
|  |  |  |  |
| 83 |  |  |  |
| Product | Yb-prop |  |  |
| Yb | -2.42857 | 0.008271 | -2.24545 |
| N | -2.77376 | -1.46219 | -0.44738 |
| N | -2.29452 | 1.473878 | -0.41576 |
| C | -2.40026 | 2.454981 | 1.834363 |
| C | -2.75943 | 0.02278 | 1.465042 |
| C | 0.482831 | 2.46178 | -0.51456 |
| C | -1.80341 | -3.63504 | -1.09809 |
| C | -0.48133 | 4.466555 | -1.70075 |
| C | -2.91966 | -2.76536 | -0.99819 |
| C | -1.52637 | 5.207338 | -2.24114 |
| C | -4.26318 | -4.3669 | -2.23488 |
| C | -0.70358 | 3.247935 | -1.05379 |
| C | -2.48172 | 1.277695 | 0.887437 |
| C | -2.03374 | 2.767808 | -0.9452 |
| C | 0.115107 | -4.33232 | 0.413282 |
| C | -4.54282 | 3.021374 | -1.42917 |
| C | -0.43867 | -3.26165 | -0.53773 |
| C | -5.37236 | -2.21594 | -1.52281 |
| C | -3.18898 | -2.40479 | 1.783611 |
| C | -2.89124 | -1.24335 | 0.860528 |
| C | -4.15928 | -3.13463 | -1.58229 |
| C | -2.82631 | 4.72702 | -2.14213 |
| C | 1.353208 | 1.919415 | -1.65906 |
| C | -3.10414 | 3.514947 | -1.50313 |
| C | 0.560761 | -2.97583 | -1.67025 |
| C | -1.96182 | -4.85554 | -1.75979 |
| C | 1.330164 | 3.288021 | 0.464048 |
| C | -3.17693 | -5.22835 | -2.32339 |
| C | -5.0849 | 2.664082 | -2.8221 |
| C | -5.75161 | -1.69871 | -2.91963 |
| C | -5.4734 | 4.028879 | -0.73996 |
| C | -6.58284 | -2.88653 | -0.85863 |
| H | -3.0405 | 3.274753 | 1.4938 |
| H | -1.37973 | 2.850697 | 1.867196 |
| H | -2.69328 | 2.176561 | 2.847955 |
| H | -2.88789 | 0.032502 | 2.541359 |
| H | 0.08633 | 1.601133 | 0.03419 |
| H | 0.536001 | 4.838778 | -1.79034 |
| H | -1.32742 | 6.150705 | -2.74201 |
| H | -5.21234 | -4.65634 | -2.67986 |
| H | -0.58759 | -4.56705 | 1.219467 |
| H | 1.049554 | -3.98726 | 0.868649 |
| H | 0.335918 | -5.26677 | -0.11417 |
| H | -4.54563 | 2.109862 | -0.82072 |
| H | -0.5585 | -2.33756 | 0.037092 |
| H | -5.10031 | -1.35282 | -0.90437 |
| H | -2.35063 | -3.10901 | 1.798911 |
| H | -4.06049 | -2.96695 | 1.433604 |
| H | -3.37376 | -2.06887 | 2.805184 |
| H | -3.64292 | 5.304762 | -2.56901 |
| H | 0.779889 | 1.279532 | -2.33951 |
| H | 2.186345 | 1.330695 | -1.25864 |
| H | 1.778046 | 2.738937 | -2.25005 |
| H | 0.721439 | -3.86935 | -2.28459 |
| H | 1.530675 | -2.67595 | -1.25737 |
| H | 0.210787 | -2.1757 | -2.33273 |
| H | -1.10961 | -5.52521 | -1.84247 |
| H | 1.820726 | 4.129936 | -0.03701 |
| H | 2.117943 | 2.665593 | 0.901875 |
| H | 0.72853 | 3.697374 | 1.282174 |
| H | -3.27399 | -6.18213 | -2.83453 |
| H | -5.1263 | 3.545135 | -3.47128 |
| H | -6.09481 | 2.244552 | -2.75886 |
| H | -4.45105 | 1.933045 | -3.34447 |
| H | -4.91064 | -1.20408 | -3.42683 |
| H | -6.57787 | -0.98151 | -2.86618 |
| H | -6.05945 | -2.51844 | -3.57744 |
| H | -5.11298 | 4.280381 | 0.261815 |
| H | -6.48369 | 3.616464 | -0.64228 |
| H | -5.55115 | 4.961675 | -1.30885 |
| H | -6.93745 | -3.7439 | -1.44066 |
| H | -7.4155 | -2.18006 | -0.76986 |
| H | -6.33628 | -3.24587 | 0.144754 |
| C | -0.61132 | -0.32098 | -4.26951 |
| H | -0.8114 | -1.23468 | -4.84068 |
| H | -0.6394 | 0.52001 | -4.97168 |
| C | 0.912092 | -0.37917 | -4.05171 |
| H | 1.188638 | -1.20387 | -3.385 |
| H | 1.286708 | 0.547855 | -3.60294 |
| C | 1.661051 | -0.58652 | -5.38124 |
| H | 2.612686 | -1.10751 | -5.2261 |
| H | 1.068703 | -1.18346 | -6.08402 |
| H | 1.88394 | 0.370244 | -5.86708 |
|  |  |  |  |
| 81 |  |  |  |
| Yb-allyl |  |  |  |
| Yb | 0.963921 | 12.44408 | 2.194603 |
| N | 1.095998 | 14.81289 | 2.163027 |
| N | -1.27676 | 13.02942 | 1.719906 |
| C | 2.939321 | 15.14302 | -0.10073 |
| C | -3.34106 | 14.28484 | 1.274646 |
| C | 3.350663 | 16.39516 | -0.89022 |
| C | 2.611516 | 10.58489 | 1.320349 |
| C | -2.5704 | 11.92885 | -2.02942 |
| C | 3.469187 | 13.88002 | -0.79524 |
| C | 2.036571 | 16.6122 | 5.75794 |
| C | 2.111058 | 14.09534 | 5.847034 |
| C | -1.85959 | 14.21917 | 1.577354 |
| C | -3.26882 | 9.313822 | 1.397555 |
| C | -3.99024 | 12.09412 | 4.686318 |
| C | -0.31052 | 10.97707 | -1.4404 |
| C | 1.92651 | 15.31845 | 4.93684 |
| C | 0.548135 | 17.20145 | 2.000366 |
| C | -3.21361 | 9.975041 | 2.618887 |
| C | 0.163255 | 15.73873 | 1.953479 |
| C | -2.58075 | 11.91128 | 4.104309 |
| C | -2.03109 | 11.82747 | 1.593447 |
| C | 2.068796 | 10.08927 | 2.511755 |
| C | -1.68705 | 11.15486 | 5.098655 |
| C | -2.09663 | 11.164 | 0.341198 |
| C | -1.19068 | 15.45457 | 1.686462 |
| C | 2.897302 | 15.31049 | 3.763444 |
| C | -2.60661 | 11.22798 | 2.743709 |
| C | -1.51462 | 11.77911 | -0.92426 |
| C | 5.186157 | 15.49566 | 2.962208 |
| C | 4.737951 | 15.38456 | 1.651122 |
| C | -2.71571 | 9.91256 | 0.271852 |
| C | 4.265438 | 15.46749 | 4.003351 |
| C | 2.449522 | 15.16516 | 2.424278 |
| C | 3.381054 | 15.22496 | 1.354817 |
| C | 2.121685 | 10.68882 | 3.775732 |
| H | 1.376555 | 9.247035 | 2.409764 |
| H | 2.544369 | 9.991896 | 0.413539 |
| H | 3.48725 | 11.23802 | 1.379661 |
| H | 1.845246 | 15.09315 | -0.11044 |
| H | -3.91862 | 13.77464 | 2.051804 |
| H | -3.69151 | 15.31544 | 1.200724 |
| H | -3.56777 | 13.77197 | 0.334383 |
| H | 4.439896 | 16.47939 | -0.97189 |
| H | 2.946429 | 16.35502 | -1.90766 |
| H | 2.984237 | 17.3114 | -0.41693 |
| H | -3.43854 | 12.49983 | -1.68576 |
| H | -2.14604 | 12.44804 | -2.89565 |
| H | -2.93276 | 10.95459 | -2.37528 |
| H | 3.136028 | 12.96428 | -0.29441 |
| H | 3.124049 | 13.83788 | -1.83426 |
| H | 4.564645 | 13.8637 | -0.80792 |
| H | 1.915191 | 17.50176 | 5.132025 |
| H | 1.265634 | 16.63765 | 6.535783 |
| H | 3.008931 | 16.69097 | 6.256334 |
| H | 3.117282 | 14.07102 | 6.280205 |
| H | 1.39285 | 14.1182 | 6.67419 |
| H | 1.967103 | 13.15427 | 5.304959 |
| H | -3.74287 | 8.339016 | 1.323009 |
| H | -4.46243 | 11.13146 | 4.911359 |
| H | -3.94546 | 12.66355 | 5.621127 |
| H | -4.64825 | 12.63069 | 3.995595 |
| H | -0.60386 | 9.95789 | -1.71606 |
| H | 0.118969 | 11.4519 | -2.32976 |
| H | 0.481822 | 10.89468 | -0.68707 |
| H | 0.912588 | 15.27446 | 4.525073 |
| H | 1.346871 | 17.41583 | 1.283461 |
| H | -0.30146 | 17.84944 | 1.779492 |
| H | 0.938826 | 17.46568 | 2.988383 |
| H | -3.65132 | 9.508778 | 3.49816 |
| H | -2.15275 | 12.90948 | 3.963514 |
| H | -0.65956 | 11.05064 | 4.731012 |
| H | -1.64876 | 11.67833 | 6.060563 |
| H | -2.06994 | 10.14494 | 5.283829 |
| H | -1.81826 | 16.32596 | 1.539738 |
| H | -1.16091 | 12.78375 | -0.66852 |
| H | 6.245999 | 15.61207 | 3.170942 |
| H | 5.45719 | 15.42322 | 0.836522 |
| H | -2.76505 | 9.398163 | -0.68474 |
| H | 4.615363 | 15.57105 | 5.027648 |
| H | 1.70481 | 10.16917 | 4.632874 |
| H | 2.962811 | 11.35055 | 4.002858 |
|  |  |  |  |
| 175 |  |  |  |
| Ts-Allyl-after | second | insertion |  |
| Yb | 5.520899 | 9.653533 | 4.901248 |
| Yb | 8.135078 | 6.563755 | 5.016601 |
| N | 10.53203 | 6.398992 | 5.01315 |
| N | 5.769624 | 11.70902 | 3.629062 |
| N | 8.295214 | 4.177422 | 4.845232 |
| N | 3.41108 | 9.480355 | 3.733254 |
| C | 9.669279 | 1.681831 | 6.964927 |
| C | 12.07075 | 5.366191 | 8.034748 |
| C | 8.429842 | 2.719415 | 8.876479 |
| C | 10.43007 | 6.847772 | 9.242752 |
| C | 5.793021 | 12.21601 | 0.712437 |
| C | 5.122808 | 1.777333 | 6.302732 |
| C | 1.242301 | 10.66365 | 3.861441 |
| C | 13.05981 | 8.634255 | 2.576889 |
| C | 5.00169 | 2.414216 | 5.07562 |
| C | 4.932963 | 5.046937 | 3.275402 |
| C | 5.258013 | 13.45449 | -0.02323 |
| C | 11.4074 | 7.550824 | 6.985842 |
| C | 10.77042 | 9.658515 | 2.738391 |
| C | 7.032031 | 8.182461 | 3.320405 |
| C | 2.348263 | 9.915268 | 0.346194 |
| C | 12.04637 | 8.655364 | 7.554364 |
| C | 6.201321 | 11.1378 | -0.30033 |
| C | 7.801117 | 13.86105 | 6.043246 |
| C | 6.341095 | 7.471792 | 8.375093 |
| C | 7.372641 | 2.671877 | 6.548711 |
| C | 9.326681 | 11.8633 | 5.870529 |
| C | 9.270945 | 3.657759 | 4.089257 |
| C | 9.066793 | 2.306134 | 3.428085 |
| C | 2.761587 | 10.63921 | 3.857006 |
| C | 1.241563 | 6.050488 | 2.480972 |
| C | 0.655271 | 7.845913 | 6.393997 |
| C | 8.657922 | 2.77089 | 7.360771 |
| C | 6.677409 | 8.081331 | 7.013463 |
| C | 3.891332 | 8.022124 | -0.2467 |
| C | 8.045909 | 12.51658 | 5.340684 |
| C | 5.867899 | 3.835334 | 3.187275 |
| C | 4.683272 | 13.94461 | 3.673316 |
| C | 1.322832 | 6.365284 | 3.829979 |
| C | 10.94734 | 6.402418 | 7.871128 |
| C | 5.37383 | 2.853467 | 2.1158 |
| C | 11.26684 | 7.486269 | 5.574589 |
| C | 11.14993 | 5.409735 | 4.376086 |
| C | 4.657129 | 12.42733 | 3.776702 |
| C | 2.058157 | 7.765801 | 5.774014 |
| C | 11.77863 | 8.532576 | 4.765242 |
| C | 6.305771 | 1.904051 | 7.023234 |
| C | 2.672618 | 8.322439 | 3.334906 |
| C | 12.5443 | 9.687203 | 6.767254 |
| C | 7.890814 | 7.266699 | 2.426533 |
| C | 2.896845 | 6.713782 | 6.513385 |
| C | 7.238842 | 3.326379 | 5.294141 |
| C | 2.618045 | 7.987365 | 1.956284 |
| C | 12.40924 | 9.61564 | 5.387564 |
| C | 6.046352 | 3.18003 | 4.547043 |
| C | 3.361275 | 11.91086 | 4.027238 |
| C | 8.038462 | 12.67528 | 3.83003 |
| C | 2.024316 | 7.489495 | 4.280134 |
| C | 3.302399 | 8.847089 | 0.904187 |
| C | 11.67966 | 8.531013 | 3.245397 |
| C | 9.195422 | 13.47712 | 1.845606 |
| C | 8.095146 | 13.12826 | 1.074324 |
| C | 1.891941 | 6.861179 | 1.558195 |
| C | 10.5155 | 4.263854 | 3.835775 |
| C | 9.155061 | 13.2496 | 3.214733 |
| C | 6.913303 | 12.32697 | 3.040246 |
| C | 6.949713 | 12.56049 | 1.640236 |
| C | 12.65469 | 5.422387 | 4.172103 |
| C | 7.924041 | 7.594945 | 0.931306 |
| C | 6.624619 | 5.980832 | 8.532322 |
| C | 5.10469 | 11.06579 | 7.233792 |
| C | 3.883096 | 10.45151 | 7.24417 |
| C | 6.322831 | 10.51756 | 7.773885 |
| H | 7.552659 | 6.217141 | 2.515589 |
| H | 8.946631 | 7.261322 | 2.762831 |
| H | 6.007084 | 8.130229 | 2.904671 |
| H | 7.367271 | 9.216686 | 3.109624 |
| H | 5.279075 | 7.658101 | 8.583961 |
| H | 6.889507 | 8.023182 | 9.14994 |
| H | 7.775722 | 8.08095 | 6.858288 |
| H | 6.188759 | 7.470915 | 6.222754 |
| H | 9.984033 | 1.772937 | 5.923454 |
| H | 10.56768 | 1.750245 | 7.588819 |
| H | 9.237429 | 0.684286 | 7.10575 |
| H | 12.94167 | 5.818608 | 8.522406 |
| H | 11.73697 | 4.524138 | 8.651229 |
| H | 12.39463 | 4.969851 | 7.068797 |
| H | 8.143214 | 1.716172 | 9.211511 |
| H | 9.356686 | 2.97363 | 9.401802 |
| H | 7.652464 | 3.416361 | 9.200285 |
| H | 9.674009 | 7.634555 | 9.157805 |
| H | 9.980308 | 5.9992 | 9.768199 |
| H | 11.23613 | 7.225403 | 9.881188 |
| H | 4.979341 | 11.80791 | 1.31843 |
| H | 4.303253 | 1.182049 | 6.696082 |
| H | 0.870546 | 10.37998 | 4.852737 |
| H | 0.858102 | 11.65881 | 3.630969 |
| H | 0.825495 | 9.949194 | 3.148755 |
| H | 13.74668 | 7.861003 | 2.931759 |
| H | 12.96402 | 8.532962 | 1.490273 |
| H | 13.52622 | 9.605725 | 2.773128 |
| H | 4.080373 | 2.310334 | 4.508306 |
| H | 5.303685 | 5.798847 | 3.983018 |
| H | 4.82283 | 5.538804 | 2.30401 |
| H | 3.933595 | 4.756193 | 3.612367 |
| H | 6.002231 | 13.86 | -0.71785 |
| H | 4.368456 | 13.19748 | -0.60898 |
| H | 4.987361 | 14.25535 | 0.670504 |
| H | 11.16939 | 10.6428 | 3.003218 |
| H | 10.6812 | 9.622179 | 1.647405 |
| H | 9.763646 | 9.59316 | 3.160318 |
| H | 2.00516 | 10.59964 | 1.12616 |
| H | 2.845431 | 10.51283 | -0.42628 |
| H | 1.466168 | 9.448094 | -0.10716 |
| H | 12.16197 | 8.707475 | 8.632754 |
| H | 6.512209 | 10.21929 | 0.204082 |
| H | 5.364551 | 10.8983 | -0.96564 |
| H | 7.033756 | 11.47446 | -0.92838 |
| H | 6.859567 | 14.3176 | 5.725183 |
| H | 7.76307 | 13.72915 | 7.130241 |
| H | 8.606887 | 14.56866 | 5.816649 |
| H | 10.20322 | 12.50103 | 5.71486 |
| H | 9.248414 | 11.6848 | 6.947507 |
| H | 9.527674 | 10.90492 | 5.384257 |
| H | 8.417656 | 2.419345 | 2.551758 |
| H | 10.01145 | 1.875483 | 3.091339 |
| H | 8.572504 | 1.597712 | 4.096314 |
| H | 0.679979 | 5.180943 | 2.150004 |
| H | 0.149817 | 6.873887 | 6.377876 |
| H | 0.719051 | 8.162464 | 7.440848 |
| H | 0.014985 | 8.556447 | 5.86298 |
| H | 9.113376 | 3.738196 | 7.116114 |
| H | 3.112438 | 7.598759 | -0.89082 |
| H | 4.520171 | 8.653848 | -0.88111 |
| H | 4.504625 | 7.193912 | 0.121985 |
| H | 7.208302 | 11.86336 | 5.606429 |
| H | 6.853098 | 4.192697 | 2.867528 |
| H | 5.696426 | 14.34237 | 3.614135 |
| H | 4.129637 | 14.28033 | 2.7909 |
| H | 4.181963 | 14.37694 | 4.545353 |
| H | 0.821301 | 5.731235 | 4.557159 |
| H | 10.12507 | 5.896149 | 7.348252 |
| H | 4.350951 | 2.516521 | 2.314195 |
| H | 5.370365 | 3.335509 | 1.132259 |
| H | 6.010574 | 1.965116 | 2.059024 |
| H | 2.543702 | 8.735063 | 5.922121 |
| H | 6.3983 | 1.398782 | 7.979968 |
| H | 13.03767 | 10.53878 | 7.227378 |
| H | 3.91952 | 6.667813 | 6.125246 |
| H | 2.950408 | 6.94009 | 7.583856 |
| H | 2.464722 | 5.712771 | 6.404853 |
| H | 12.80211 | 10.42061 | 4.771817 |
| H | 2.635229 | 12.69473 | 4.220013 |
| H | 4.126197 | 9.368222 | 1.405342 |
| H | 11.23363 | 7.57998 | 2.937106 |
| H | 10.07183 | 13.92621 | 1.38623 |
| H | 8.119671 | 13.30572 | 0.001849 |
| H | 1.833974 | 6.612946 | 0.502456 |
| H | 11.17836 | 3.659902 | 3.224247 |
| H | 10.00591 | 13.53566 | 3.827307 |
| H | 12.88953 | 5.597245 | 3.116366 |
| H | 13.14835 | 6.19461 | 4.762775 |
| H | 13.07717 | 4.4493 | 4.437081 |
| H | 8.553323 | 6.893221 | 0.370422 |
| H | 6.915198 | 7.55942 | 0.50898 |
| H | 8.315276 | 8.604635 | 0.772389 |
| H | 6.368721 | 5.622069 | 9.537076 |
| H | 6.050393 | 5.382332 | 7.815808 |
| H | 7.688135 | 5.764914 | 8.372227 |
| H | 5.182045 | 11.97757 | 6.632534 |
| H | 3.020692 | 10.92266 | 6.784474 |
| H | 3.697346 | 9.5895 | 7.882338 |
| H | 6.192552 | 10.09265 | 8.774165 |
| H | 6.511446 | 9.388313 | 7.210067 |
| H | 7.172386 | 11.20176 | 7.774546 |
|  |  |  |  |
| 169 |  |  |  |
| Ts-first-SN | |  |  |
| Yb | 5.480515 | 9.722318 | 4.939536 |
| Yb | 8.105119 | 6.930807 | 5.566592 |
| N | 10.41939 | 6.517106 | 5.054518 |
| N | 5.775835 | 11.83434 | 3.85219 |
| N | 7.961038 | 4.660052 | 4.843761 |
| N | 3.250909 | 9.770162 | 4.070166 |
| C | 8.133364 | 9.014685 | 10.30544 |
| C | 7.949401 | 2.02343 | 7.456761 |
| C | 12.68127 | 5.517912 | 7.5134 |
| C | 6.701807 | 3.77368 | 8.764352 |
| C | 11.55938 | 7.008865 | 9.181602 |
| C | 6.02625 | 11.72343 | 0.941637 |
| C | 4.083093 | 3.05105 | 5.393389 |
| C | 1.25581 | 11.09857 | 4.72914 |
| C | 12.22494 | 8.727592 | 2.06819 |
| C | 4.428355 | 3.636627 | 4.181487 |
| C | 5.347905 | 6.144116 | 2.439953 |
| C | 5.362644 | 12.74591 | 0.008226 |
| C | 11.73318 | 7.729702 | 6.731249 |
| C | 9.965287 | 9.655462 | 2.676673 |
| C | 1.301986 | 10.70175 | 0.929825 |
| C | 12.42565 | 8.884052 | 7.106686 |
| C | 6.630553 | 10.55962 | 0.143176 |
| C | 7.838316 | 14.44645 | 6.112944 |
| C | 7.856367 | 7.61289 | 9.764976 |
| C | 6.30416 | 3.526515 | 6.258937 |
| C | 9.129723 | 12.28821 | 6.15074 |
| C | 8.900854 | 3.923301 | 4.243381 |
| C | 8.552384 | 2.551793 | 3.695316 |
| C | 2.740852 | 10.94848 | 4.436104 |
| C | 0.726218 | 6.655405 | 2.680411 |
| C | 0.980526 | 7.98587 | 6.847961 |
| C | 7.306849 | 3.418465 | 7.400173 |
| C | 7.882533 | 7.508447 | 8.255934 |
| C | 3.029894 | 9.133492 | -0.01978 |
| C | 7.920873 | 13.0199 | 5.548808 |
| C | 6.02951 | 4.778188 | 2.607676 |
| C | 4.794101 | 14.11527 | 3.966659 |
| C | 1.096683 | 6.70762 | 4.016947 |
| C | 11.58111 | 6.573433 | 7.713095 |
| C | 5.65879 | 3.854919 | 1.437607 |
| C | 11.2319 | 7.634805 | 5.40367 |
| C | 10.93495 | 5.489595 | 4.384115 |
| C | 4.735519 | 12.60804 | 4.146094 |
| C | 2.235916 | 7.725576 | 6.000646 |
| C | 11.46543 | 8.683192 | 4.478271 |
| C | 5.018042 | 3.002698 | 6.420377 |
| C | 2.378472 | 8.730131 | 3.627978 |
| C | 12.63162 | 9.929234 | 6.212086 |
| C | 2.903364 | 6.416443 | 6.436189 |
| C | 6.658346 | 4.101441 | 5.01041 |
| C | 2.009024 | 8.677745 | 2.258764 |
| C | 12.15821 | 9.81926 | 4.91163 |
| C | 5.705189 | 4.161934 | 3.961793 |
| C | 3.470305 | 12.14966 | 4.605507 |
| C | 7.962307 | 12.99264 | 4.028799 |
| C | 1.916323 | 7.727209 | 4.514189 |
| C | 2.452633 | 9.740081 | 1.26536 |
| C | 11.0259 | 8.603032 | 3.022501 |
| C | 9.189874 | 13.5517 | 2.002939 |
| C | 8.19637 | 12.94907 | 1.242504 |
| C | 1.184825 | 7.640431 | 1.814605 |
| C | 10.2325 | 4.320101 | 4.030319 |
| C | 9.067951 | 13.55926 | 3.386441 |
| C | 6.933524 | 12.41335 | 3.243753 |
| C | 7.069018 | 12.37325 | 1.835548 |
| C | 12.37653 | 5.516097 | 3.908681 |
| C | 6.235636 | 8.528944 | 7.577657 |
| C | 5.116903 | 8.271558 | 8.458463 |
| C | 6.523056 | 9.929461 | 7.341638 |
| C | 4.382075 | 9.293455 | 9.014598 |
| C | 5.752301 | 10.93918 | 7.94731 |
| C | 4.66674 | 10.65468 | 8.762724 |
| H | 9.099871 | 9.396071 | 9.955603 |
| H | 8.156589 | 9.006747 | 11.40012 |
| H | 7.361103 | 9.719436 | 9.988407 |
| H | 8.578604 | 6.903912 | 10.19492 |
| H | 6.867774 | 7.286022 | 10.11657 |
| H | 8.773402 | 7.993283 | 7.825661 |
| H | 7.791521 | 6.451812 | 7.951561 |
| H | 8.48082 | 1.78527 | 6.531865 |
| H | 8.668492 | 1.962436 | 8.281434 |
| H | 7.187315 | 1.252111 | 7.616302 |
| H | 13.67191 | 5.963251 | 7.659504 |
| H | 12.56657 | 4.704383 | 8.23865 |
| H | 12.65054 | 5.079302 | 6.514585 |
| H | 5.981894 | 3.019313 | 9.09966 |
| H | 7.48755 | 3.826364 | 9.525546 |
| H | 6.183219 | 4.737109 | 8.742168 |
| H | 10.84576 | 7.81732 | 9.36231 |
| H | 11.28184 | 6.161822 | 9.817499 |
| H | 12.54501 | 7.348601 | 9.518995 |
| H | 5.249195 | 11.31227 | 1.592746 |
| H | 3.088794 | 2.637566 | 5.538385 |
| H | 0.676867 | 10.24529 | 4.375746 |
| H | 1.110884 | 11.18539 | 5.812128 |
| H | 0.857059 | 12.00985 | 4.276056 |
| H | 13.01531 | 8.009435 | 2.305796 |
| H | 11.90649 | 8.556221 | 1.034024 |
| H | 12.66588 | 9.729544 | 2.111559 |
| H | 3.690729 | 3.686074 | 3.385066 |
| H | 5.693617 | 6.854145 | 3.199862 |
| H | 5.58498 | 6.573692 | 1.460061 |
| H | 4.259152 | 6.060949 | 2.52053 |
| H | 6.088501 | 13.18503 | -0.68549 |
| H | 4.578524 | 12.27125 | -0.59197 |
| H | 4.907359 | 13.56577 | 0.57239 |
| H | 10.33645 | 10.6726 | 2.838421 |
| H | 9.680462 | 9.574689 | 1.622007 |
| H | 9.058885 | 9.525976 | 3.276035 |
| H | 0.90499 | 11.18428 | 1.827212 |
| H | 1.63981 | 11.48763 | 0.244801 |
| H | 0.475005 | 10.16839 | 0.446833 |
| H | 12.8138 | 8.968855 | 8.117027 |
| H | 7.062363 | 9.807238 | 0.810224 |
| H | 5.866445 | 10.07271 | -0.47098 |
| H | 7.424535 | 10.9036 | -0.52909 |
| H | 6.958883 | 14.98228 | 5.746028 |
| H | 7.786828 | 14.42368 | 7.207411 |
| H | 8.722901 | 15.03144 | 5.837565 |
| H | 10.0615 | 12.82717 | 5.948226 |
| H | 9.030203 | 12.20402 | 7.238512 |
| H | 9.243987 | 11.28312 | 5.734076 |
| H | 8.084509 | 2.648468 | 2.709091 |
| H | 9.447599 | 1.93807 | 3.578308 |
| H | 7.839752 | 2.023999 | 4.332374 |
| H | 0.082324 | 5.858717 | 2.317591 |
| H | 0.255135 | 7.172205 | 6.735202 |
| H | 1.243915 | 8.054309 | 7.908936 |
| H | 0.481843 | 8.915588 | 6.561191 |
| H | 8.115063 | 4.130543 | 7.190118 |
| H | 2.272851 | 8.583701 | -0.58957 |
| H | 3.411126 | 9.92408 | -0.67489 |
| H | 3.850491 | 8.443272 | 0.197074 |
| H | 7.015114 | 12.4895 | 5.859145 |
| H | 7.110954 | 4.94447 | 2.568074 |
| H | 4.196125 | 14.40094 | 3.094066 |
| H | 4.352888 | 14.61635 | 4.833179 |
| H | 5.807174 | 14.48585 | 3.81575 |
| H | 0.732131 | 5.944734 | 4.700063 |
| H | 10.62447 | 6.081769 | 7.491545 |
| H | 4.57406 | 3.736756 | 1.342487 |
| H | 6.022425 | 4.27592 | 0.494075 |
| H | 6.09056 | 2.855767 | 1.552951 |
| H | 2.934968 | 8.545931 | 6.204211 |
| H | 4.74489 | 2.544071 | 7.366805 |
| H | 13.16763 | 10.82033 | 6.527316 |
| H | 3.837079 | 6.234498 | 5.895846 |
| H | 3.125892 | 6.442413 | 7.506139 |
| H | 2.251859 | 5.555651 | 6.256342 |
| H | 12.33281 | 10.63012 | 4.209031 |
| H | 2.844187 | 12.96217 | 4.962687 |
| H | 3.244586 | 10.32236 | 1.745931 |
| H | 10.57539 | 7.617773 | 2.863708 |
| H | 10.05327 | 14.0047 | 1.523219 |
| H | 8.291977 | 12.92798 | 0.159656 |
| H | 0.891712 | 7.607941 | 0.768511 |
| H | 10.8341 | 3.589945 | 3.499295 |
| H | 9.84824 | 14.01881 | 3.988298 |
| H | 12.40642 | 5.785166 | 2.846769 |
| H | 12.98319 | 6.241597 | 4.45239 |
| H | 12.8329 | 4.527279 | 4.002478 |
| H | 7.206941 | 8.491789 | 4.324085 |
| H | 4.864783 | 7.24134 | 8.690425 |
| H | 7.460946 | 10.20657 | 6.861564 |
| H | 3.557558 | 9.034512 | 9.67675 |
| H | 6.044131 | 11.9747 | 7.783451 |
| H | 4.07694 | 11.44465 | 9.213838 |
| H | 6.063641 | 7.756513 | 6.371591 |
|  |  |  |  |
| 178 |  |  |  |
| Ts-second-SN-2 | |  |  |
| Yb | 5.612822 | 10.20936 | 4.910044 |
| Yb | 8.224072 | 6.364482 | 5.400161 |
| N | 10.59319 | 6.002423 | 5.705285 |
| N | 5.689429 | 12.30144 | 3.684337 |
| N | 8.194224 | 3.920874 | 5.425145 |
| N | 3.34767 | 10.0646 | 4.043008 |
| C | 7.067246 | 9.459952 | 1.464646 |
| C | 7.818058 | 8.344113 | 2.196736 |
| C | 7.373966 | 8.043085 | 3.631332 |
| C | 7.11296 | 8.375033 | 10.45881 |
| C | 6.886552 | 1.729421 | 8.244875 |
| C | 12.79605 | 7.167508 | 8.786363 |
| C | 5.298485 | 3.65278 | 8.495162 |
| C | 10.70256 | 8.519972 | 8.993588 |
| C | 4.885135 | 13.11271 | 0.97488 |
| C | 4.428866 | 2.174796 | 4.531759 |
| C | 1.599525 | 11.03374 | 5.519488 |
| C | 10.38968 | 5.925489 | 2.745542 |
| C | 5.293689 | 2.543747 | 3.510114 |
| C | 6.868086 | 4.521776 | 1.711864 |
| C | 3.937646 | 14.32064 | 0.91594 |
| C | 11.66639 | 8.002042 | 6.690382 |
| C | 12.88999 | 6.155781 | 2.656277 |
| C | 0.155529 | 11.80694 | 2.094159 |
| C | 12.27439 | 9.246706 | 6.501663 |
| C | 5.075413 | 12.50282 | -0.42137 |
| C | 8.706352 | 14.20795 | 5.668443 |
| C | 6.519382 | 7.153222 | 9.762045 |
| C | 6.056193 | 2.931581 | 6.176098 |
| C | 9.590734 | 12.08409 | 4.620118 |
| C | 9.26845 | 3.14142 | 5.566155 |
| C | 9.162782 | 1.633417 | 5.395526 |
| C | 2.953058 | 11.06522 | 4.830747 |
| C | 0.375407 | 7.453362 | 2.477572 |
| C | 2.450342 | 6.855189 | 6.260757 |
| C | 6.435763 | 3.070791 | 7.643903 |
| C | 7.122132 | 6.847315 | 8.411666 |
| C | 1.862045 | 10.95284 | 0.458709 |
| C | 8.378081 | 13.01527 | 4.759529 |
| C | 7.463316 | 3.428107 | 2.608678 |
| C | 4.837391 | 14.38568 | 4.750109 |
| C | 1.292019 | 6.99684 | 3.412333 |
| C | 11.45387 | 7.510153 | 8.116739 |
| C | 7.796654 | 2.176494 | 1.782365 |
| C | 11.3379 | 7.209726 | 5.555351 |
| C | 11.21005 | 4.835002 | 5.857459 |
| C | 4.791619 | 12.89616 | 4.456975 |
| C | 3.211035 | 7.230317 | 4.981032 |
| C | 11.67332 | 7.673337 | 4.254528 |
| C | 4.81825 | 2.367995 | 5.851543 |
| C | 2.351027 | 9.183616 | 3.527558 |
| C | 12.56542 | 9.725904 | 5.231237 |
| C | 3.967672 | 6.02029 | 4.421832 |
| C | 6.937914 | 3.305007 | 5.126828 |
| C | 1.430517 | 9.646601 | 2.547656 |
| C | 12.26911 | 8.935485 | 4.12952 |
| C | 6.548255 | 3.100388 | 3.779145 |
| C | 3.730252 | 12.21716 | 5.135515 |
| C | 7.872648 | 13.44781 | 3.396371 |
| C | 2.279861 | 7.831675 | 3.944483 |
| C | 1.479775 | 11.0427 | 1.943321 |
| C | 11.56503 | 6.88378 | 2.945891 |
| C | 8.342535 | 14.65404 | 1.338025 |
| C | 7.098271 | 14.2809 | 0.8498 |
| C | 0.460474 | 8.771189 | 2.051776 |
| C | 10.58796 | 3.571185 | 5.823469 |
| C | 8.721985 | 14.22257 | 2.60047 |
| C | 6.591525 | 13.09473 | 2.905393 |
| C | 6.217031 | 13.4955 | 1.599119 |
| C | 12.71194 | 4.767933 | 6.077249 |
| C | 6.513749 | 8.302323 | 7.097676 |
| C | 5.236815 | 8.905061 | 7.47625 |
| C | 7.566105 | 9.279004 | 6.836793 |
| C | 5.100104 | 10.26249 | 7.702802 |
| C | 7.399817 | 10.62711 | 7.110726 |
| C | 6.16185 | 11.17329 | 7.499955 |
| H | 7.192962 | 10.42543 | 1.969716 |
| H | 5.992212 | 9.243939 | 1.423424 |
| H | 7.418641 | 9.587297 | 0.431894 |
| H | 8.890028 | 8.588761 | 2.197925 |
| H | 7.735796 | 7.429855 | 1.592197 |
| H | 7.631519 | 8.911398 | 4.279915 |
| H | 6.274014 | 7.895004 | 3.655402 |
| H | 8.177828 | 8.237799 | 10.66593 |
| H | 6.611695 | 8.559793 | 11.41371 |
| H | 7.004565 | 9.273042 | 9.844487 |
| H | 6.619468 | 6.274973 | 10.41721 |
| H | 5.438042 | 7.302361 | 9.638985 |
| H | 8.216486 | 6.889135 | 8.40541 |
| H | 6.746104 | 5.915175 | 7.982107 |
| H | 7.752508 | 1.317284 | 7.722339 |
| H | 7.157707 | 1.853255 | 9.299455 |
| H | 6.079492 | 0.989806 | 8.190531 |
| H | 13.40923 | 8.067818 | 8.906955 |
| H | 12.63104 | 6.742246 | 9.782752 |
| H | 13.37666 | 6.450244 | 8.202528 |
| H | 4.474978 | 2.939813 | 8.611272 |
| H | 5.66019 | 3.89165 | 9.500722 |
| H | 4.883513 | 4.565402 | 8.057008 |
| H | 9.736613 | 8.804546 | 8.569175 |
| H | 10.52373 | 8.095423 | 9.987369 |
| H | 11.28403 | 9.437272 | 9.136946 |
| H | 4.429231 | 12.34952 | 1.613703 |
| H | 3.462317 | 1.734117 | 4.30289 |
| H | 1.193867 | 10.02193 | 5.570542 |
| H | 1.684851 | 11.4399 | 6.531004 |
| H | 0.877817 | 11.65783 | 4.984109 |
| H | 9.430288 | 6.416057 | 2.949486 |
| H | 10.36224 | 5.617172 | 1.693934 |
| H | 10.47809 | 5.02095 | 3.351834 |
| H | 4.996882 | 2.385813 | 2.47618 |
| H | 6.687369 | 5.443694 | 2.273363 |
| H | 7.550017 | 4.757878 | 0.887618 |
| H | 5.915312 | 4.204692 | 1.273425 |
| H | 4.357606 | 15.11744 | 0.291289 |
| H | 2.96907 | 14.03931 | 0.488493 |
| H | 3.75866 | 14.74126 | 1.909927 |
| H | 13.05814 | 5.343872 | 3.36997 |
| H | 12.87256 | 5.71732 | 1.651964 |
| H | 13.74304 | 6.838724 | 2.71557 |
| H | -0.17745 | 11.85684 | 3.134787 |
| H | 0.262718 | 12.83257 | 1.724327 |
| H | -0.64769 | 11.33476 | 1.517879 |
| H | 12.53264 | 9.846863 | 7.370399 |
| H | 5.746275 | 11.63961 | -0.38652 |
| H | 4.116796 | 12.1727 | -0.83108 |
| H | 5.496591 | 13.22524 | -1.12854 |
| H | 7.846041 | 14.8739 | 5.784724 |
| H | 9.006094 | 13.86363 | 6.664985 |
| H | 9.533925 | 14.8028 | 5.266817 |
| H | 10.42201 | 12.59526 | 4.122916 |
| H | 9.955037 | 11.7479 | 5.596729 |
| H | 9.350454 | 11.1968 | 4.026857 |
| H | 9.603077 | 1.346427 | 4.433832 |
| H | 9.736541 | 1.120469 | 6.172614 |
| H | 8.135331 | 1.27128 | 5.415045 |
| H | -0.38901 | 6.7909 | 2.08103 |
| H | 1.673745 | 6.111508 | 6.052442 |
| H | 3.126861 | 6.420371 | 7.005013 |
| H | 1.961294 | 7.726519 | 6.708167 |
| H | 7.290161 | 3.754962 | 7.696905 |
| H | 1.115742 | 10.38343 | -0.10587 |
| H | 1.922038 | 11.95076 | 0.011763 |
| H | 2.827338 | 10.45621 | 0.327861 |
| H | 7.572993 | 12.45599 | 5.245882 |
| H | 8.403215 | 3.808526 | 3.019373 |
| H | 5.677838 | 14.87936 | 4.26296 |
| H | 3.909138 | 14.86645 | 4.426482 |
| H | 4.916034 | 14.54045 | 5.831695 |
| H | 1.239292 | 5.964909 | 3.749621 |
| H | 10.85891 | 6.590961 | 8.065194 |
| H | 6.90607 | 1.77385 | 1.287179 |
| H | 8.526481 | 2.417329 | 1.001627 |
| H | 8.215231 | 1.381009 | 2.405893 |
| H | 3.950493 | 7.992135 | 5.257602 |
| H | 4.147302 | 2.068007 | 6.651949 |
| H | 13.02973 | 10.69934 | 5.101083 |
| H | 4.515941 | 6.280291 | 3.512294 |
| H | 4.682154 | 5.633029 | 5.155986 |
| H | 3.289433 | 5.198065 | 4.174937 |
| H | 12.51656 | 9.294614 | 3.13256 |
| H | 3.271981 | 12.83731 | 5.900353 |
| H | 2.259154 | 11.61487 | 2.454291 |
| H | 11.45964 | 7.652417 | 2.168331 |
| H | 9.010556 | 15.26637 | 0.738587 |
| H | 6.799263 | 14.60525 | -0.14312 |
| H | -0.24163 | 9.133875 | 1.305291 |
| H | 11.28523 | 2.748754 | 5.953723 |
| H | 9.700285 | 14.49668 | 2.987496 |
| H | 13.15879 | 3.986629 | 5.456218 |
| H | 13.20868 | 5.714772 | 5.863187 |
| H | 12.91604 | 4.500977 | 7.120187 |
| H | 4.385262 | 8.254988 | 7.645914 |
| H | 8.566013 | 8.947403 | 6.559828 |
| H | 4.129221 | 10.63703 | 8.019285 |
| H | 8.261416 | 11.2787 | 7.001776 |
| H | 6.051299 | 12.22869 | 7.7155 |
| H | 6.251193 | 7.405574 | 5.998707 |
|  |  |  |  |
|  |  |  |  |

## 1.7 Supplementary References

1. Tilley, T. D. Ph.D. Thesis, University of California, Berkeley, 1982.

2. Stender, M. et al*.* The synthesis and structure of lithium derivatives of the sterically encumbered β-diketiminate ligand [{(2,6-Pri_2_H_3_C_6_)N(CH_3_)C}2CH]^−^, and a modified synthesis of the aminoimine precursor. *Dalton Trans.* **23**, 3465-3469 (2001).

3. Gulmine, J. V., Janissek, P. R., Heise, H. M., Akcelrud, L. Polyethene characterization by FTIR. *Polym. Test.* **21**, 557-563 (2002).

4. Kovács, R. L. et al. Surface characterization of plasma-modified low density polyethylene by attenuated total reflectance fourier-transform infrared (ATR-FTIR) spectroscopy combined with chemometrics. *Polym. Test*. **96**, 107080 (2021).

5. Spina, F. et al. Low density polyethylene degradation by filamentous fungi. *Environ. Pollut.* **274**, 116548 (2021).

6. Kelly, K., Brown, G. & Anthony, S. Quantifying CTFE content in FK-800 using ATR-FTIR and time to peak crystallization. *Int. J. Polym. Anal. Charact.* **25**, 621-633 (2020).

7. Suman, T. Y. et al*.* Characterization of petroleum-based plastics and their absorbed trace metals from the sediments of the Marina Beach in Chennai, India. *Environ. Sci.* *Eur.* **32**, 110 (2020).

4. Spek, A. L. Planton Squeeze: a tool for the calculation of the disordered solvent contribution to the calculated structure factors. *Acta Crystallogra. C*, **C71**, 9-18 (2015).

5. Becke, A. D. *J. Chem. Phys.*, **98**, 5648 (1993).

6. Burke, K., Perdew, J. P., Wang, Y. In Electronic Density Functional Theory: Recent Progress and New Directions; J. F. Dobson, G. Vignale, M. P. Das, Eds.; Plenum: New York (1998).

7. Gaussian 09 Revision D.01, Frisch, M. J. et al. Gaussian, Inc., Wallingford CT (2016).

8. Küchle, W., Dolg, M., Stoll, H., Preuss, H. *J. Chem. Phys.* **100**, 7535 (1994).
9. Cao, X., Dolg, M., Stoll, H. *J. Chem. Phys.* **118**, 487 (2003).
10. Cao, X., Dolg, M. *J. Molec. Struct*. (Theochem), **673**, 203 (2004).

11. (a) McLean, A. D., Chandler, G. S. *J. Chem. Phys.* **72**, 5639 (1980). (b) Hehre, W. J., Ditchfield, R., Pople, J. A. *J.Chem. Phys.* **56**, 2257 (1972).
